# Supplementary material for: A modelling assessment of short- and medium-term risks of programme interruptions for gambiense human African trypanosomiasis in the DRC
Source: PLoS Negl Trop Dis. 2023 Apr 28;17(4):e0011299. doi: 10.1371/journal.pntd.0011299 (PMC10171604; doi:10.1371/journal.pntd.0011299)
Supplement: S1 Text — An expansion of the mathematical modelling methodology. (PDF) [file pntd.0011299.s001.pdf]

A modelling assessment of short- and medium-term risks of programme interruptions for *gambiense* human African trypanosomiasis in the DRC

Supporting information – S1 Text: Methods

Ching-I Huang[1,2,*,◑], Ronald E Crump[1,2,3], Emily H Crowley[1,2], Andrew Hope[4], Paul R Bessell[5], Chansy Shampa[6], Erick Mwamba Miaka[6], Kat S Rock[1,2◑]

1 Zeeman Institute for System Biology and Infectious Disease Epidemiology Research, The University of Warwick, Coventry, U.K.
2 Mathematics Institute, The University of Warwick, Coventry, U.K.
3 The School of Life Sciences, The University of Warwick, Coventry, U.K.
4 Liverpool School of Tropical Medicine (LSTM), Liverpool, U.K.
5 Independent Consultant, Edinburgh, U.K.
6 Programme National de Lutte contre la Trypanosomiase Humaine Africaine (PNLTHA), Kinshasa, D.R.C.

◑These authors contributed equally to this work.
* ching-i.huang@warwick.ac.uk

This work is built on a series of studies on modelling and analyses of eliminating transmission of *gambiense* human African trypanosomiasis (gHAT) in the Democratic Republic of Congo (DRC). The series of studies includes a model fitting paper [1] and a model projections paper [2]. The key difference between the model utilised in the present study and the recent papers [1, 2] is the way it can generate new projections which allow for (1) reduction in active screening (AS) coverage, (2) lower detection rates of passive screening (PS) from both stage 1 and stage 2, and (3) suspension or delay enrollment of vector control (VC) for 2020 and 2021. There are no fundamental differences in the model itself, only the simulation of these different future scenarios. To aid the reader of the present study, much of the same model information is provided here. Note that the gHAT model is fitted to individual health zone HAT Atlas data [3] independently and health zones with fewer than 10 data points (number of screening events plus non-zero passive cases) are excluded in the previous and this studies.

S1.1 The compartmental gHAT model

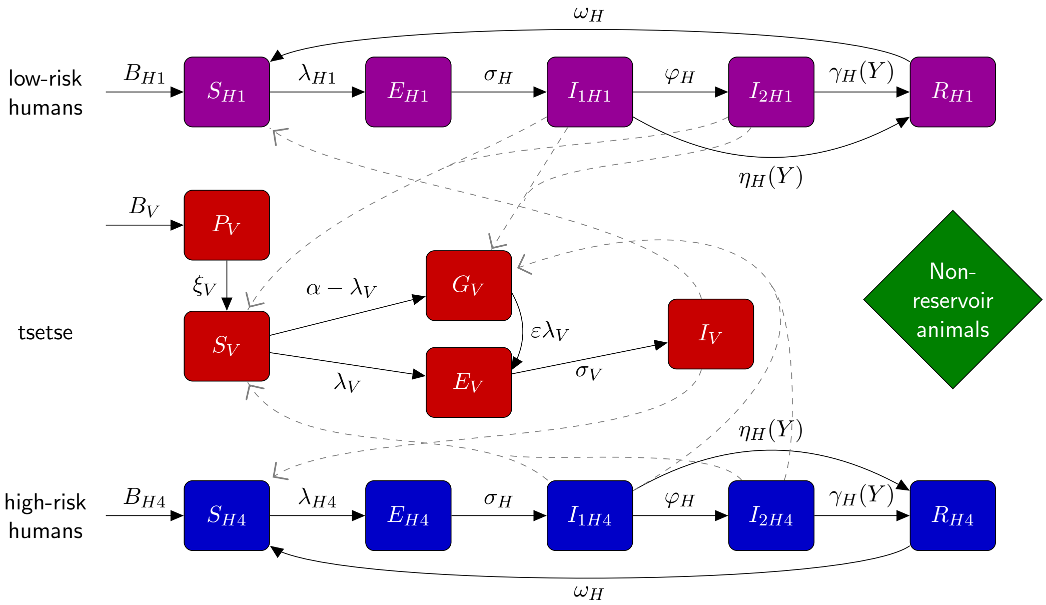

Fig A. Illustration of compartmental gHAT model. Multi-host gHAT model is composed of one host species able to confer gHAT (humans), a further non-reservoir species (others) and tsetse. After incubation period, infected human hosts follow the progression which includes infectious stage 1 disease, I_{1H}, infectious stage 2 disease, I_{2H}, and non-infectious (due to hospitalisation) disease, R. Pupal stage tsetse, P_V, emerge into unfed adults. Unfed tsetse are susceptible, S_V, and following a blood-meal become either exposed, E_V, or have reduce susceptibility to the trypanosomes, G_V. Tsetse select their blood-meal from one of the host types dependant upon innate feeding preference and relative host abundance. High-risk humans are more likely to receive bites than low-risk humans. Any blood-meals taken upon "other" hosts do not result in infection. The transmission of infection between humans and tsetse is shown by grey paths. This figure is adapted from the original model schematic [4], which was published under a CC-BY licence.

The gHAT model we considered in this study is a variant "Model 4" of the Warwick model presented in the literature [1, 4–6], starting with Rock et al. [4]. An illustration in Fig. A and mathematical descriptions by Equation (1) highlight that this compartmental multi-host model captures improvements in passive screening at fixed health facilities ($\eta_H(Y)$ for stage 1 and $\gamma_H(Y)$ for stage 2) and systematic non-participation of high-risk groups (subscript $H4$, are r-fold more likely to receive bites and never show up in active screenings) in the population. This participating structure, i.e. low-risk humans randomly participate in active screening and high-risk humans are assumed to never participate, is supported by data in previous model fits [1].

Human hosts are modelled by the SEIIRS model with two infectious compartments, stage 1 disease, I_{1H}, and stage 2 disease, I_{2H}. Vectors are modelled by using compartments for appropriately modelling tsetse when used in a host-vector model with disease [5]. Pupal stage tsetse, P_V, emerge into unfed susceptible adults, S_V, and following a blood-meal become either exposed, E_V, or have reduced susceptibility to the *Trypanosoma brucei gambiense* parasites, G_V - this effect is known as the teneral phenomenon. Following an infection, tsetse have an extrinsic incubation period (EIP) before becoming onwardly infectious. To incorporate a more realistic EIP distribution, there are three exposed classes,

E_{1V}, E_{2V}, E_{3V}, which result in a gamma-distributed EIP (rather than an exponential with only one). Tsetse bites are assumed to be taken on humans or non-reservoir animals. However, the non-reservoir animal species do not need to be explicitly modelled, i.e. this model variant does not include tsetse to non-human animal transmission.

Tsetse select their blood-meal from one of the host types dependant upon innate feeding preference and relative host abundance. We assume tsetse preferentially feed on humans with a probability f_H which is taken to be 0.09 [7]. The proportion of tsetse bites taken on low-risk and high-risk humans are f_1 and f_4, depending on the relative availability/attractiveness and the relative abundance of two risk groups. High-risk humans are assumed to be r-fold more likely to receive bites, i.e. $s_1 = 1$ and $s_4 = r$. Therefore, f_i's can be calculated using $f_i = \dfrac{s_i N_{Hi}}{\sum_j s_j N_{Hj}}$.

In order to reduce the dimensionality of our ODE system (by one), the vector equations are non-dimensionalised using the scaling N_H/N_V, where N_H is the total human population, and N_V is the tsetse population size. This results in a new non-dimensionalised parameter, m_{eff}, which is $\dfrac{p_H N_V}{N_H}$ appearing in host equations (p_H is the probability of a human being infected by a single infectious bloodmeal) and is referred to as the *effective vector density.*

$$
\text{Humans}\left\{
\begin{aligned}
\frac{\mathrm{d}S_{Hi}}{\mathrm{d}t} &= \mu_H N_{Hi} + \omega_H R_{Hi} - \alpha m_{\text{eff}} f_i \frac{S_{Hi}}{N_{Hi}} I_V - \mu_H S_{Hi} \\
\frac{\mathrm{d}E_{Hi}}{\mathrm{d}t} &= \alpha m_{\text{eff}} f_i \frac{S_{Hi}}{N_{Hi}} I_V - (\sigma_H + \mu_H) E_{Hi} \\
\frac{\mathrm{d}I_{1Hi}}{\mathrm{d}t} &= \sigma_H E_{Hi} - (\varphi_H + \eta_H(Y) + \mu_H) I_{1Hi} \\
\frac{\mathrm{d}I_{2Hi}}{\mathrm{d}t} &= \varphi_H I_{1Hi} - (\gamma_H(Y) + \mu_H) I_{2Hi} \\
\frac{\mathrm{d}R_{Hi}}{\mathrm{d}t} &= \eta_H(Y) I_{1Hi} + \gamma_H(Y) I_{2Hi} - (\omega_H + \mu_H) R_{Hi}
\end{aligned}
\right.
$$

$$
\text{Tsetse}\left\{
\begin{aligned}
\frac{\mathrm{d}P_V}{\mathrm{d}t} &= B_V N_H - (\xi_V + \tfrac{P_V}{K}) P_V \\
\frac{\mathrm{d}S_V}{\mathrm{d}t} &= \xi_V \mathbb{P}(\text{survive pupal stage}) P_V - \alpha S_V - \mu_V S_V \\
\frac{\mathrm{d}E_{1V}}{\mathrm{d}t} &= \alpha\big(1 - f_T(t)\big) p_V \big(\textstyle\sum_i f_i \dfrac{(I_{1Hi} + I_{2Hi})}{N_{Hi}} + f_A \dfrac{I_A}{N_A}\big)(S_V + \varepsilon G_V) \\
&\qquad -(3\sigma_V + \mu_V + \alpha f_T(t)) E_{1V} \\
\frac{\mathrm{d}E_{2V}}{\mathrm{d}t} &= 3\sigma_V E_{1V} - (3\sigma_V + \mu_V + \alpha f_T(t)) E_{2V} \\
\frac{\mathrm{d}E_{3V}}{\mathrm{d}t} &= 3\sigma_V E_{2V} - (3\sigma_V + \mu_V + \alpha f_T(t)) E_{3V} \\
\frac{\mathrm{d}I_V}{\mathrm{d}t} &= 3\sigma_V E_{3V} - (\mu_V + \alpha f_T(t)) I_V \\
\frac{\mathrm{d}G_V}{\mathrm{d}t} &= \alpha(1 - f_T(t))\Big(1 - p_V\big(\textstyle\sum_i f_i \dfrac{(I_{1Hi} + I_{2Hi})}{N_{Hi}} + f_A \dfrac{I_A}{N_A}\big)\Big) S_V \\
&\qquad -\alpha\Big(f_T(t) + (1 - f_T(t)) p_V \varepsilon \big(\textstyle\sum_i f_i \dfrac{(I_{1Hi} + I_{2Hi})}{N_{Hi}} + f_A \dfrac{I_A}{N_A}\big)\Big) G_V \\
&\qquad -\mu_V G_V
\end{aligned}
\right.
\tag{1}
$$

S1.2 Model fitting

S1.2.1 Parameters

Table A provides the estimates of fixed parameters available in the literature used in the previous gHAT model [1,4,5]. The parameters fitted during the model fitting are defined in Table B. Prior and posterior distributions of all fitted parameters in Kwamouth health zone are summarised in Table B and Fig. B. Posterior distributions for these parameters are estimated using MCMC methods [1] and are available in our graphical user interface at https://hatmepp.warwick.ac.uk/fitting/v2/, or can be downloaded in Open Science Framework at https://osf.io/ck3tr/.

Table A. Model parametrisation (fixed parameters). Notation, brief description, and the used values for fixed parameters.

Notation	Description	Value	
N_H	Total human population size in 2015	Fixed for each health zone	[8]
μ_H	Natural human mortality rate	5.4795×10^{-5} days^{-1}	[9]
B_H	Total human birth rate	$= \mu_H N_H$	
σ_H	Human incubation rate	0.0833 days^{-1}	[10]
φ_H	Stage 1 to 2 progression rate	0.0019 days^{-1}	[11,12]
ω_H	Recovery rate or waning-immunity rate	0.006 days^{-1}	[13]
Sens	Active screening diagnostic sensitivity	0.91	[14]
B_V	Tsetse birth rate	0.0505 days^{-1}	[5]
ξ_V	Pupal death rate	0.037 days $^{-1}$	
K	Pupal carrying capacity	$= 111.09 N_H$	[5]
$\mathbb{P}$(pupating)	Probability of pupating	0.75	
μ_V	Tsetse mortality rate	0.03 days^{-1}	[10]
σ_V	Tsetse incubation rate	0.034 days^{-1}	[15,16]
α	Tsetse bite rate	0.333 days^{-1}	[17]
p_V	Probability of tsetse infection per infectious bite	0.065	[10]
ε	Reduced non-teneral susceptibility factor	0.05	[4]
f_H	Proportion of blood-meals on humans	0.09	[7]
disp_{act}	Overdispersion parameter for active detection	4×10^{-4}	[1]
$\text{disp}_{\text{pass}}$	Overdispersion parameter for passive detection	2.8×10^{-5}	[1]

[1] Value of B_V is chosen to maintain constant population size without interventions.
[2] Value of K is chosen to reflect the observed bounce back rate.

Table B. Model parametrisation (posteriors of fitted parameters). Notation, brief description, information on prior distributions, and representative percentiles of the posterior distributions (Kwamouth health zone) for fitted parameters.

Notation	Description	Prior distribution	Posterior (median [95% CI])
R_0	Basic reproduction number (NGM approach)	$1 + \text{Exp}(10)$	1.09 [1.06, 1.14]
r	Relative bites taken on high-risk humans	$1 + \Gamma(3.68, 1.09)$	6.61 [3.15, 10.75]
k_1	Proportion of low-risk people	$\text{B}(16.97, 3.23)$	0.90 [0.82, 0.95]
$b_{\gamma_H^{\text{pre}}}$	Pre-1998 relative exit rate from stage 2 factor	$\text{B}(1, 1)$	0.92 [0.73, 1.00]
η_H^{post}	Post-1998 treatment rate from stage 1 (days^{-1})	$\Gamma\left(3.54, 5.32 \times 10^{-5}\right)$	1.24 $\times 10^{-4}$ [0.60, 2.74] $\times 10^{-4}$
γ_H^{post}	Post-1998 exit rate from stage 2 (days^{-1})	$\Gamma(2.45, 0.00192)$	1.88 $\times 10^{-3}$ [0.46, 5.42] $\times 10^{-3}$
Spec	Active screening diagnostic specificity	$0.998 + (1 - 0.998)\,\text{B}(7.23, 2.41)$	0.9991 [0.9987, 0.9997]
u	Proportion of stage 2 passive cases reported	$\text{B}(20, 40)$	0.27 [0.18, 0.40]
d_{change}	Midpoint year for passive improvement	$2000 + (2017 - 2000)\,\text{B}(5, 6)$	2005.8 [2004.4, 2007.3]
$\eta_{H_{\text{amp}}}$	Relative improvement in passive stage 1 detection rate	$\Gamma(2.013, 1.049)$	2.52 [0.92, 5.46]
$\gamma_{H_{\text{amp}}}$	Relative improvement in passive stage 2 detection rate	$\Gamma(1.001, 5)$	0.51 [0.24, 0.97]
d_{steep}	Speed of improvement in passive detection rate (years^{-1})	$\Gamma(39.57, 0.0270)$	0.94 [0.68, 1.29]

[1] Exp(.), Γ(.) and B(.) in prior distributions are the exponential, gamma (shape, scale) and beta distributions.
[2] See Equation (2) for improved passive detections formulated by d_{change}, $\eta_{H_{\text{amp}}}$, $\gamma_{H_{\text{amp}}}$ and d_{steep}.

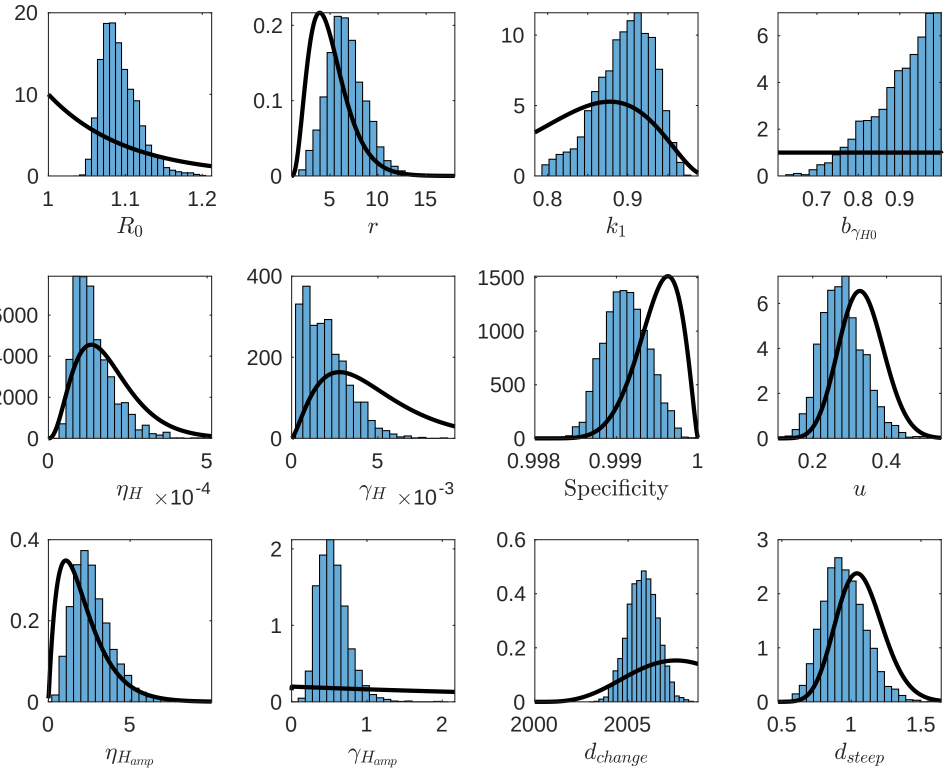

Fig B. Prior and posterior distributions of all fitted parameters in Kwamouth health zone. Black lines are the prior distributions of fitted parameters. Blue histograms from 2,000 posteriors show the posterior distributions from fitting our gHAT model to data.

S1.2.2 Formulation of improved passive detections

Previous analysis on provincial-level staged data (Lumbala *et al.* for 2000–2012 [18] and WHO HAT Atlas data 2015–2016 [3]) indicated that improved passive detection has happened across Kwilu, Mai Ndombe, Kwango and Kongo Central provinces [1]. In our model, improved passive detections in year Y were formulated by logistic functions

$$\begin{aligned} \eta_H(Y) &= \eta_H^{\text{post}}\left[1 + \frac{\eta_{H_{\text{amp}}}}{1+\exp\left(-d_{\text{steep}}(Y - d_{\text{change}})\right)}\right], \\ \gamma_H(Y) &= \gamma_H^{\text{post}}\left[1 + \frac{\gamma_{H_{\text{amp}}}}{1+\exp\left(-d_{\text{steep}}(Y - d_{\text{change}})\right)}\right]. \end{aligned} \tag{2}$$

Parameter definitions and their priors are provided in Table B, posterior characteristics for Kwamouth health zone are summarised in Table B and Fig. B. N.B. It was assumed that improvements in both stages shared the same midpoint year and speed of improvement within a health zone. However, the amplitude of variation in each health zone came from the fitting of health-zone-specific data (see Fig. C).

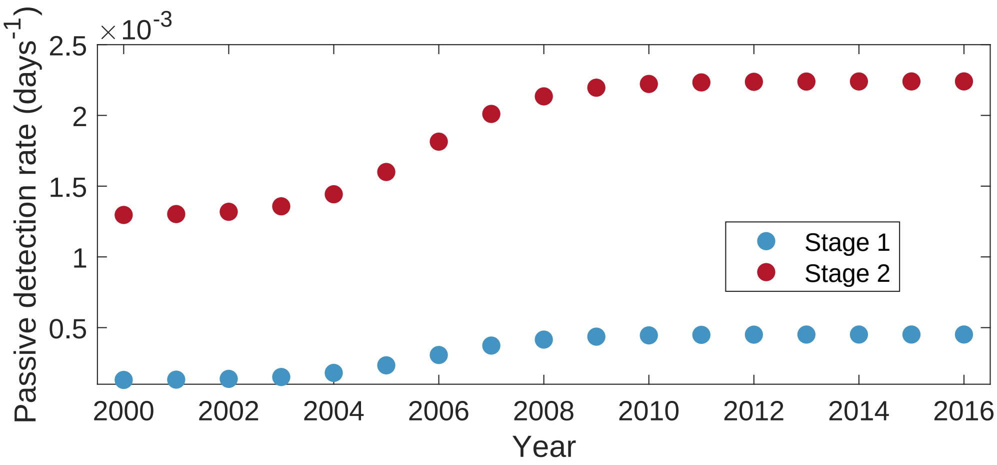

Fig C. Yearly passive detection rates in Kwamouth health zone. The values of passive detection rates shown in this figure are calculated based on Equation (2) using the median values of each parameter from its posterior. Blue and red circles denote the passive detection rates of stage 1 and 2 respectively. Note that stage 2 detection rate here is $(1-u)\gamma_H(Y)$, where u is the proportion of stage 2 passive cased reported and $\gamma_H(Y)$ is a combined rate of exiting stage 2 including detection and unreported deaths.

S1.2.3 Formulation of additional tsetse mortality under vector control measures

The tsetse dynamics without gHAT infections can be described by

$$\begin{aligned} \frac{\mathrm{d}P_V}{\mathrm{d}t} &= B_V N_H - (\xi_V + \tfrac{P_V}{K})P_V, \\ \frac{\mathrm{d}S_V}{\mathrm{d}t} &= \xi_V \mathbb{P}(\text{survive pupal stage})P_V - \alpha S_V - \mu_V S_V, \\ \frac{\mathrm{d}G_V}{\mathrm{d}t} &= \alpha(1 - f_T(t))S_V - \alpha f_T(t) G_V - \mu_V G_V. \end{aligned} \tag{3}$$

In our tsetse model, we assumed the efficacy of Tiny Target (f_T) reduces rapidly after 4 months due to the potentials of being washed away by rain or covered by mud. Thus, a sigmoid function, which has a characteristic S-shaped curve, was used to describe the time dependent probability of both hitting a

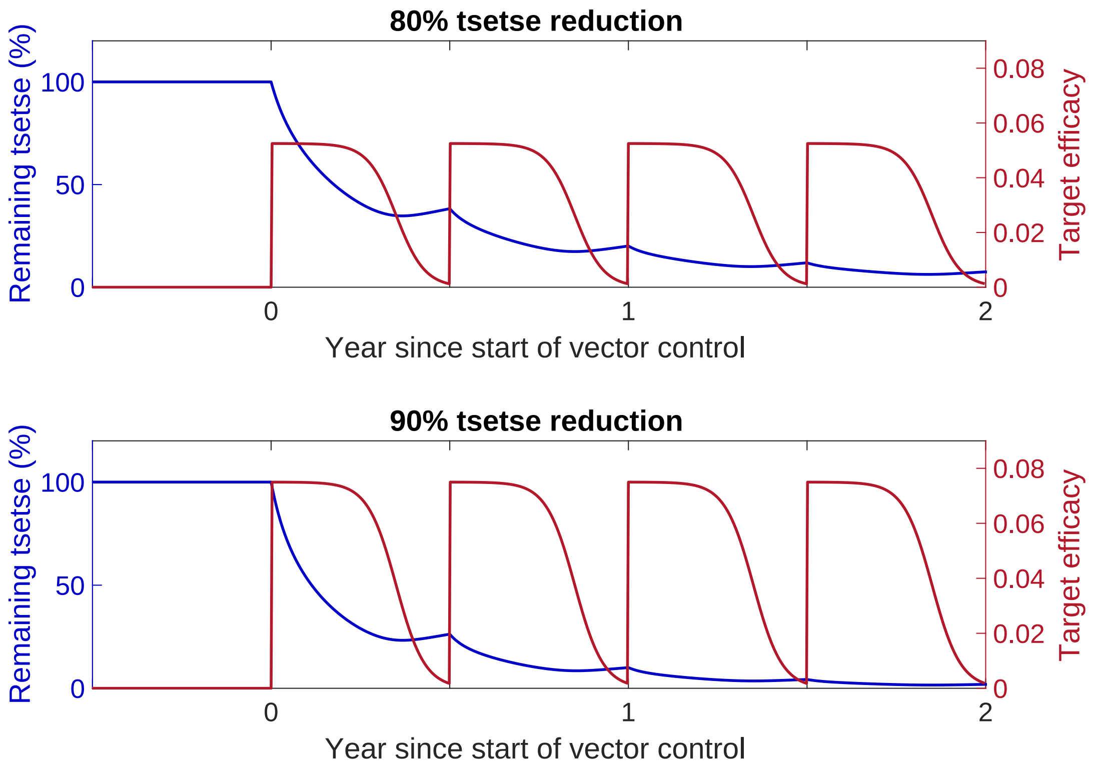

Fig D. Trends of tsetse populations and target efficacy under vector control. Red curves show Tiny Target efficacy ($f_T(t)$ defined in Equation (4)) for bi-annual deployments. Blue curves present the corresponding tsetse dynamics for 80% (top panel) and 90% (bottom panel) annual tsetse reduction caused by vector control.

target and dying when bi-annual deployments occur at the beginning and middle of the year. The efficacy of Tiny Target,

$$f_T(t) = f_{\max}\left(1 - \frac{1}{1+\exp(-0.068(\mathrm{mod}(t, 182.5) - 127.75))}\right), \tag{4}$$

where $f_{\max}$ is chosen such that the tsetse population after one year is at the observed/assumed percentage reduction. For DRC-specific settings, i.e. two deployments each year, we used $f_{\max} = 0.0525$ for an 80% reduction (for Masi Manimba, Bandundu, Kikongo, Kwamouth, Bokoro, Bolobo, Bulungu, Mokala, Mushie, and Yumbi), and $f_{\max} = 0.0750$ for a 90% reduction (for Yasa Bonga). Tsetse dynamics and Tiny Target efficacy for 80% and 90% reductions are shown in Fig. D.

S1.2.4 Fits to historical case data

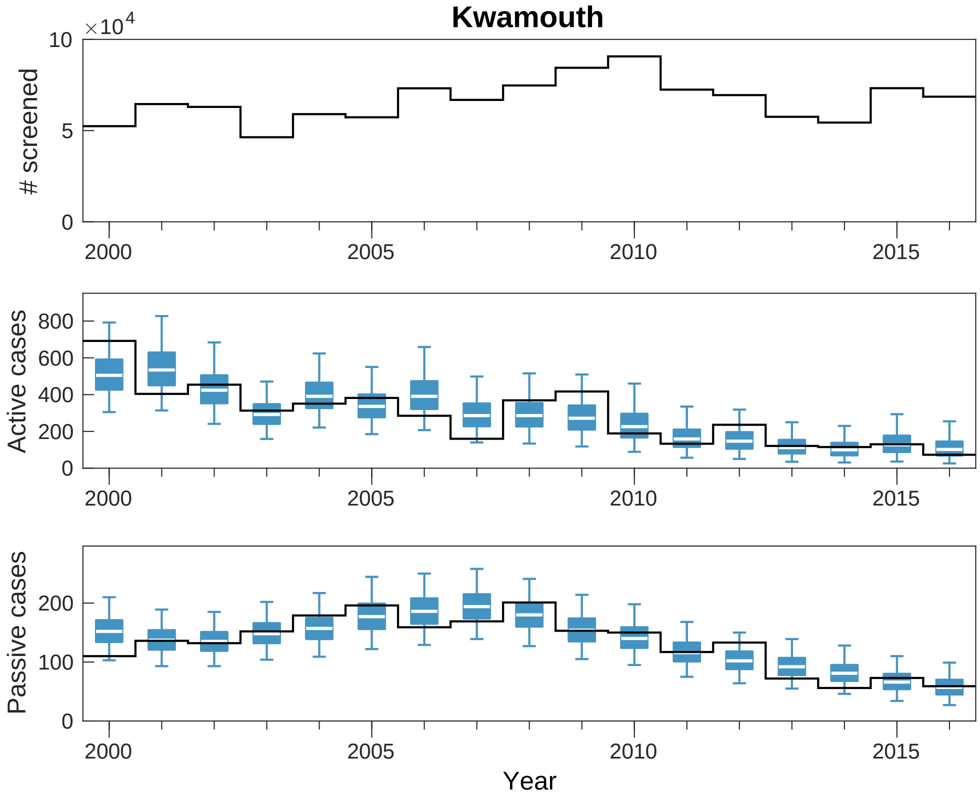

Fig E. Model fits to the observed trends in active and passive case detections over time for Kwamouth health zone. There are n = 10,000 independent samples, 10 from each of 1,000 independent samples from the joint posterior distributions of the fitted model parameters. Black lines and box plots indicate data and model fits during the data period (2000–2016). Box plots summarise parameter and observational uncertainty. The lines in the boxes present the medians of predicted results. The lower and upper bounds of the boxes indicate 25th and 75th percentiles. The minimum and maximum values are 2.5th and 97.5th percentiles and therefore whiskers cover 95% prediction intervals. Time series plots of all analysed health zones are available in the graphical user interface at https://hatmepp.warwick.ac.uk/fitting/v2/.

S1.3 Model projections

S1.3.1 Active screening

Screening data is aggregated by year and the exact dates and frequencies of conducting AS are unknown (see top panel in Fig. E), therefore some assumptions were made as to when AS takes place. Our model assumed only low-risk humans participate in AS and used the ratio of assumed number of people screened (N_{AS}) and the number of low-risk humans ($k_1 N_H$) to decide the frequency of AS each year. When screening numbers were smaller than potential participants ($N_{AS} < k_1 N_H$), a single AS event was assumed to take place at the beginning of those years. On the other hand, multiple AS events were evenly distributed over the time of the corresponding years, i.e. a second AS event in July when $k_1 N_H < N_{AS} \leq 2k_1 N_H$; a second AS event in May and a third AS event in September when $2k_1 N_H < N_{AS} \leq 3k_1 N_H$; etc.

S1.3.2 Uncertainty

Simulations for each scenario (including the baseline) in each health zone were performed based on 1,000 sets of parameters (or model realisations). Observation uncertainty was considered by drawing 10 random samples from the predicted mean dynamics for each realisation. A beta-binomial distribution in which an overdispersion parameter ρ was introduced to the binomial distribution was used to account for larger variance than the binomial. The probability of obtaining m successes out of n trials with probability p and overdispersion parameter ρ is

$$\text{BetaBin}(m; n, p, \rho) = \frac{\Gamma(n+1)\Gamma(m+a)\Gamma(n-m+b)\Gamma(a+b)}{\Gamma(n-m+1)\Gamma(n+a+b)\Gamma(a)\Gamma(b)}, \tag{5}$$

where $a = p(1/\rho - 1)$ and $b = a(1-p)/p$.

In other words, main observable outputs (e.g. active and passive cases each year) were predicted by 10,000 samples incorporating both parameter and observation uncertainties, but unobservable outputs (such as new infections and the year of elimination of transmission) were predicted directly from the 1,000 model realisations without sampling (i.e. parameter uncertainty but no observation uncertainty). We tried to represent this uncertainty in a variety of ways:

- Time series box plots were used to display statistical summaries of model predictions – the median (the middle line in each box), the lower and upper quartiles (the edges of each box showing 50% prediction intervals), and 95% prediction intervals (extended whiskers containing the middle 95% of outputs).
- The median year of elimination of transmission was used to indicate the estimated elimination year for a series of model predictions because neither extreme values (outliers) nor truncation of simulation will affect the estimates.
- The probability of elimination of transmission by years were used to set a certainty (or a confidence level) of achieving desirable outcome (i.e. EoT in this paper) at specific time point based on a series of model predictions.

S1.3.3 Tsetse bounce back in interruption scenarios

In the 9-month *No AS or VC and reduced PS* scenario, it was assumed that there was no VC deployment in Yasa Bonga, Masi Manimba, Bandundu, Kikongo, and Kwamouth in mid-2020. The suspension continued until the end of 2021 in the 21-month interruption scenario. During the period of interruption, we assumed the existing Tiny Targets from the latest deployment in the beginning of 2020 keep working with extremely low efficacy, and therefore, remaining tsetse can repopulate its population. Fig. F shows the bounce back of tsetse population from the absence of deploying new Tiny Targets for three periods of interruption starting from April 2020. The level of bounce back not only depends on the duration of interruption but also the remaining tsetse at the beginning of interruption.

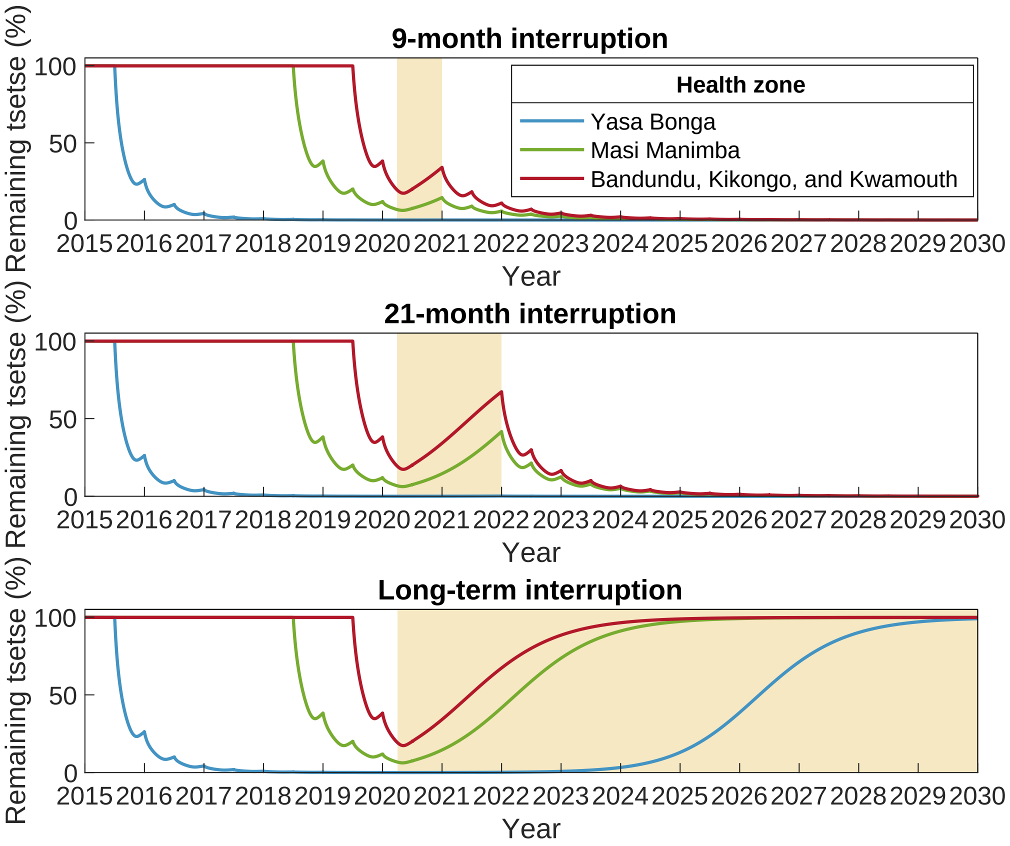

Fig F. Tsetse bounce back during the suspension of VC Three panels show three length of VC interruption, 9-month (top), 21-month (middle), and long-term (i.e. never restart, bottom). In each panel, modelled tsetse dynamics is presented by colour curves associating with different VC starting years (blue for Yasa Bonga, green for Masi Manimba, and red for Bandundu, Kikongo, and Kwamouth in which VC started from 2015.5, 2018.5, and 2019.5 respectively). Colour shaded area highlights three length of interruption periods starting from April 2020.

S1.3.4 Proxy for elimination of transmission

The gHAT model we used here is a deterministic model described by ODEs with transition rates between compartments. In the deterministic model, variables such as new infections, new cases and deaths can be non-integer and their values are continuous. The stochastic model, on the other hand, has dynamics driven by randomly occurring events with associated probabilities and its variables capture the discrete nature of the population. Despite good agreements on the mean dynamics in both models, even at very low prevalence, the dynamics at the endgame are different. Because of the continuous nature of deterministic dynamics, the number of infected people asymptotes to zero rather than reaching it unlike the stochastic model. In this paper, an artificial elimination of transmission (EoT) threshold i.e. one new infection per health zone per year was applied to new infections to determine whether EoT has been achieved or not. Other values of EoT thresholds, such as one new infection per 100,000 or per 1,000,000 people per year, can be found in the literature [19, 20]. Large variation in EoT threshold highlights the difficulty in choosing a proper threshold reflecting the reality. More detailed comparison between stochastic and deterministic model variants will be needed in the future to ensure robustness of year of EoT estimates arising from such a proxy threshold.

S1.4 Study areas

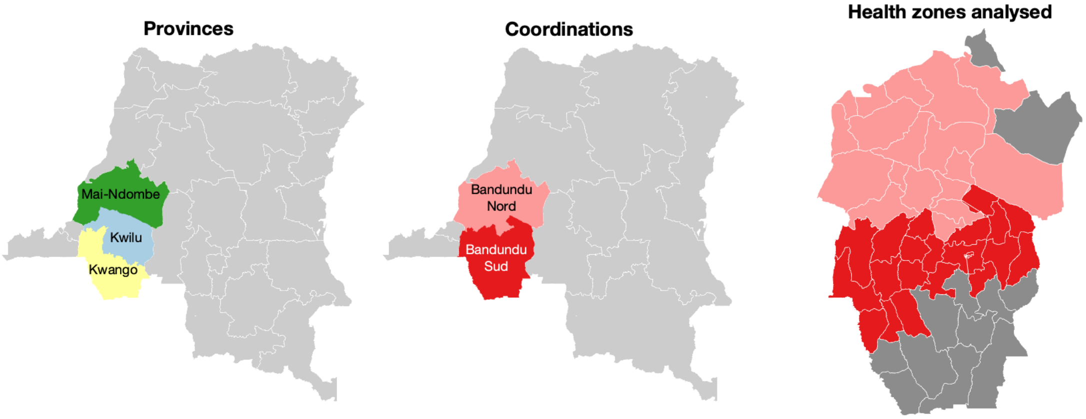

Fig G. Maps of the DRC indicating our study areas. The province map (left) highlights the three provinces covered by our study: Mai-Ndombe (in green), Kwilu (in light blue), and Kwango (in yellow) provinces. The coordination map (middle) shows our study areas mapped onto two coordinations: Bandundu Nord (in pink) and Bundundu Sud (in red) coordinations. The national programme PHLTHA-DRC makes annual plans on vertical interventions such as active screening and vector control at the coordination-level. Areas shaded by light grey in the province and coordination maps are not considered in this study. The health zone map (right) summaries areas at the geographical scale in which our analyses carried out. Coloured health zones are those included in this study (pink and red for the Bandundu Nord and Sud coordinations respectively). Health zone in dark grey are excluded in this paper due to insufficient data points for model fitting in the previous study [1]. Shapefiles used to produce these maps were provided by Nicole Hoff and Cyrus Sinai under a CC-BY licence (current versions can be found at https://data.humdata.org/dataset/drc-health-data).

S1.5 Computational specifications and runtime

Our code, available in Open Science Framework at https://osf.io/gur7c/, has been tested and run in MATLAB R2018b, R2021b, and R2022b on Windows, Linux and Mac operating systems. Initial runs of model fitting for a single health zone take 12–22 hours of elapsed time depending on data quality (the number of data points present and possibly missing active screening numbers) and intervention history (which dictates the number of fitted parameters, e.g. improved passive detection has four extra parameters) on our cluster. In a subset of health zones additional running is required to ensure that the two MCMC chains have converged to the same volume of parameter space and to achieve our target effective sample size of 1,000. In this situation, the total elapsed time across runs for a health zone can increase greatly (up to 3 or 4 times the initial run time). Two independent MCMC chains were run in parallel for each MCMC analysis and we assigned 3 cores (1 per chain, plus a master/controller) with 5 GB RAM per CPU for each fitting job on our cluster. The cluster is running CentOS Linux version 7.8 and has either Intel Xeon Gold 6130 (2.10GHz) or Intel Xeon Gold 5218 (2.30GHz) CPUs depending on the node used. Future projections of 1,000 realisations with 10 samples each from 2020 to 2050 for seven strategies (one baseline and six interruption scenarios) take about 20 minutes to complete on a desktop machine (MacOS, 4.2 GHz Quad-Core, 32 GB 2300 MHz DDR4).

References

1. Crump RE, Huang CI, Knock ES, Spencer SEF, Brown PE, Mwamba Miaka E, et al. Quantifying epidemiological drivers of *gambiense* human African trypanosomiasis across the Democratic Republic of Congo. PLoS Comput Biol. 2021;17:1–23.

2. Huang CI, Crump RE, Brown PE, Spencer SE, Miaka EM, Shampa C, et al. Identifying regions for enhanced control of *gambiense* sleeping sickness in the Democratic Republic of Congo. Nature Communications. 2022;13:1–11.

3. Simarro PP, Cecchi G, Jannin JG. The Atlas of human African trypanosomiasis: a contribution to global mapping of neglected tropical diseases. International Journal of Health Geographics. 2010;9:57.

4. Rock KS, Torr SJ, Lumbala C, Keeling MJ. Quantitative evaluation of the strategy to eliminate human African trypanosomiasis in the Democratic Republic of Congo. Parasites & Vectors. 2015;8:532.

5. Rock KS, Torr SJ, Lumbala C, Keeling MJ. Predicting the impact of intervention strategies for sleeping sickness in two high-endemicity health zones of the Democratic Republic of Congo. PLoS Negl Trop Dis. 2017;11:e0005162.

6. Mahamat MH, Peka M, Rayaisse Jb, Rock KS, Toko MA, Darnas J, et al. Adding tsetse control to medical activities contributes to decreasing transmission of sleeping sickness in the Mandoul focus (Chad). PLoS Negl Trop Dis. 2017;11:e0005792.

7. Clausen PH, Adeyemi I, Bauer B, Breloeer M, Salchow F, Staak C. Host preferences of tsetse (Diptera: Glossinidae) based on bloodmeal identifications. Medical and Veterinary Entomology. 1998;12:169–180.

8. OCHA Office for the Coordination of Humanitarian Affairs. Journees Nationales de Vaccination (JNV) activities de vaccination supplementaire, RDC. Available at: https://data.humdata.org/dataset/rdc-statistiques-des-populations (accessed May 2016).

9. The World Bank. Data: Democratic Republic of Congo. Available at: https://data.worldbank.org/country/congo-dem-rep?view=chart (accessed 2015).

10. Rogers DJ. A general model for the African trypanosomiases. Parasitology. 1988;97:193–212.

11. Checchi F, Filipe JAN, Barrett MP, Chandramohan D. The natural progression of *gambiense* sleeping sickness: what is the evidence? PLoS Negl Trop Dis. 2008;2:e303.

12. Checchi F, Funk S, Chandramohan D, Haydon DT, Chappuis F. Updated estimate of the duration of the meningo-encephalitic stage in *gambiense* human African trypanosomiasis. BMC Research Notes. 2015;8:292.

13. Mpanya A, Hendrickx D, Vuna M, Kanyinda A, Lumbala C, Tshilombo V, et al. Should I get screened for sleeping sickness? A qualitative study in Kasai province, Democratic Republic of Congo. PLoS Negl Trop Dis. 2012;6:e1467.

14. Checchi F, Chappuis F, Karunakara U, Priotto G, Chandramohan D. Accuracy of five algorithms to diagnose *gambiense* human African trypanosomiasis. PLoS Negl Trop Dis. 2011;5:e1233.

15. Davis S, Aksoy S, Galvani AP. A global sensitivity analysis for African sleeping sickness. Parasitology. 2010;138:516–526.

16. Ravel S, Grebaut P, Cuisance D, Cuny G. Monitoring the developmental status of *Trypanosoma brucei gambiense* in the tsetse fly by means of PCR analysis of anal and saliva drops. Acta Tropica. 2003;88:161–165.

17. World Health Organization & WHO Expert Committee on the Control and Surveillance of Human African Trypanosomiasis (2013: Geneva, Switzerland). Control and surveillance of human African trypanosomiasis: report of a WHO expert committee. World Health Organization; 2013.

18. Lumbala C, Simarro PP, Cecchi G, Paone M, Franco JR, Kande Betu Ku Mesu V, et al. Human African trypanosomiasis in the Democratic Republic of the Congo: disease distribution and risk. International Journal of Health Geographics. 2015;14:20.

19. Rock KS, Ndeffo-Mbah ML, Castaño S, Palmer C, Pandey A, Atkins KE, et al. Assessing strategies against *gambiense* sleeping sickness through mathematical modeling. Clinical Infectious Diseases. 2018;66:S286–S292.

20. Castaño MS, Ndeffo-Mbah ML, Rock KS, Palmer C, Knock E, Mwamba Miaka E, et al. Assessing the impact of aggregating disease stage data in model predictions of human African trypanosomiasis transmission and control activities in Bandundu province (DRC). PLoS Negl Trop Dis. 2020;14:e0007976.
