# Supplementary material for: A modelling assessment of short- and medium-term risks of programme interruptions for gambiense human African trypanosomiasis in the DRC
Source: PLoS Negl Trop Dis. 2023 Apr 28;17(4):e0011299. doi: 10.1371/journal.pntd.0011299 (PMC10171604; doi:10.1371/journal.pntd.0011299)
Supplement: S2 Text — Individual time series plots for all modelled health zones and additional figures and tables summarising impacts on EoT and DALYs. (PDF) [file pntd.0011299.s002.pdf]

A modelling assessment of short- and medium-term risks of programme interruptions for *gambiense* human African trypanosomiasis in the DRC

Supporting information – S2 Text: Additional model outputs

Ching-I Huang[1,2,*,◕], Ronald E Crump[1,2,3], Emily H Crowley[1,2], Andrew Hope[4], Paul R Bessell[5], Chansy Shampa[6], Erick Mwamba Miaka[6], Kat S Rock[1,2◕]

1 Zeeman Institute for System Biology and Infectious Disease Epidemiology Research, The University of Warwick, Coventry, U.K.
2 Mathematics Institute, The University of Warwick, Coventry, U.K.
3 The School of Life Sciences, The University of Warwick, Coventry, U.K.
4 Liverpool School of Tropical Medicine (LSTM), Liverpool, U.K.
5 Independent Consultant, Edinburgh, U.K.
6 Programme National de Lutte contre la Trypanosomiase Humaine Africaine (PNLTHA), Kinshasa, D.R.C.

◕These authors contributed equally to this work.
* ching-i.huang@warwick.ac.uk

Additional results

Fig A shows summary information for the predicted total cases, new infections, disability-adjusted life years (DALYs, a measure of disease burden) and year of elimination of transmission (YEoT) for three example health zones under different interruption scenarios. This is similar to the information provided in main text Figs 2 – 4, however, Fig A displays the total impact on outputs in each health zone between 2020 to 2030 compared the time series as displayed in the main text. This figure focuses on the overall impact of the interruption scenarios.

Figs B – E show time series of model outputs for health zones that have started vector control (VC) prior to the COVID-19 pandemic, Figs F – J show those that had plans for VC from 2020 or 2021, and Figs K – AJ show those that didn't have plans for VC, respectively. Model outputs include estimated active and passive cases, underlying new infections, disability-adjusted life years (DALYs, a measure of disease burden), and predicted probability of elimination of transmission (PEoT). Across health zones with existing VC or VC plans, the trends of model outputs are similar during and after the interruption period.

During the interruption period, the general features of model outputs are:

- Fewer active cases but more DALYs
- More passive cases when PS is fully operational (*No AS* scenario only)
- Fewer passive cases when there is reduced levels of PS (*No AS and reduced PS* and *No AS or VC and reduced PS* scenarios)
- Little impact on new infections and PEoT if VC as planned (*No AS* and *No AS and reduced PS* scenarios)
- More new infections and lower PEoT if VC suspended or postponed (*No AS or VC and reduced PS* scenario)

After the interruption period, the general features of model outputs are

- More active and passive cases and DALYs for a few years compared to the baseline
- New infections decline rapidly in 1–2 years in the *No AS or VC and reduced PS* scenario in health zones with planned VC
- Achieving EoT (i.e. PEoT = 1) with a maximum delay of the length of interruption if it hasn't reach EoT prior to the COVID-19 pandemic

The Programme National de Lutte contre la Trypanosomiase Humaine Africaine (PNLTHA) in the DRC were not able to operate AS through their mobile screening teams for some months in 2020. The provincial-level VC teams managed to continue the scheduled Tiny Target deployment in five health zones with existing VC (i.e. Bandundu, Kikongo, Kwamouth, Masi Manimba, and Yasa Bonga) in 2020 and 2021 [1] so the actual situation might be between the *No AS* and *No AS and reduced PS* scenarios. But all the planned VC in Bokoro, Bolobo, Bulungu, Kokala, and Mushie were postponed, which means the *No AS or VC, and reduced PS* scenario might be considered closer to the reality in these regions.

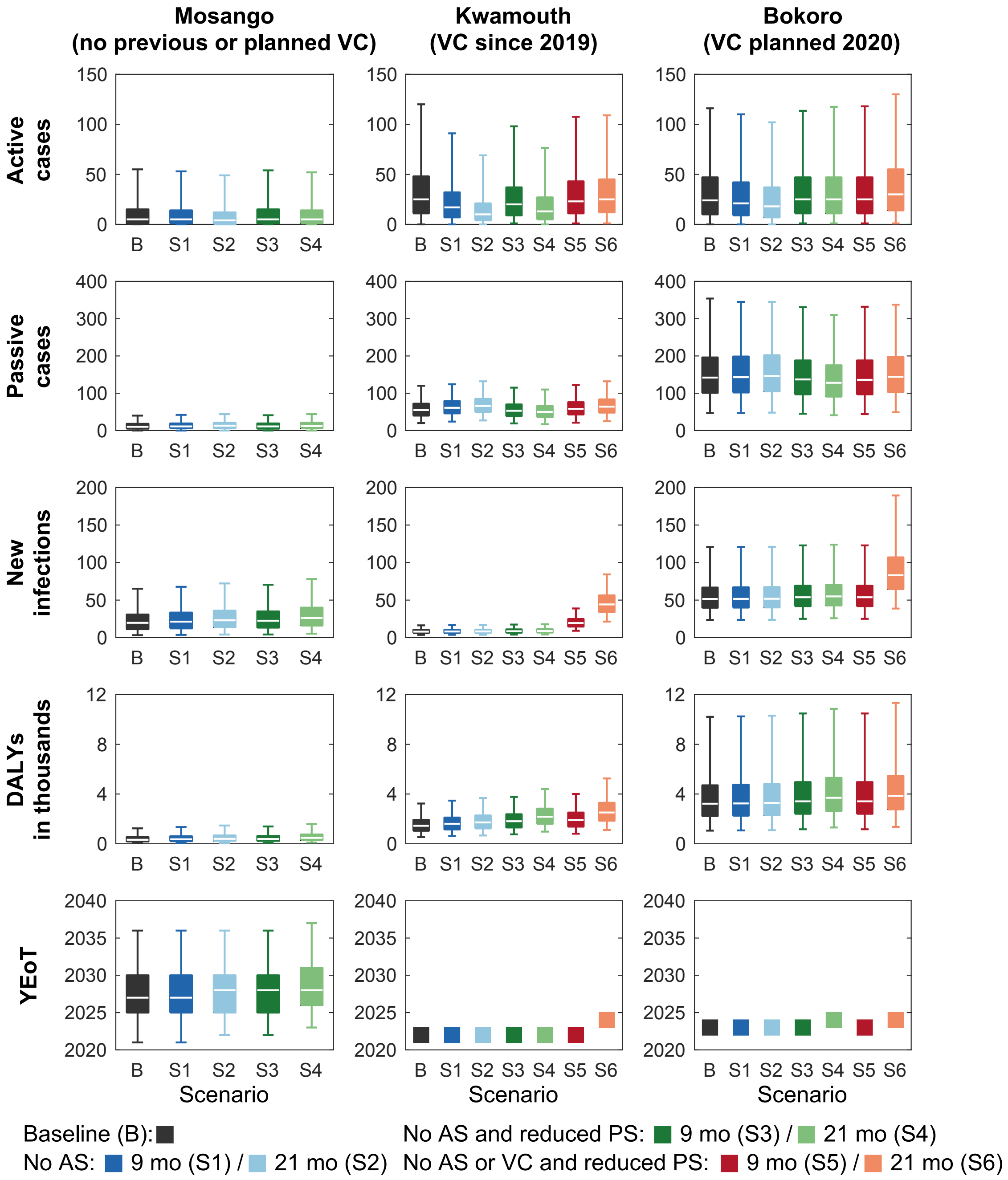

Fig A. Model outputs for 2020–2030 in Mosango, Kwamouth and Bokoro health zones under the baseline and six interruption scenarios. In Mosango only four interruption scenarios are shown as no VC is on-going or planned, therefore S5 and S6 are identical to S3 and S4, respectively. Box and whiskers show medians (centre line), 50% prediction intervals (PIs, box), and 95% PIs (whiskers). Black colours show the baseline (B) scenario, blues show *No AS* scenarios, greens show *No AS and reduced PS* scenarios and reds show *No AS or VC and reduced PS* scenarios. Darker colours show 9-month interruptions and lighter show 21-month interruptions. In the case of the expected year of elimination of transmission (YEoT) in Kwamouth and Bokoro the PIs are present but are very small therefore difficult to visualise.
AS: active screening; PS: passive screening; VC: vector control, DALYs: disability-adjusted life years, YEoT: year of elimination of transmission

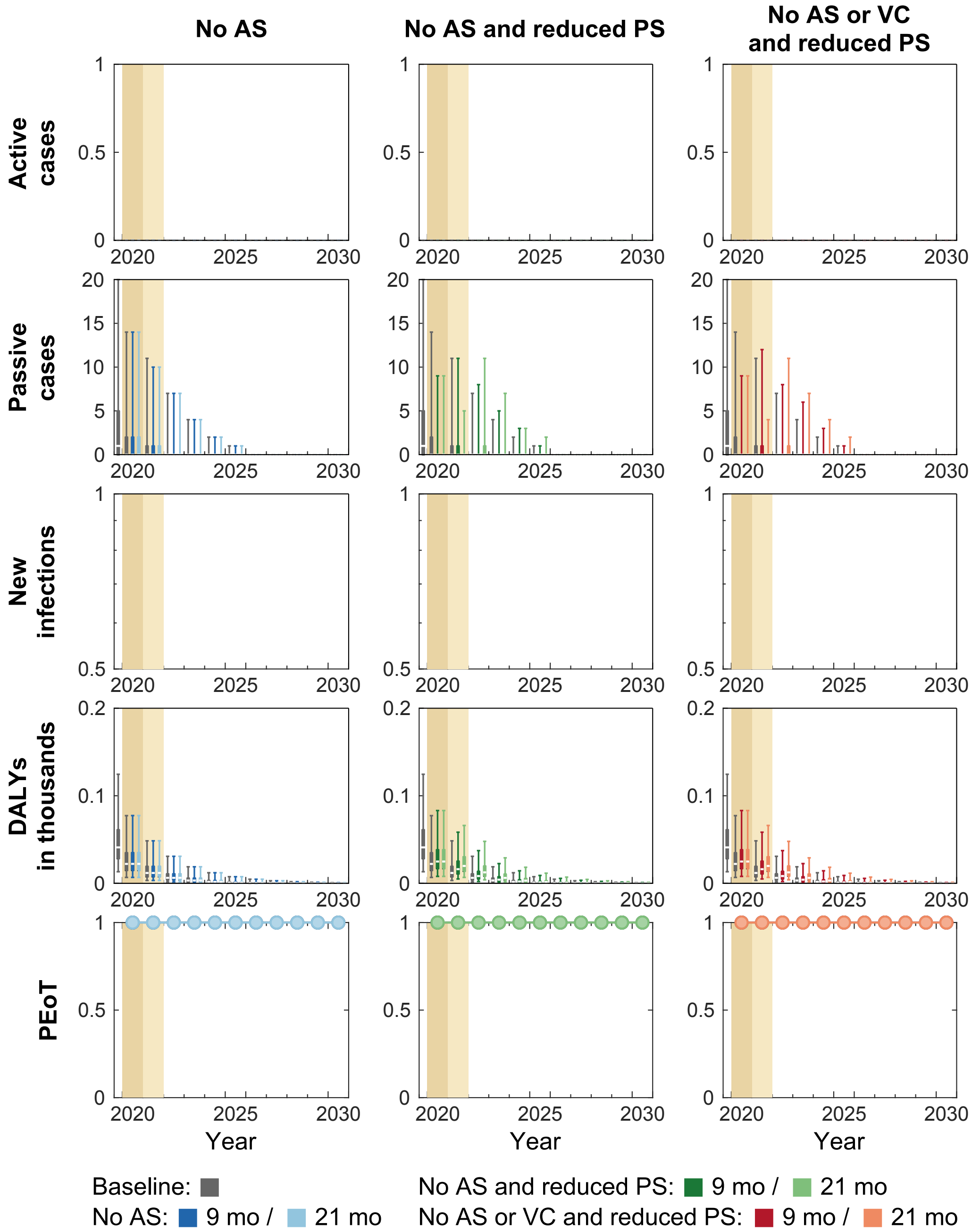

Fig B. Time series of model outputs in Yasa Bonga health zone under the baseline and six interruption scenarios. During 2014–2018, an average of 57% of the population participated in active screening resulting in 2.23 reported cases (from both active and passive screenings) per 10,000 per annum in Yasa Bonga health zone. The first Tiny Target deployment of vector control was in mid-2015. Interruptions by COVID-19 are assumed to take place in April 2020 and last until the end of 2020 or 2021 in our simulations. There are n = 10,000 independent samples, 10 from each of 1,000 independent samples from the joint posterior distributions of the fitted model parameters. Box plots summarise parameter and observational uncertainty. The lines in the boxes represent the medians of predicted results. The lower and upper bounds of the boxes indicate 25th and 75th percentiles. The minimum and maximum values are 2.5th and 97.5th percentiles and therefore whiskers cover 95% prediction intervals. AS: active screening; PS: passive screening; VC: vector control; DALYs: disability-adjusted life years; PEoT: probability of elimination of transmission

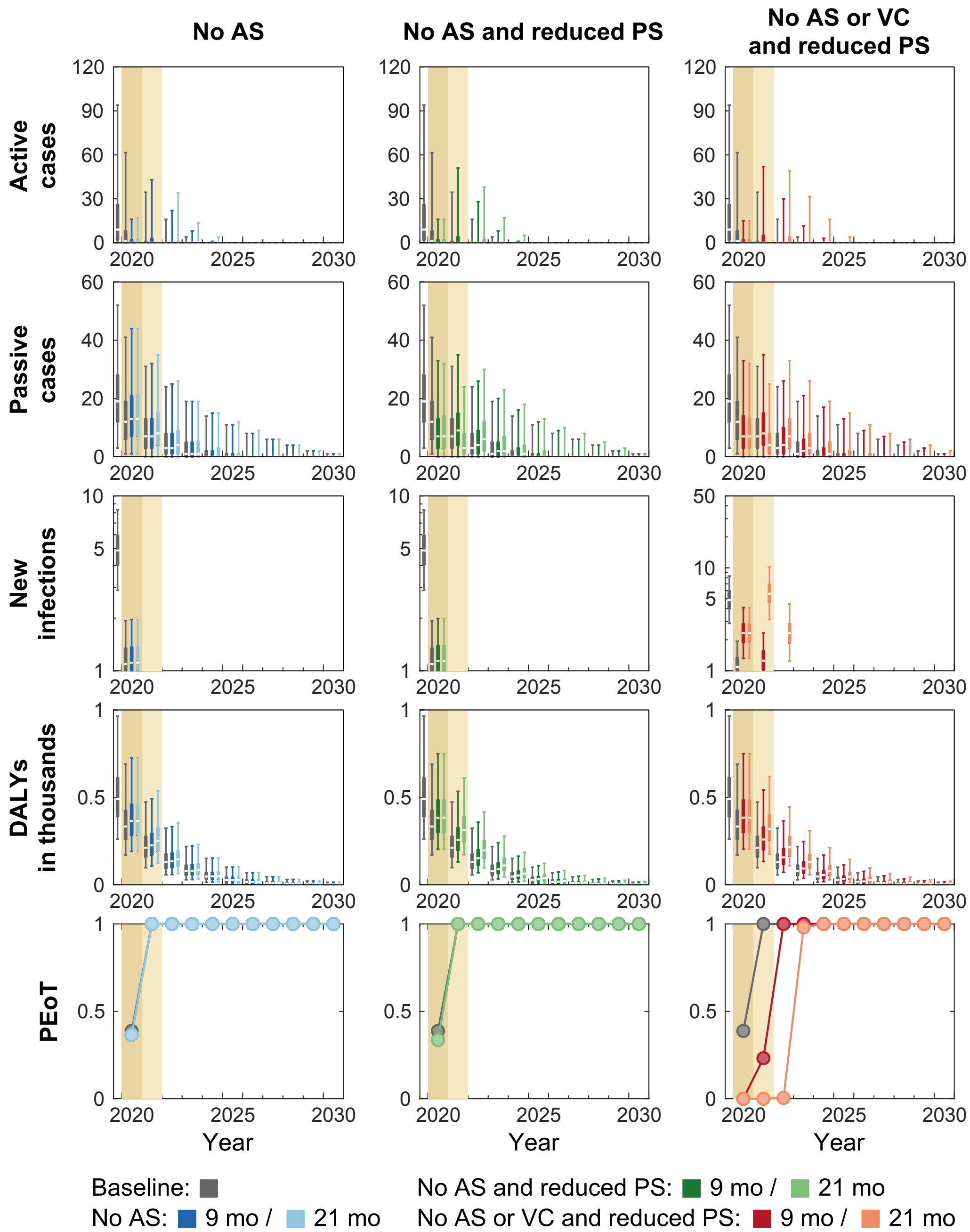

Fig C. Time series of model outputs in Masi Manimba health zone under the baseline and six interruption scenarios. During 2014–2018, an average of 46% of the population participated in active screening resulting in 2.98 reported cases (from both active and passive screenings) per 10,000 per annum in Masi Manimba health zone. The first Tiny Target deployment of vector control was in mid-2018. Interruptions by COVID-19 are assumed to take place in April 2020 and last until the end of 2020 or 2021 in our simulations. There are n = 10,000 independent samples, 10 from each of 1,000 independent samples from the joint posterior distributions of the fitted model parameters. Box plots summarise parameter and observational uncertainty. The lines in the boxes represent the medians of predicted results. The lower and upper bounds of the boxes indicate 25th and 75th percentiles. The minimum and maximum values are 2.5th and 97.5th percentiles and therefore whiskers cover 95% prediction intervals.
AS: active screening; PS: passive screening; VC: vector control; DALYs: disability-adjusted life years; PEoT: probability of elimination of transmission

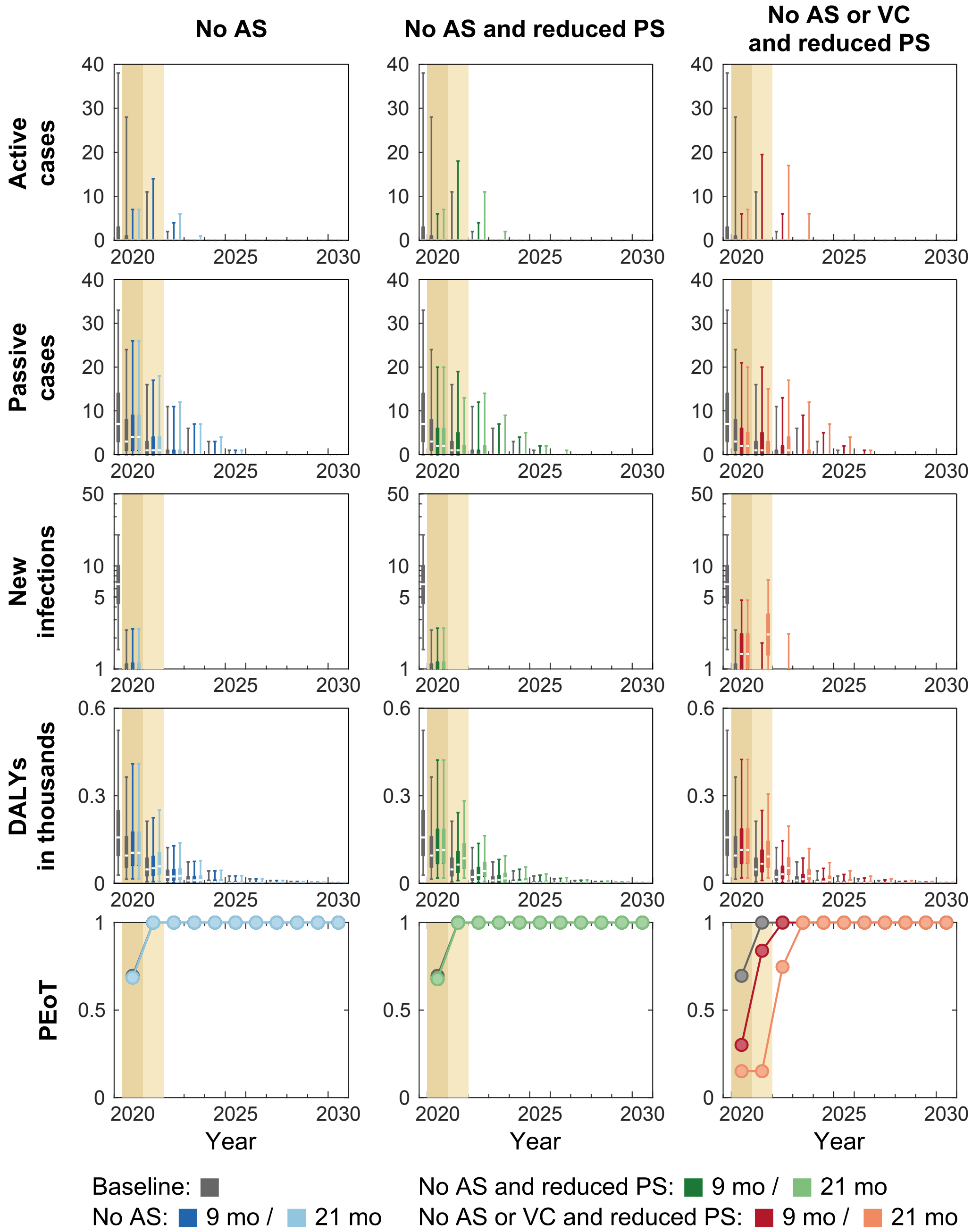

Fig D. Time series of model outputs in Bandundu health zone under the baseline and six interruption scenarios. During 2014–2018, an average of 34% of the population participated in active screening resulting in 2.75 reported cases (from both active and passive screenings) per 10,000 per annum in Bandundu health zone. The first Tiny Target deployment of vector control was in mid-2019. Interruptions by COVID-19 are assumed to take place in April 2020 and last until the end of 2020 or 2021 in our simulations. There are n = 10,000 independent samples, 10 from each of 1,000 independent samples from the joint posterior distributions of the fitted model parameters. Box plots summarise parameter and observational uncertainty. The lines in the boxes represent the medians of predicted results. The lower and upper bounds of the boxes indicate 25th and 75th percentiles. The minimum and maximum values are 2.5th and 97.5th percentiles and therefore whiskers cover 95% prediction intervals. AS: active screening; PS: passive screening; VC: vector control; DALYs: disability-adjusted life years; PEoT: probability of elimination of transmission

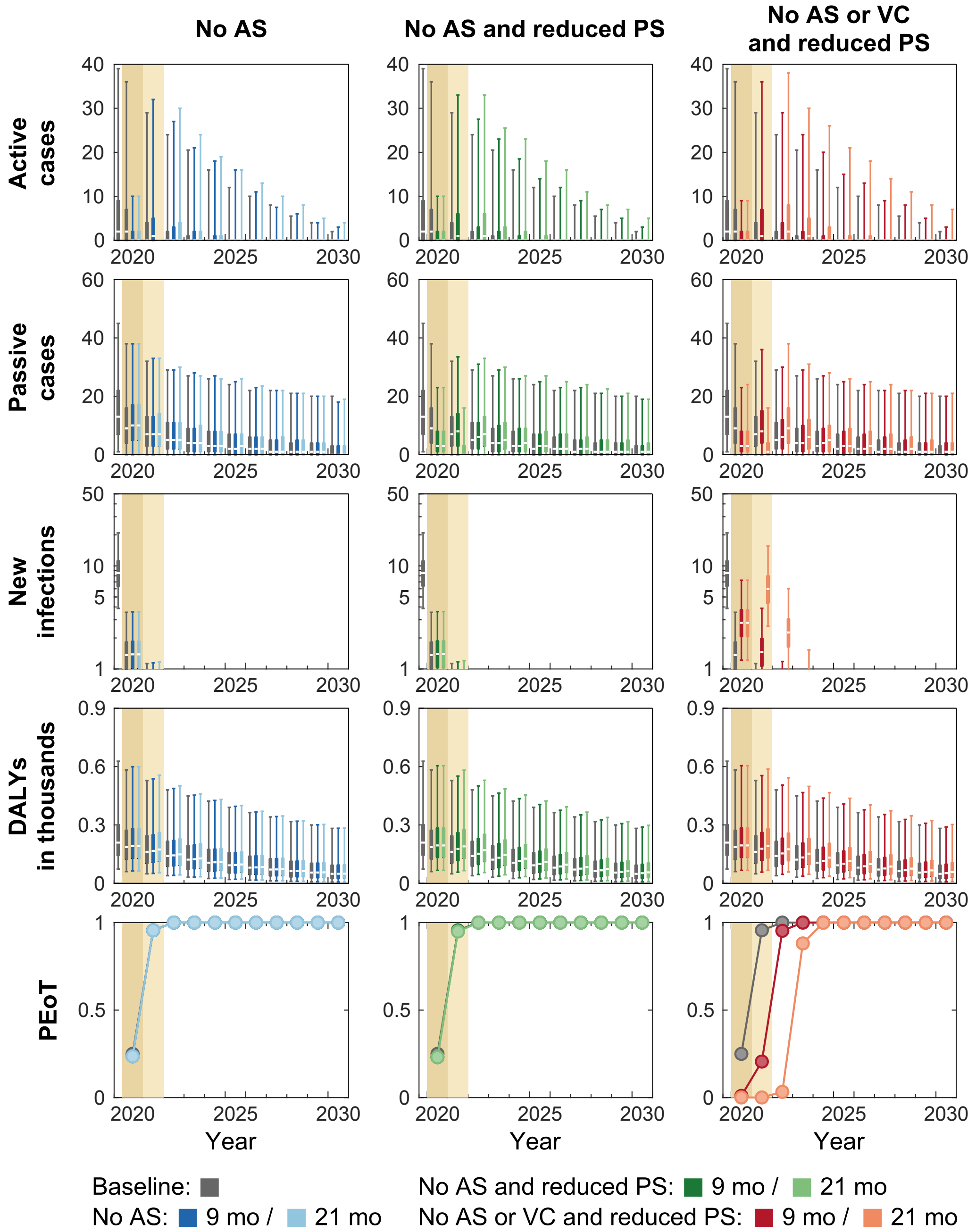

Fig E. Time series of model outputs in Kikongo health zone under the baseline and six interruption scenarios. During 2014–2018, an average of 21% of the population participated in active screening resulting in 2.82 reported cases (from both active and passive screenings) per 10,000 per annum in Kikongo health zone. The first Tiny Target deployment of vector control was in mid-2019. Interruptions by COVID-19 are assumed to take place in April 2020 and last until the end of 2020 or 2021 in our simulations. There are n = 10,000 independent samples, 10 from each of 1,000 independent samples from the joint posterior distributions of the fitted model parameters. Box plots summarise parameter and observational uncertainty. The lines in the boxes represent the medians of predicted results. The lower and upper bounds of the boxes indicate 25th and 75th percentiles. The minimum and maximum values are 2.5th and 97.5th percentiles and therefore whiskers cover 95% prediction intervals. AS: active screening; PS: passive screening; VC: vector control; DALYs: disability-adjusted life years; PEoT: probability of elimination of transmission

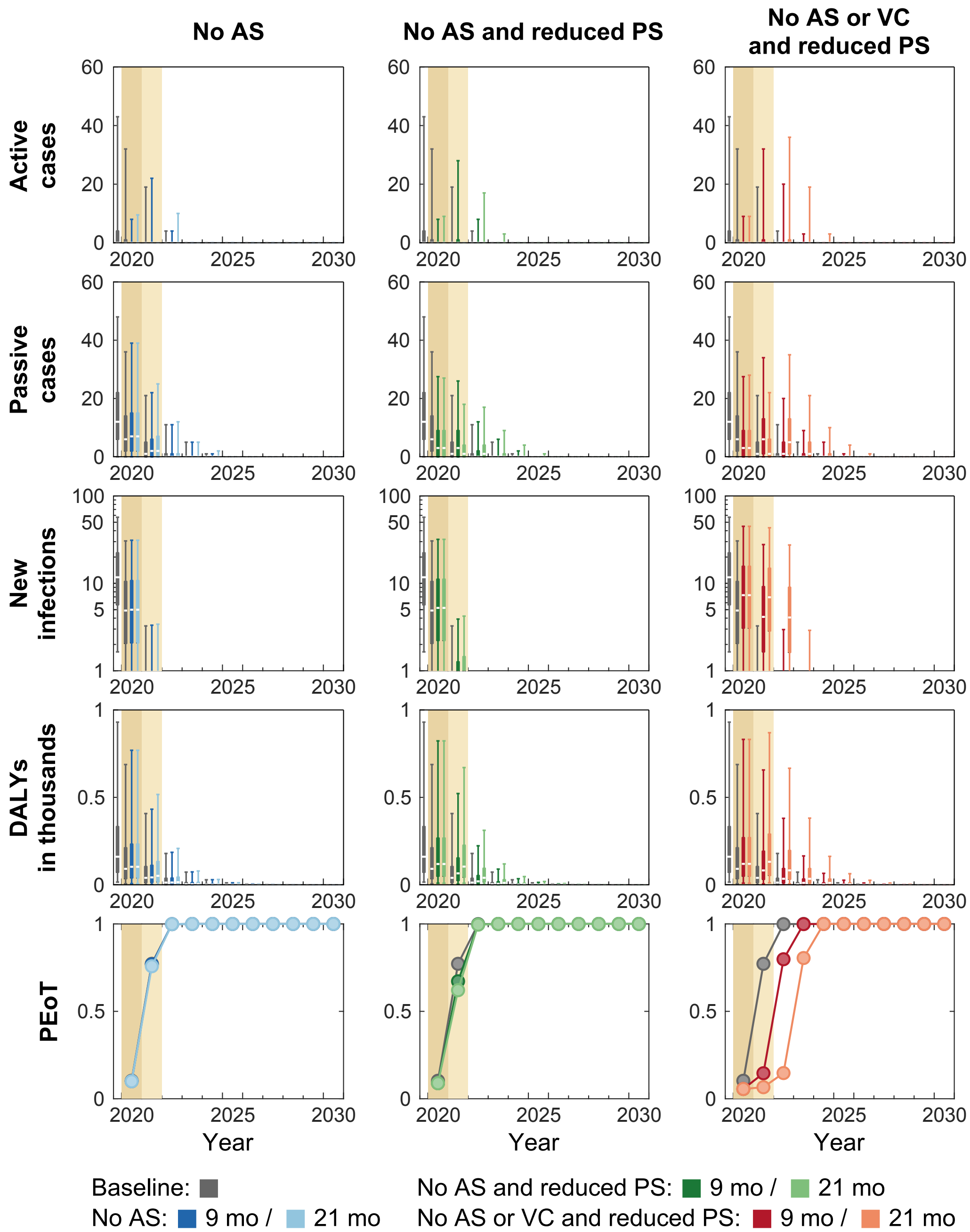

Fig F. Time series of model outputs in Bolobo health zone under the baseline and six interruption scenarios. During 2014–2018, an average of 48% of the population participated in active screening resulting in 9.97 reported cases (from both active and passive screenings) per 10,000 per annum in Bolobo health zone. The first Tiny Target deployment of vector control was scheduled in mid-2020. Interruptions by COVID-19 are assumed to take place in April 2020 and last until the end of 2020 or 2021 in our simulations. There are n = 10,000 independent samples, 10 from each of 1,000 independent samples from the joint posterior distributions of the fitted model parameters. Box plots summarise parameter and observational uncertainty. The lines in the boxes represent the medians of predicted results. The lower and upper bounds of the boxes indicate 25th and 75th percentiles. The minimum and maximum values are 2.5th and 97.5th percentiles and therefore whiskers cover 95% prediction intervals.
AS: active screening; PS: passive screening; VC: vector control; DALYs: disability-adjusted life years; PEoT: probability of elimination of transmission

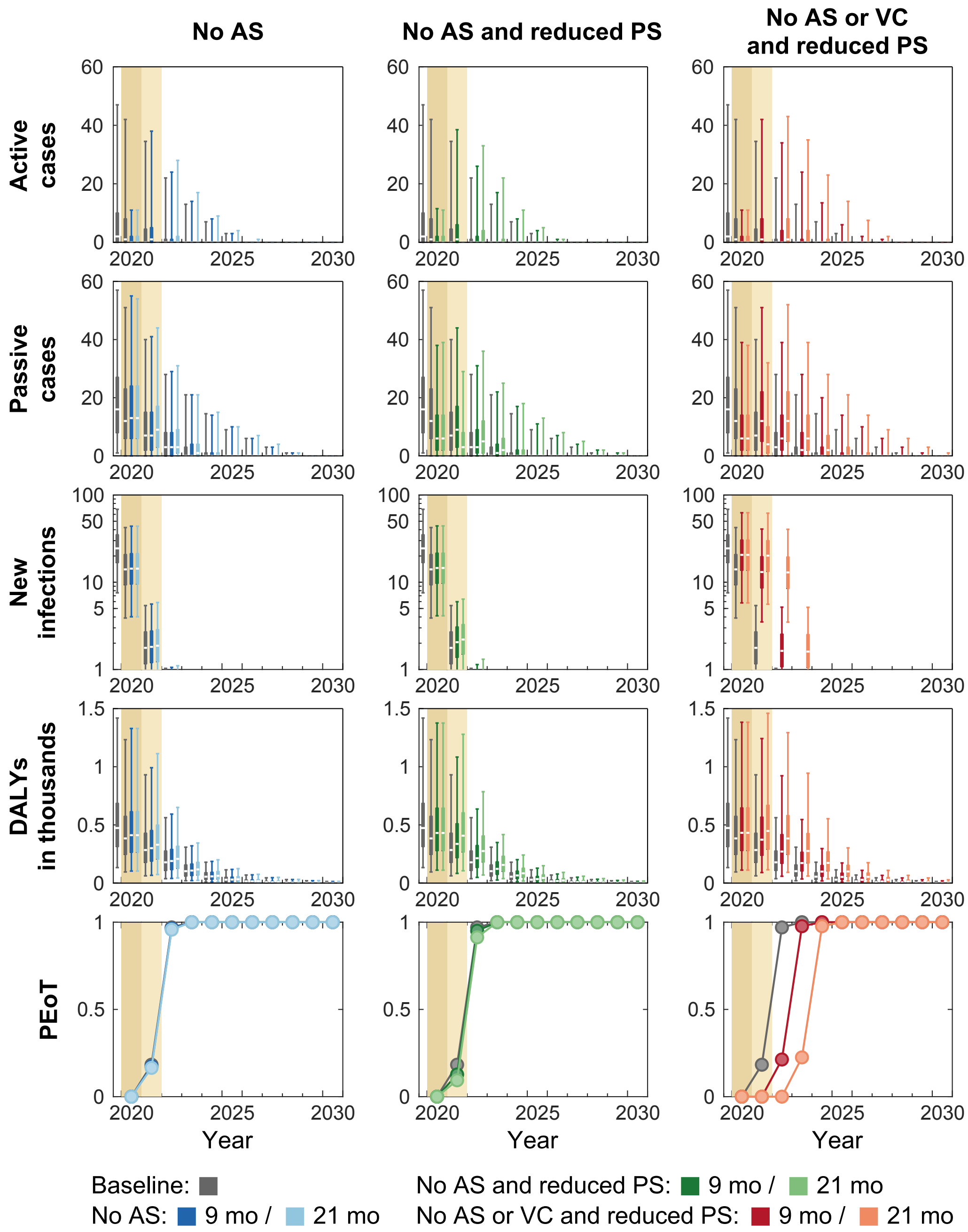

Fig G. Time series of model outputs in Bulungu health zone under the baseline and six interruption scenarios. During 2014–2018, an average of 17% of the population participated in active screening resulting in 2.05 reported cases (from both active and passive screenings) per 10,000 per annum in Bulungu health zone. The first Tiny Target deployment of vector control was scheduled in mid-2020. Interruptions by COVID-19 are assumed to take place in April 2020 and last until the end of 2020 or 2021 in our simulations. There are n = 10,000 independent samples, 10 from each of 1,000 independent samples from the joint posterior distributions of the fitted model parameters. Box plots summarise parameter and observational uncertainty. The lines in the boxes represent the medians of predicted results. The lower and upper bounds of the boxes indicate 25th and 75th percentiles. The minimum and maximum values are 2.5th and 97.5th percentiles and therefore whiskers cover 95% prediction intervals.
AS: active screening; PS: passive screening; VC: vector control; DALYs: disability-adjusted life years; PEoT: probability of elimination of transmission

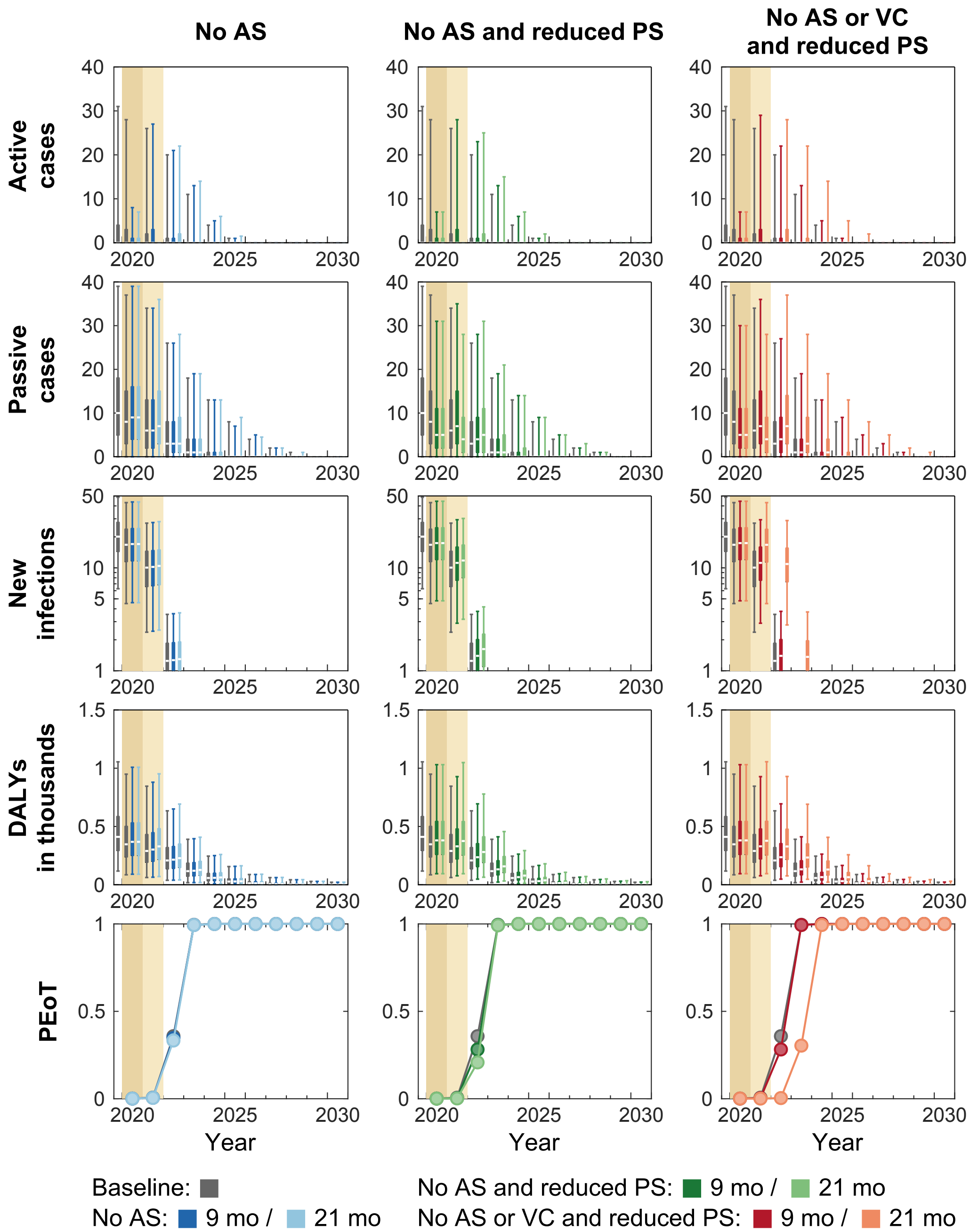

Fig H. Time series of model outputs in Mokala health zone under the baseline and six interruption scenarios. During 2014–2018, an average of 20% of the population participated in active screening resulting in 2.18 reported cases (from both active and passive screenings) per 10,000 per annum in Mokala health zone. The first Tiny Target deployment of vector control was scheduled in mid-2021. Interruptions by COVID-19 are assumed to take place in April 2020 and last until the end of 2020 or 2021 in our simulations. There are n = 10,000 independent samples, 10 from each of 1,000 independent samples from the joint posterior distributions of the fitted model parameters. Box plots summarise parameter and observational uncertainty. The lines in the boxes represent the medians of predicted results. The lower and upper bounds of the boxes indicate 25th and 75th percentiles. The minimum and maximum values are 2.5th and 97.5th percentiles and therefore whiskers cover 95% prediction intervals.
AS: active screening; PS: passive screening; VC: vector control; DALYs: disability-adjusted life years; PEoT: probability of elimination of transmission

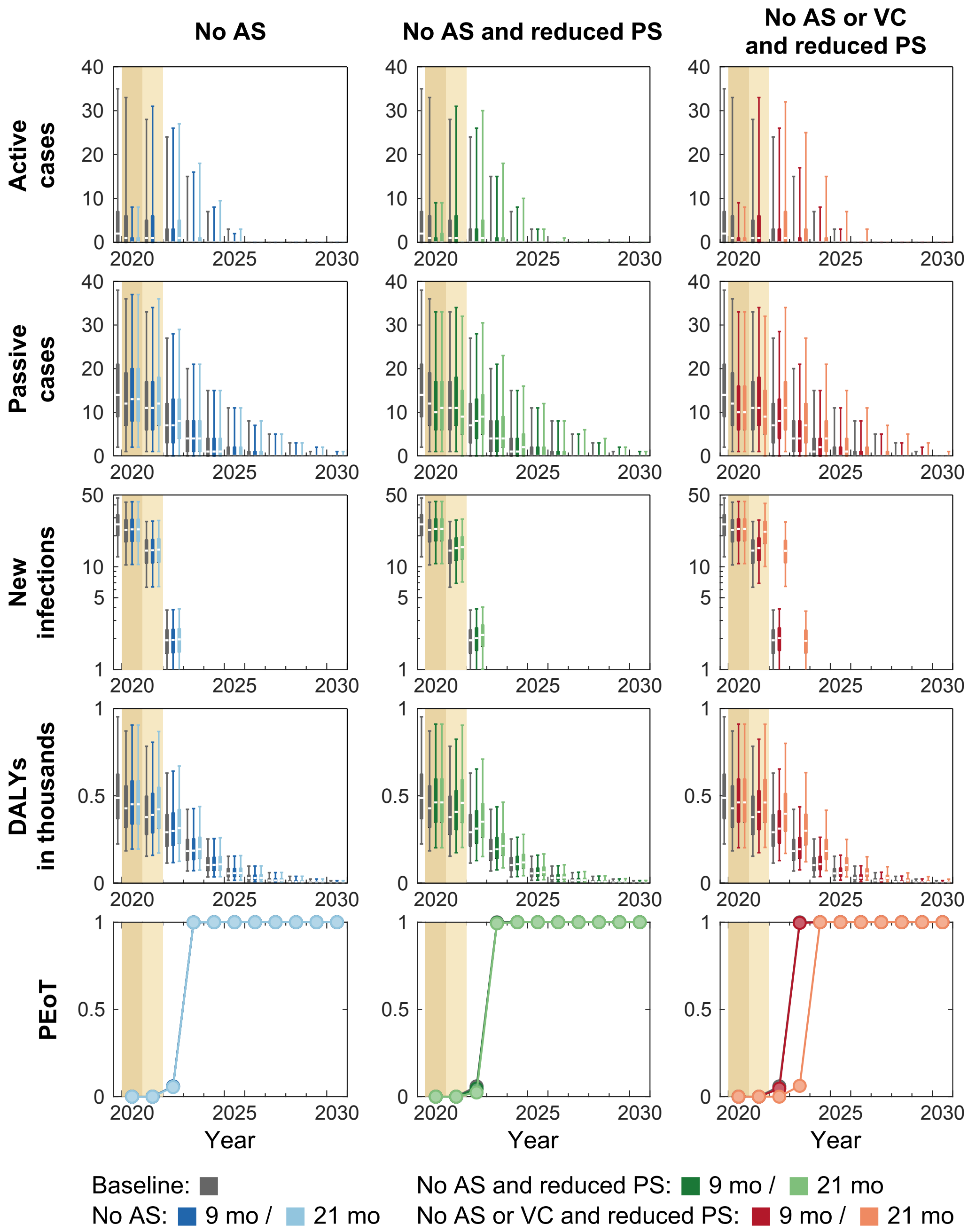

Fig I. Time series of model outputs in Mushie health zone under the baseline and six interruption scenarios. During 2014–2018, an average of 29% of the population participated in active screening resulting in 3.83 reported cases (from both active and passive screenings) per 10,000 per annum in Mushie health zone. The first Tiny Target deployment of vector control was scheduled in mid-2021. Interruptions by COVID-19 are assumed to take place in April 2020 and last until the end of 2020 or 2021 in our simulations. There are n = 10,000 independent samples, 10 from each of 1,000 independent samples from the joint posterior distributions of the fitted model parameters. Box plots summarise parameter and observational uncertainty. The lines in the boxes represent the medians of predicted results. The lower and upper bounds of the boxes indicate 25th and 75th percentiles. The minimum and maximum values are 2.5th and 97.5th percentiles and therefore whiskers cover 95% prediction intervals.
AS: active screening; PS: passive screening; VC: vector control; DALYs: disability-adjusted life years; PEoT: probability of elimination of transmission

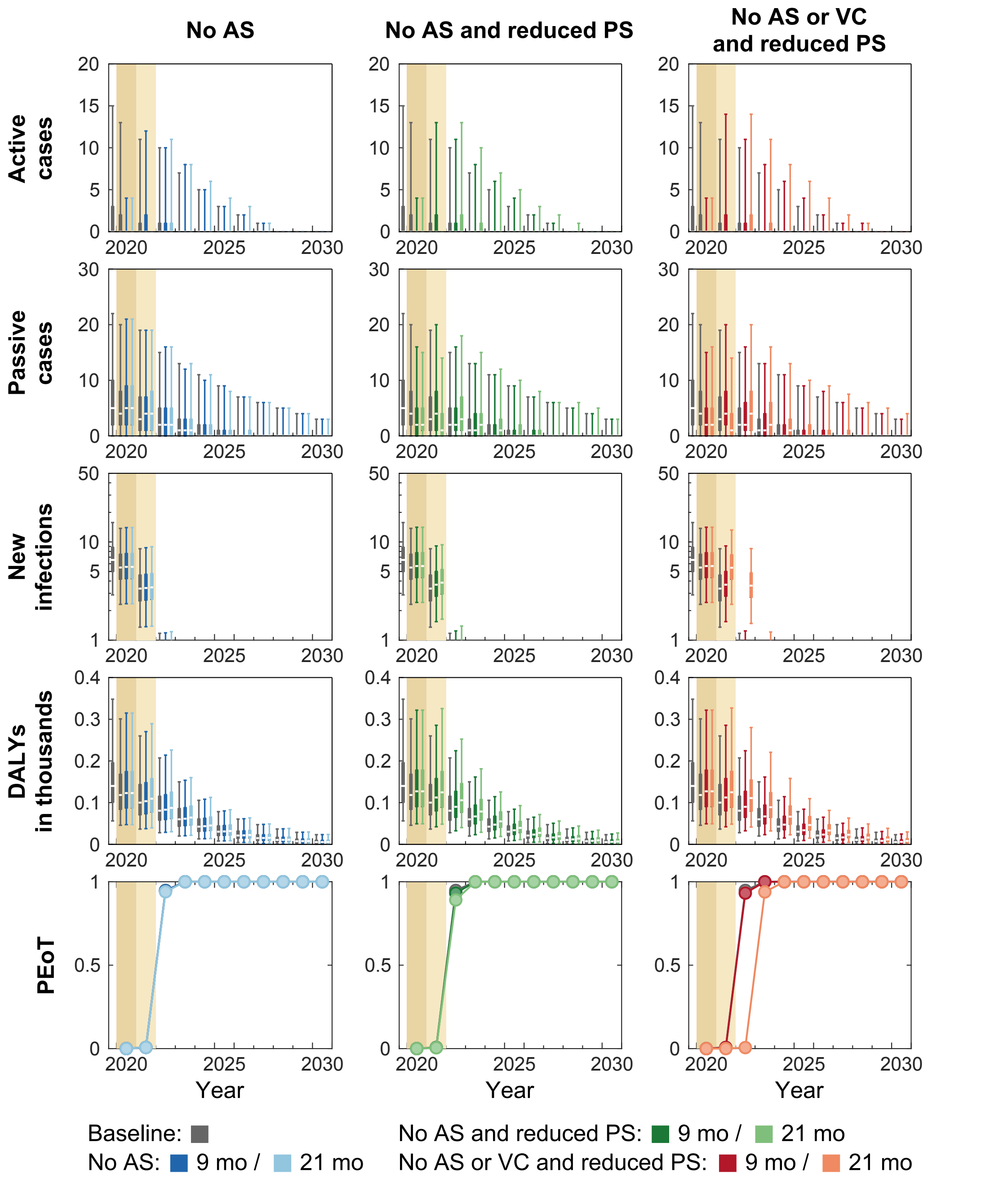

Fig J. Time series of model outputs in Yumbi health zone under the baseline and six interruption scenarios. During 2014–2018, an average of 15% of the population participated in active screening resulting in 1.94 reported cases (from both active and passive screenings) per 10,000 per annum in Yumbi health zone. The first Tiny Target deployment of vector control was scheduled in mid-2021. Interruptions by COVID-19 are assumed to take place in April 2020 and last until the end of 2020 or 2021 in our simulations. There are n = 10,000 independent samples, 10 from each of 1,000 independent samples from the joint posterior distributions of the fitted model parameters. Box plots summarise parameter and observational uncertainty. The lines in the boxes represent the medians of predicted results. The lower and upper bounds of the boxes indicate 25th and 75th percentiles. The minimum and maximum values are 2.5th and 97.5th percentiles and therefore whiskers cover 95% prediction intervals.
AS: active screening; PS: passive screening; VC: vector control; DALYs: disability-adjusted life years; PEoT: probability of elimination of transmission

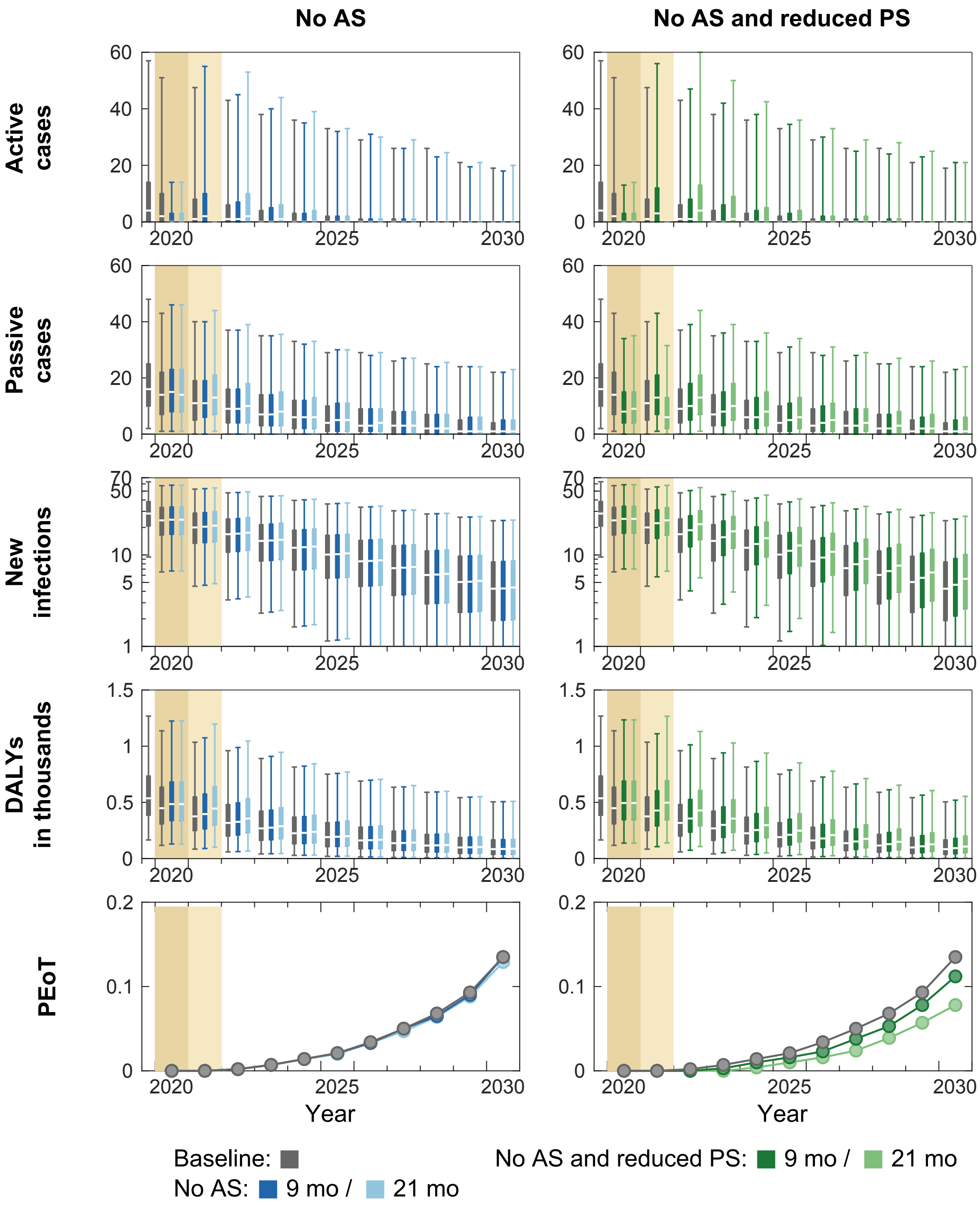

Fig K. Time series of model outputs in Bagata health zone under the baseline and six interruption scenarios. During 2014–2018, an average of 35% of the population participated in active screening resulting in 3.28 reported cases (from both active and passive screenings) per 10,000 per annum in Bagata health zone. There are n = 10,000 independent samples, 10 from each of 1,000 independent samples from the joint posterior distributions of the fitted model parameters. Box plots summarise parameter and observational uncertainty. The lines in the boxes represent the medians of predicted results. The lower and upper bounds of the boxes indicate 25th and 75th percentiles. The minimum and maximum values are 2.5th and 97.5th percentiles and therefore whiskers cover 95% prediction intervals.
AS: active screening; PS: passive screening; VC: vector control; DALYs: disability-adjusted life years; PEoT: probability of elimination of transmission

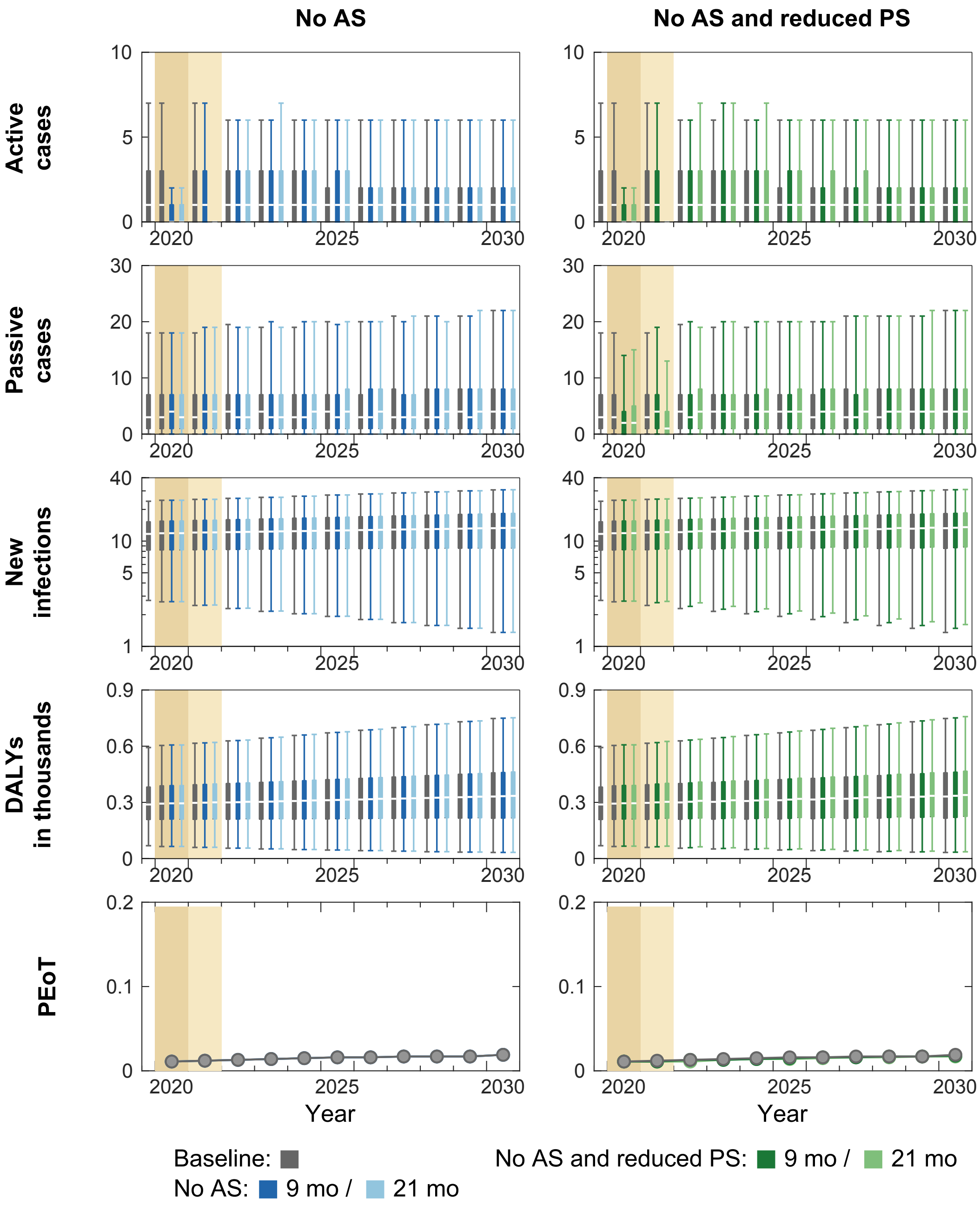

Fig L. Time series of model outputs in Bandjau health zone under the baseline and six interruption scenarios. During 2014–2018, an average of 1% of the population participated in active screening resulting in 0.43 reported cases (from both active and passive screenings) per 10,000 per annum in Bandjau health zone. There are n = 10,000 independent samples, 10 from each of 1,000 independent samples from the joint posterior distributions of the fitted model parameters. Box plots summarise parameter and observational uncertainty. The lines in the boxes represent the medians of predicted results. The lower and upper bounds of the boxes indicate 25th and 75th percentiles. The minimum and maximum values are 2.5th and 97.5th percentiles and therefore whiskers cover 95% prediction intervals.
AS: active screening; PS: passive screening; VC: vector control; DALYs: disability-adjusted life years; PEoT: probability of elimination of transmission

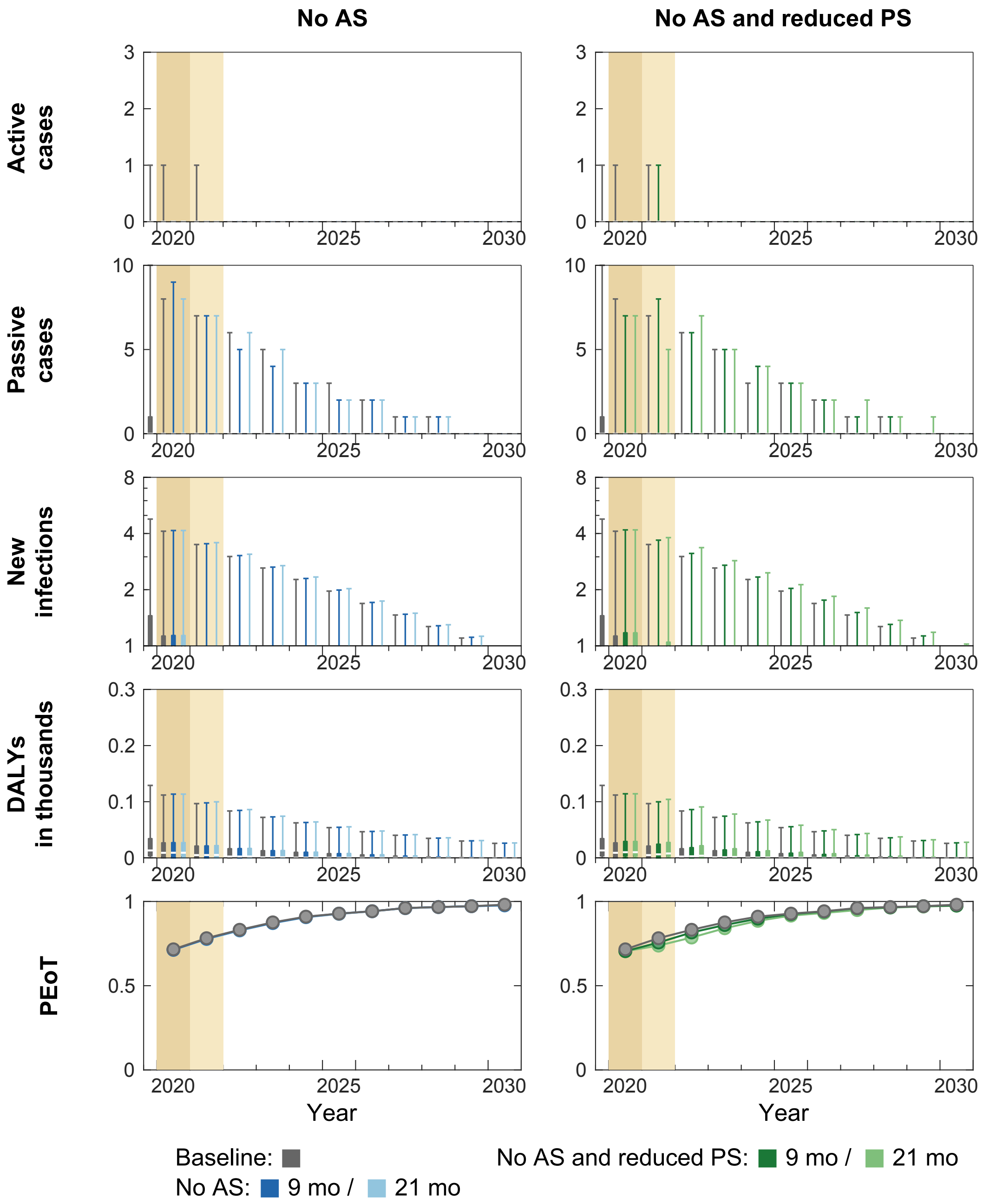

Fig M. Time series of model outputs in Boko health zone under the baseline and six interruption scenarios. During 2014–2018, an average of 3% of the population participated in active screening resulting in 0.14 reported cases (from both active and passive screenings) per 10,000 per annum in Boko health zone. There are n = 10,000 independent samples, 10 from each of 1,000 independent samples from the joint posterior distributions of the fitted model parameters. Box plots summarise parameter and observational uncertainty. The lines in the boxes represent the medians of predicted results. The lower and upper bounds of the boxes indicate 25th and 75th percentiles. The minimum and maximum values are 2.5th and 97.5th percentiles and therefore whiskers cover 95% prediction intervals.
AS: active screening; PS: passive screening; VC: vector control; DALYs: disability-adjusted life years; PEoT: probability of elimination of transmission

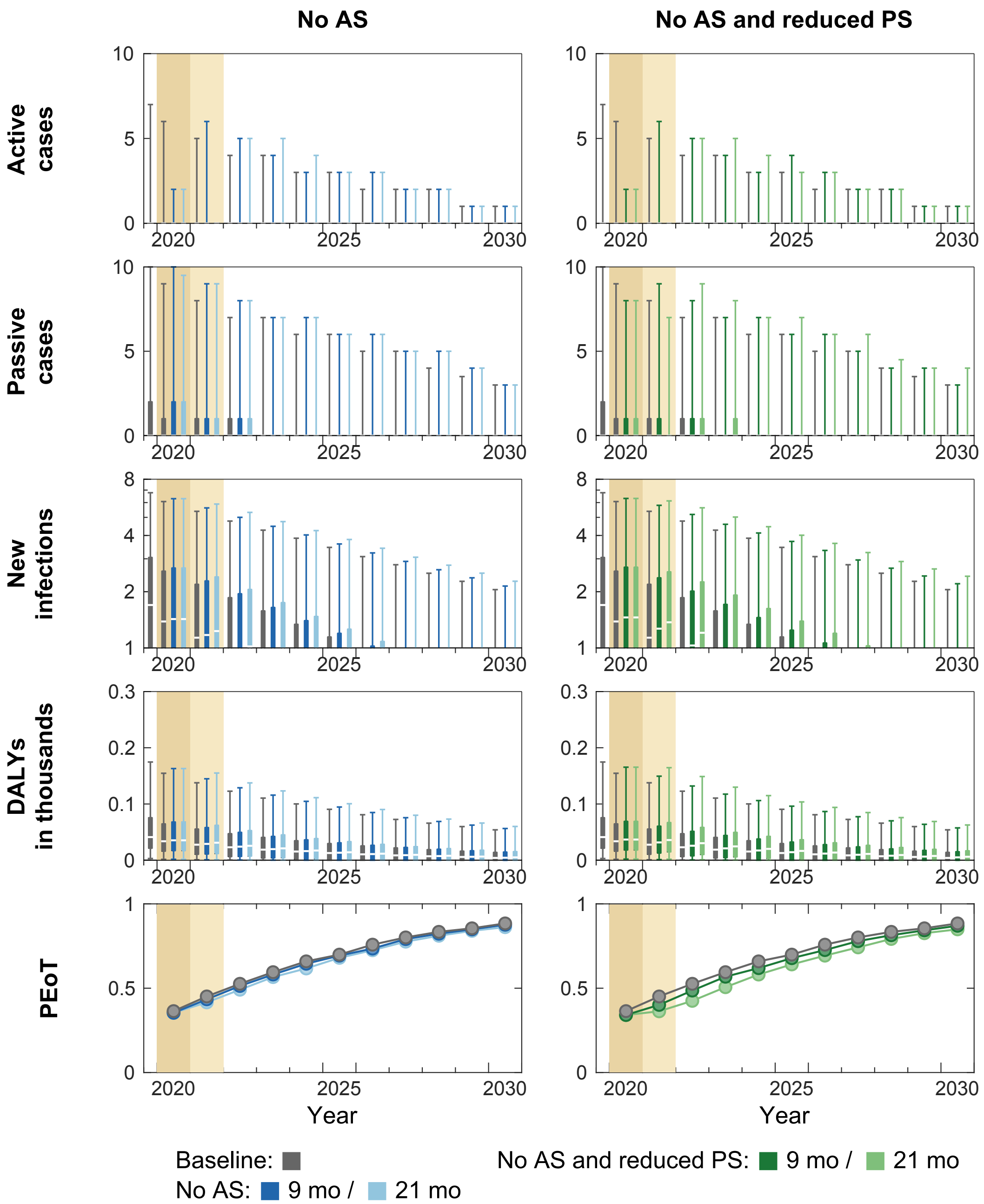

Fig N. Time series of model outputs in Bosobe health zone under the baseline and six interruption scenarios. During 2014–2018, an average of 9% of the population participated in active screening resulting in 0.4 reported cases (from both active and passive screenings) per 10,000 per annum in Bosobe health zone. There are n = 10,000 independent samples, 10 from each of 1,000 independent samples from the joint posterior distributions of the fitted model parameters. Box plots summarise parameter and observational uncertainty. The lines in the boxes represent the medians of predicted results. The lower and upper bounds of the boxes indicate 25th and 75th percentiles. The minimum and maximum values are 2.5th and 97.5th percentiles and therefore whiskers cover 95% prediction intervals. AS: active screening; PS: passive screening; VC: vector control; DALYs: disability-adjusted life years; PEoT: probability of elimination of transmission

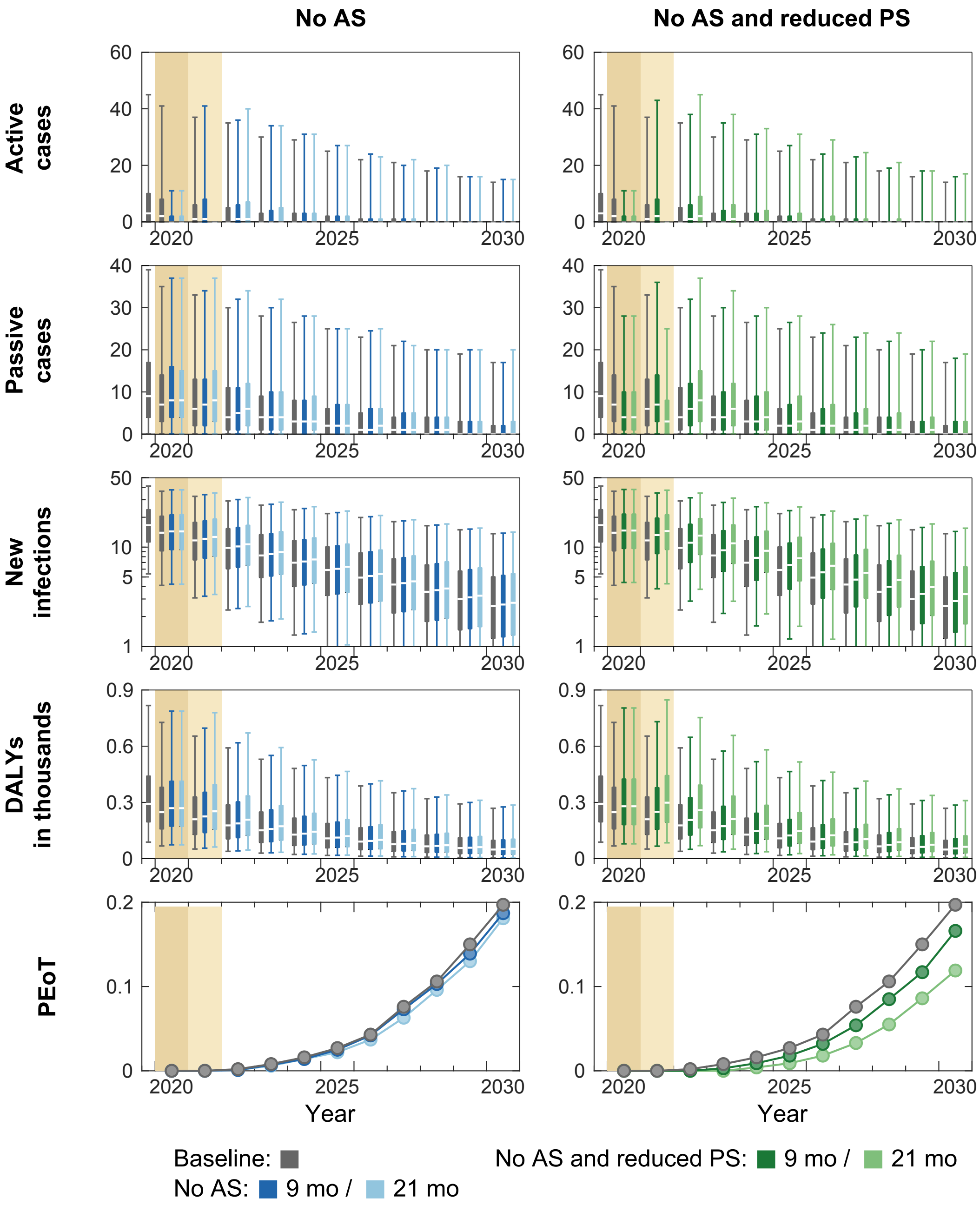

Fig O. Time series of model outputs in Djuma health zone under the baseline and six interruption scenarios. During 2014–2018, an average of 21% of the population participated in active screening resulting in 0.96 reported cases (from both active and passive screenings) per 10,000 per annum in Djuma health zone. There are n = 10,000 independent samples, 10 from each of 1,000 independent samples from the joint posterior distributions of the fitted model parameters. Box plots summarise parameter and observational uncertainty. The lines in the boxes represent the medians of predicted results. The lower and upper bounds of the boxes indicate 25th and 75th percentiles. The minimum and maximum values are 2.5th and 97.5th percentiles and therefore whiskers cover 95% prediction intervals.

AS: active screening; PS: passive screening; VC: vector control; DALYs: disability-adjusted life years; PEoT: probability of elimination of transmission

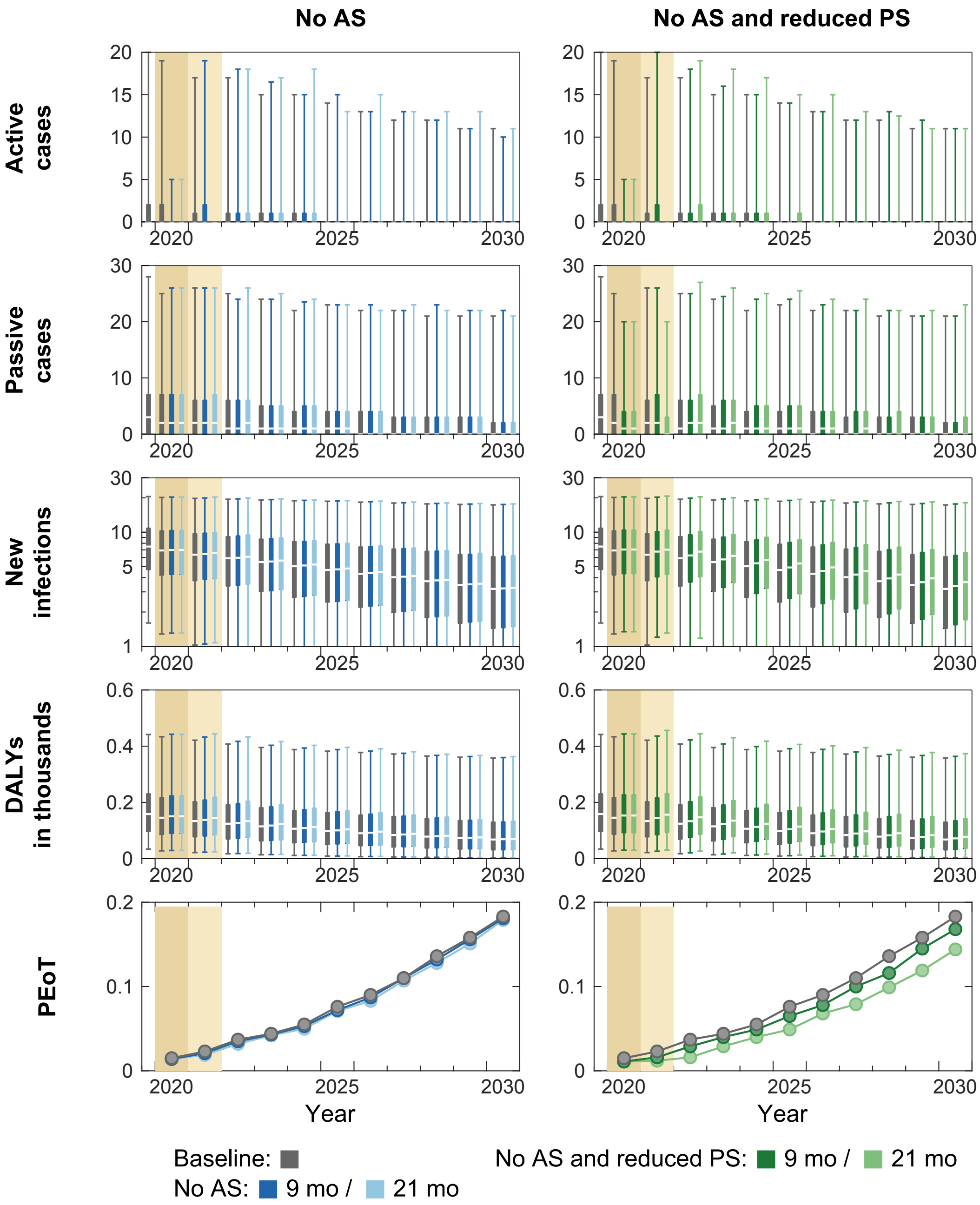

Fig P. Time series of model outputs in Idiofa health zone under the baseline and six interruption scenarios. During 2014–2018, an average of 11% of the population participated in active screening resulting in 0.99 reported cases (from both active and passive screenings) per 10,000 per annum in Idiofa health zone. There are n = 10,000 independent samples, 10 from each of 1,000 independent samples from the joint posterior distributions of the fitted model parameters. Box plots summarise parameter and observational uncertainty. The lines in the boxes represent the medians of predicted results. The lower and upper bounds of the boxes indicate 25th and 75th percentiles. The minimum and maximum values are 2.5th and 97.5th percentiles and therefore whiskers cover 95% prediction intervals.

AS: active screening; PS: passive screening; VC: vector control; DALYs: disability-adjusted life years; PEoT: probability of elimination of transmission

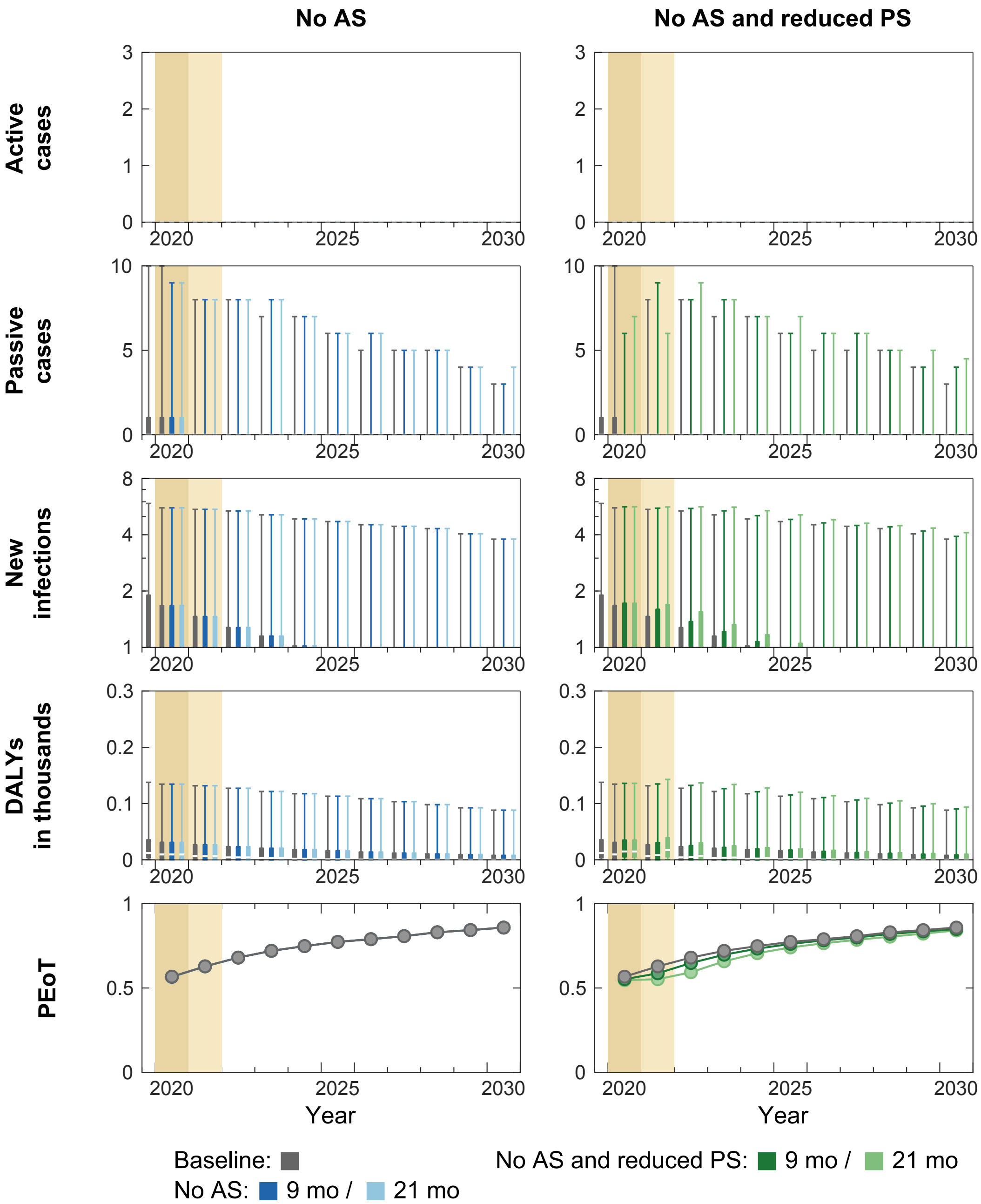

Fig Q. Time series of model outputs in Inongo health zone under the baseline and six interruption scenarios. During 2014–2018, an average of 0% of the population participated in active screening resulting in 0.08 reported cases (from both active and passive screenings) per 10,000 per annum in Inongo health zone. There are n = 10,000 independent samples, 10 from each of 1,000 independent samples from the joint posterior distributions of the fitted model parameters. Box plots summarise parameter and observational uncertainty. The lines in the boxes represent the medians of predicted results. The lower and upper bounds of the boxes indicate 25th and 75th percentiles. The minimum and maximum values are 2.5th and 97.5th percentiles and therefore whiskers cover 95% prediction intervals.
AS: active screening; PS: passive screening; VC: vector control; DALYs: disability-adjusted life years; PEoT: probability of elimination of transmission

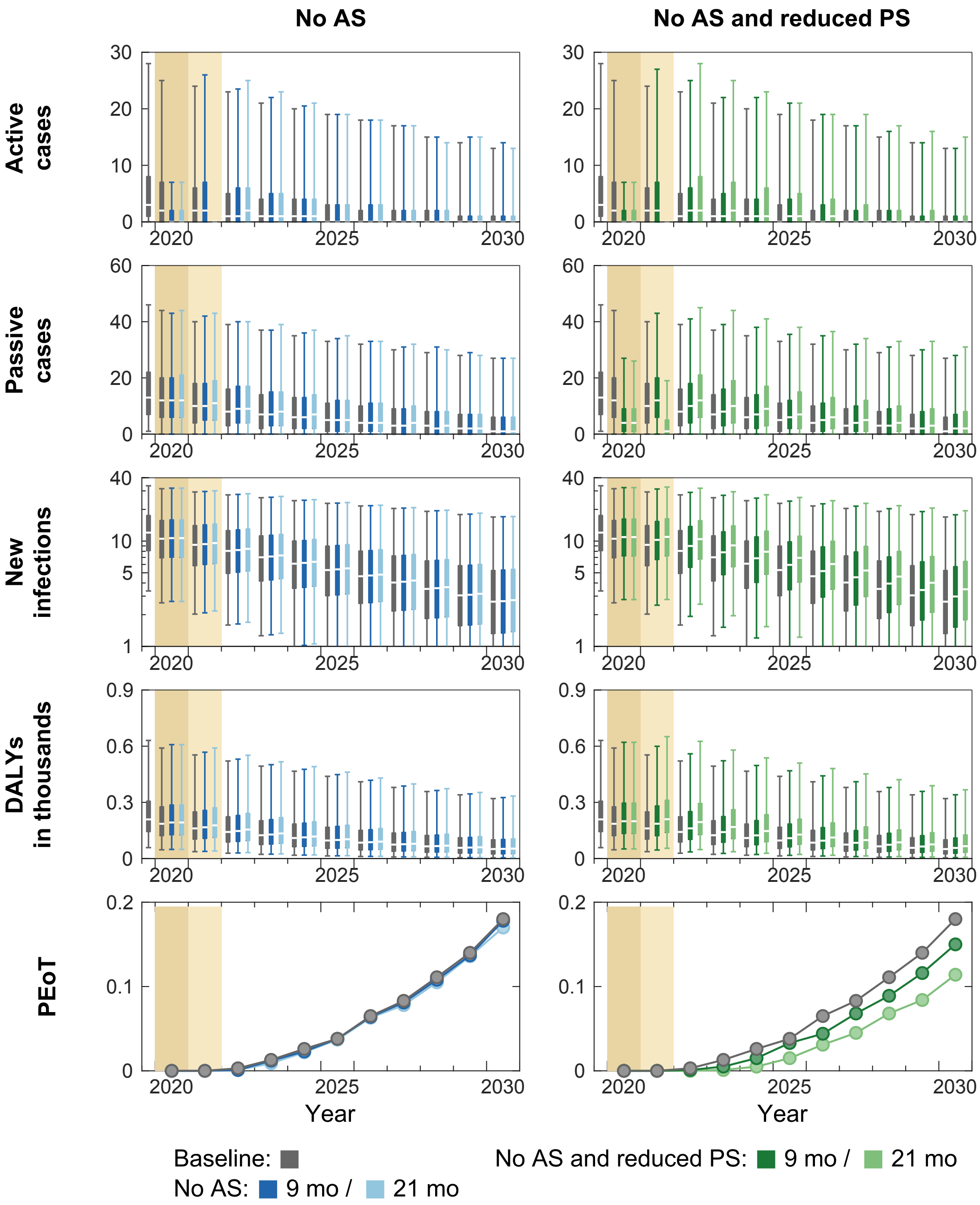

Fig R. Time series of model outputs in Ipamu health zone under the baseline and six interruption scenarios. During 2014–2018, an average of 10% of the population participated in active screening resulting in 1.52 reported cases (from both active and passive screenings) per 10,000 per annum in Ipamu health zone. There are n = 10,000 independent samples, 10 from each of 1,000 independent samples from the joint posterior distributions of the fitted model parameters. Box plots summarise parameter and observational uncertainty. The lines in the boxes represent the medians of predicted results. The lower and upper bounds of the boxes indicate 25th and 75th percentiles. The minimum and maximum values are 2.5th and 97.5th percentiles and therefore whiskers cover 95% prediction intervals.
AS: active screening; PS: passive screening; VC: vector control; DALYs: disability-adjusted life years; PEoT: probability of elimination of transmission

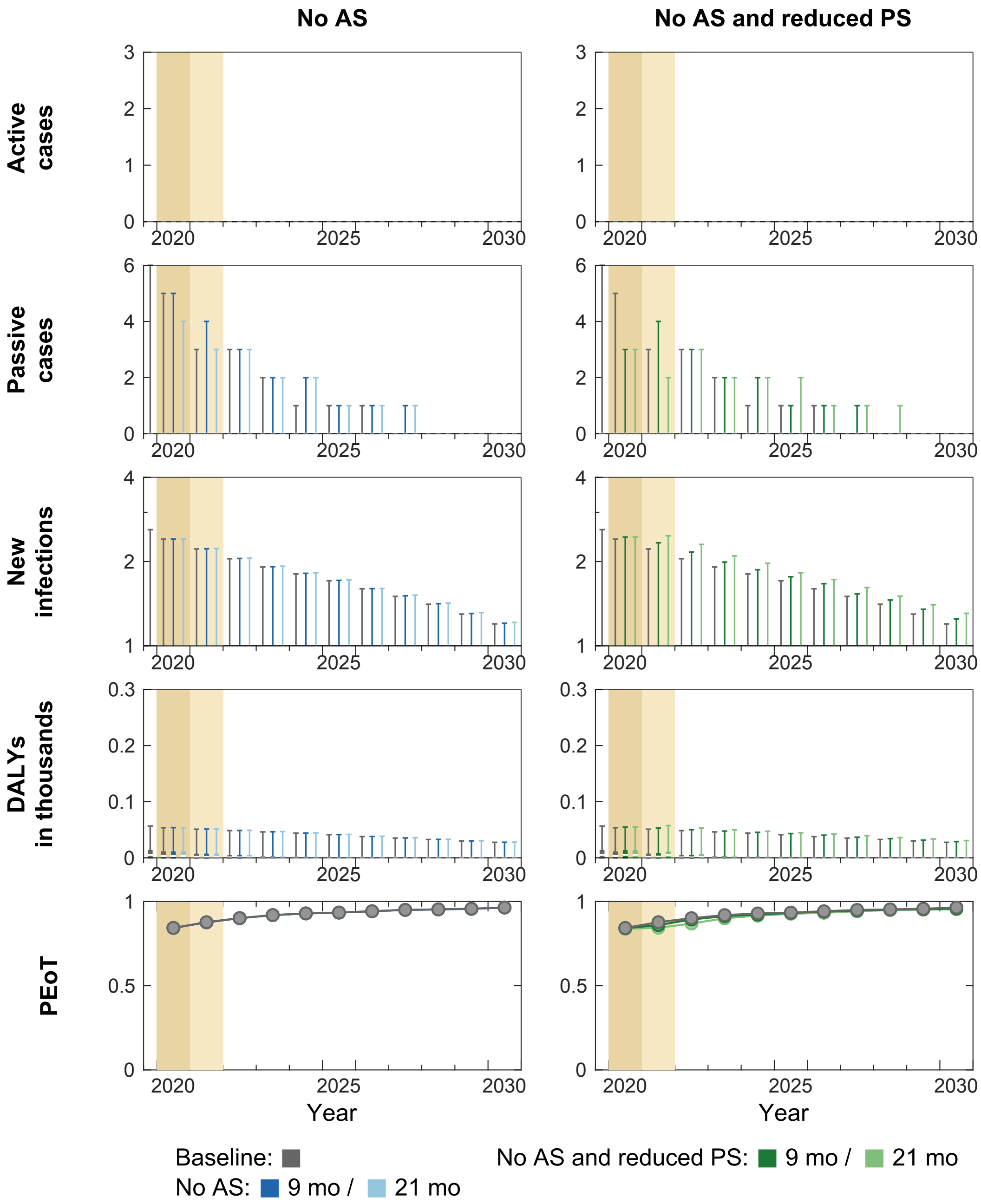

Fig S. Time series of model outputs in Kasongolunda health zone under the baseline and six interruption scenarios. During 2014–2018, an average of 2% of the population participated in active screening resulting in 0.06 reported cases (from both active and passive screenings) per 10,000 per annum in Kasongolunda health zone. There are n = 10,000 independent samples, 10 from each of 1,000 independent samples from the joint posterior distributions of the fitted model parameters. Box plots summarise parameter and observational uncertainty. The lines in the boxes represent the medians of predicted results. The lower and upper bounds of the boxes indicate 25th and 75th percentiles. The minimum and maximum values are 2.5th and 97.5th percentiles and therefore whiskers cover 95% prediction intervals.
AS: active screening; PS: passive screening; VC: vector control; DALYs: disability-adjusted life years; PEoT: probability of elimination of transmission

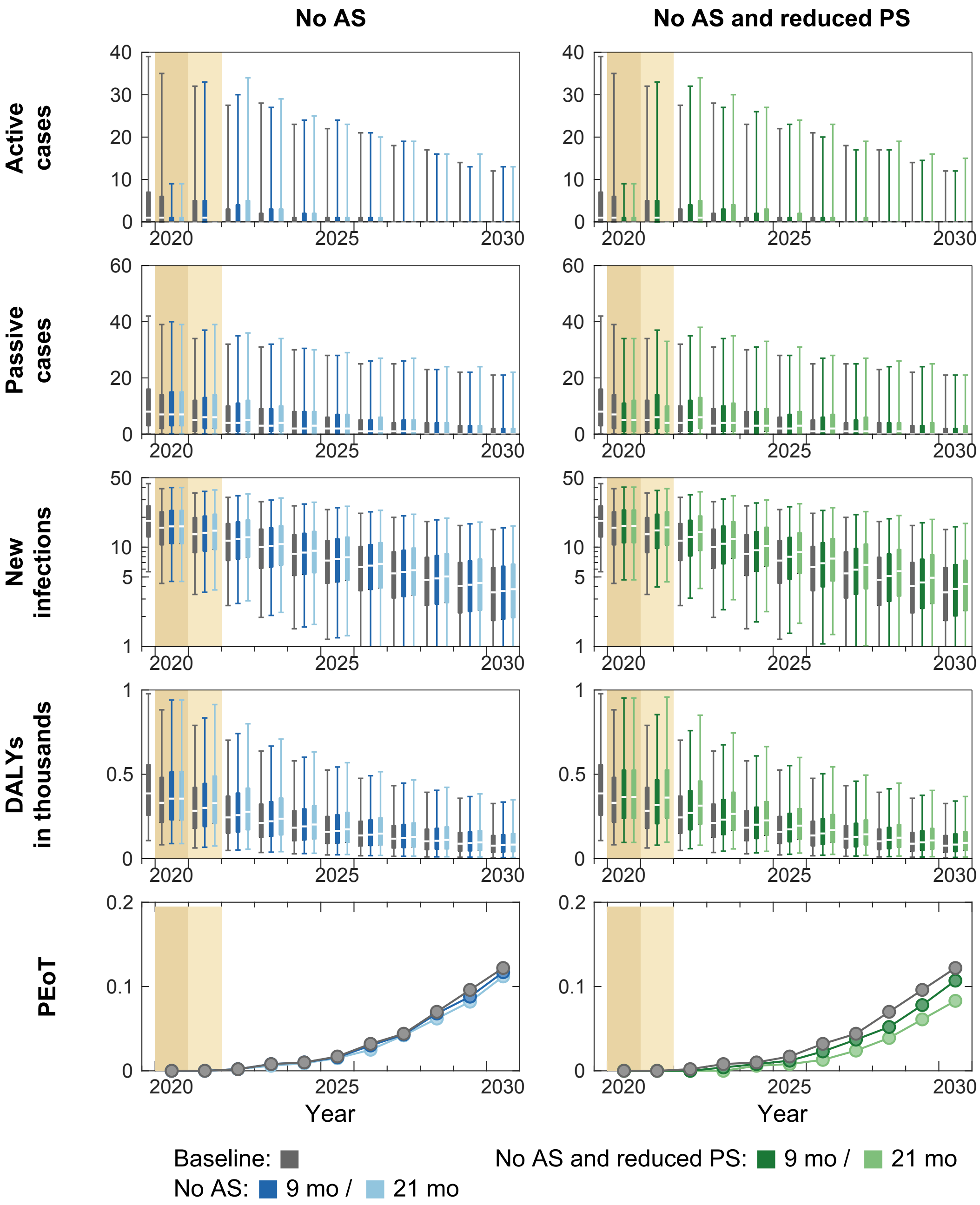

Fig T. Time series of model outputs in Kenge health zone under the baseline and six interruption scenarios. During 2014–2018, an average of 15% of the population participated in active screening resulting in 0.71 reported cases (from both active and passive screenings) per 10,000 per annum in Kenge health zone. There are n = 10,000 independent samples, 10 from each of 1,000 independent samples from the joint posterior distributions of the fitted model parameters. Box plots summarise parameter and observational uncertainty. The lines in the boxes represent the medians of predicted results. The lower and upper bounds of the boxes indicate 25th and 75th percentiles. The minimum and maximum values are 2.5th and 97.5th percentiles and therefore whiskers cover 95% prediction intervals.
AS: active screening; PS: passive screening; VC: vector control; DALYs: disability-adjusted life years; PEoT: probability of elimination of transmission

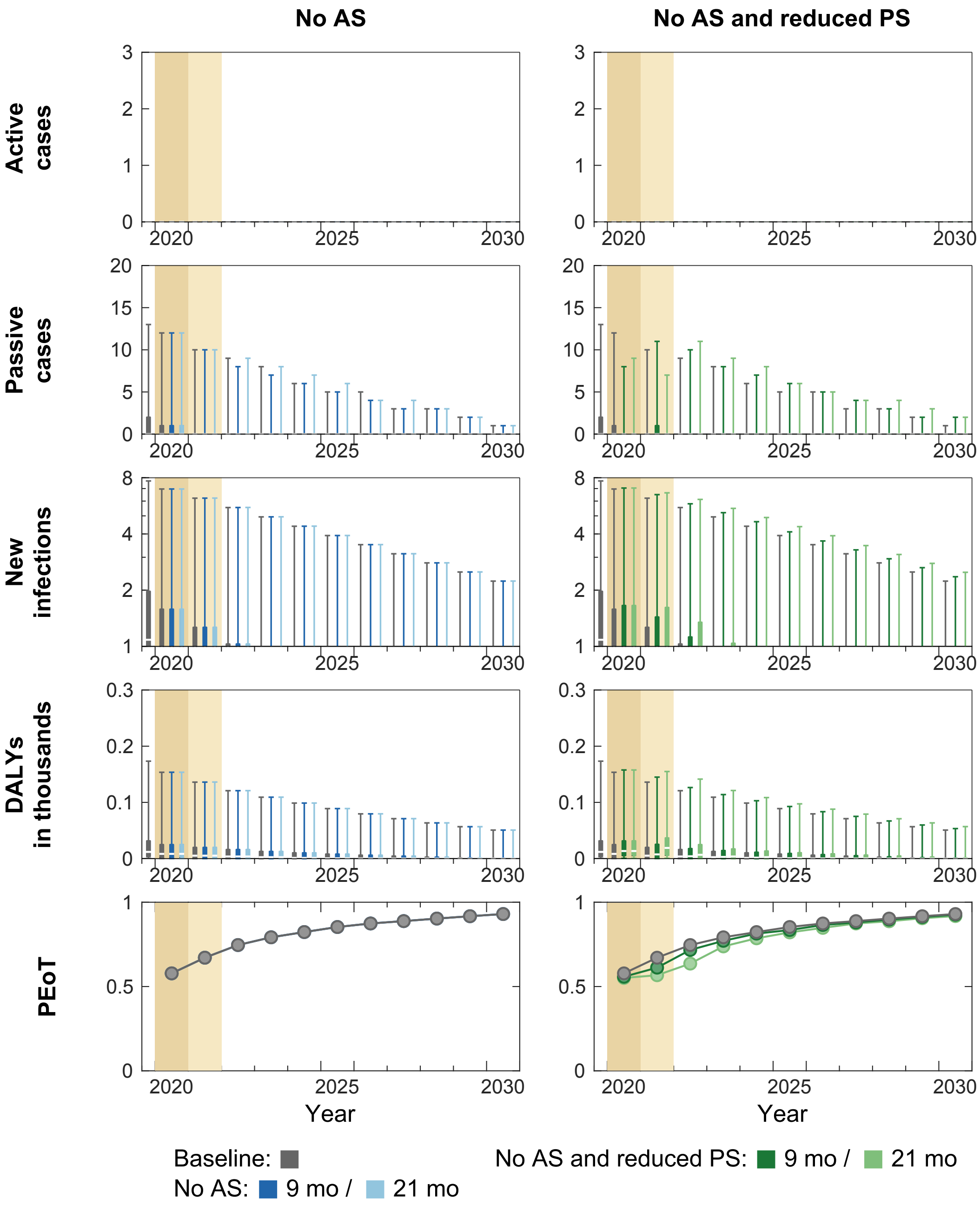

Fig U. Time series of model outputs in Kikwit Nord health zone under the baseline and six interruption scenarios. During 2014–2018, an average of 0% of the population participated in active screening resulting in 0.08 reported cases (from both active and passive screenings) per 10,000 per annum in Kikwit Nord health zone. There are n = 10,000 independent samples, 10 from each of 1,000 independent samples from the joint posterior distributions of the fitted model parameters. Box plots summarise parameter and observational uncertainty. The lines in the boxes represent the medians of predicted results. The lower and upper bounds of the boxes indicate 25th and 75th percentiles. The minimum and maximum values are 2.5th and 97.5th percentiles and therefore whiskers cover 95% prediction intervals.
AS: active screening; PS: passive screening; VC: vector control; DALYs: disability-adjusted life years; PEoT: probability of elimination of transmission

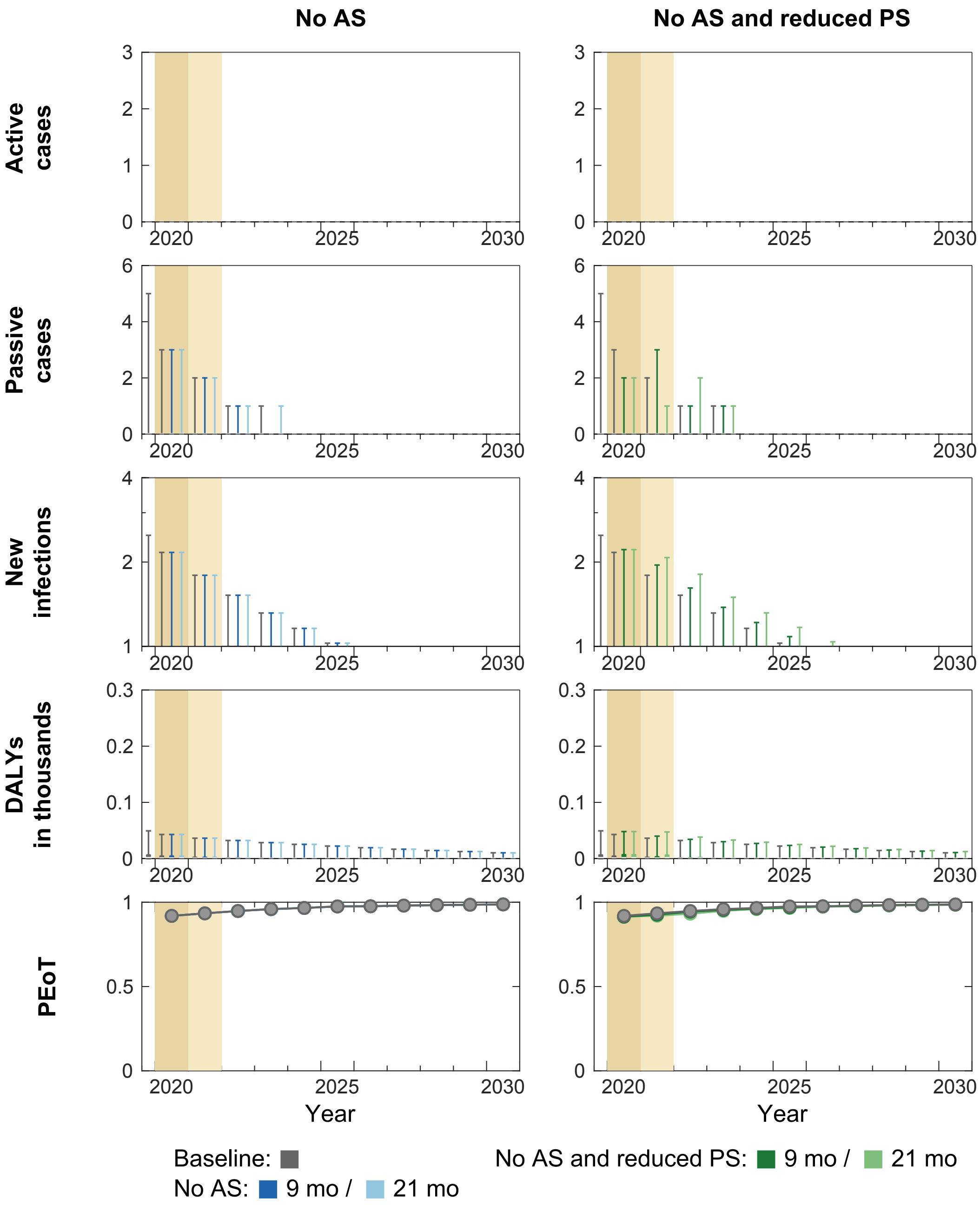

Fig V. Time series of model outputs in Kikwit Sud health zone under the baseline and six interruption scenarios. During 2014–2018, an average of 0% of the population participated in active screening resulting in 0.04 reported cases (from both active and passive screenings) per 10,000 per annum in Kikwit Sud health zone. There are n = 10,000 independent samples, 10 from each of 1,000 independent samples from the joint posterior distributions of the fitted model parameters. Box plots summarise parameter and observational uncertainty. The lines in the boxes represent the medians of predicted results. The lower and upper bounds of the boxes indicate 25th and 75th percentiles. The minimum and maximum values are 2.5th and 97.5th percentiles and therefore whiskers cover 95% prediction intervals.
AS: active screening; PS: passive screening; VC: vector control; DALYs: disability-adjusted life years; PEoT: probability of elimination of transmission

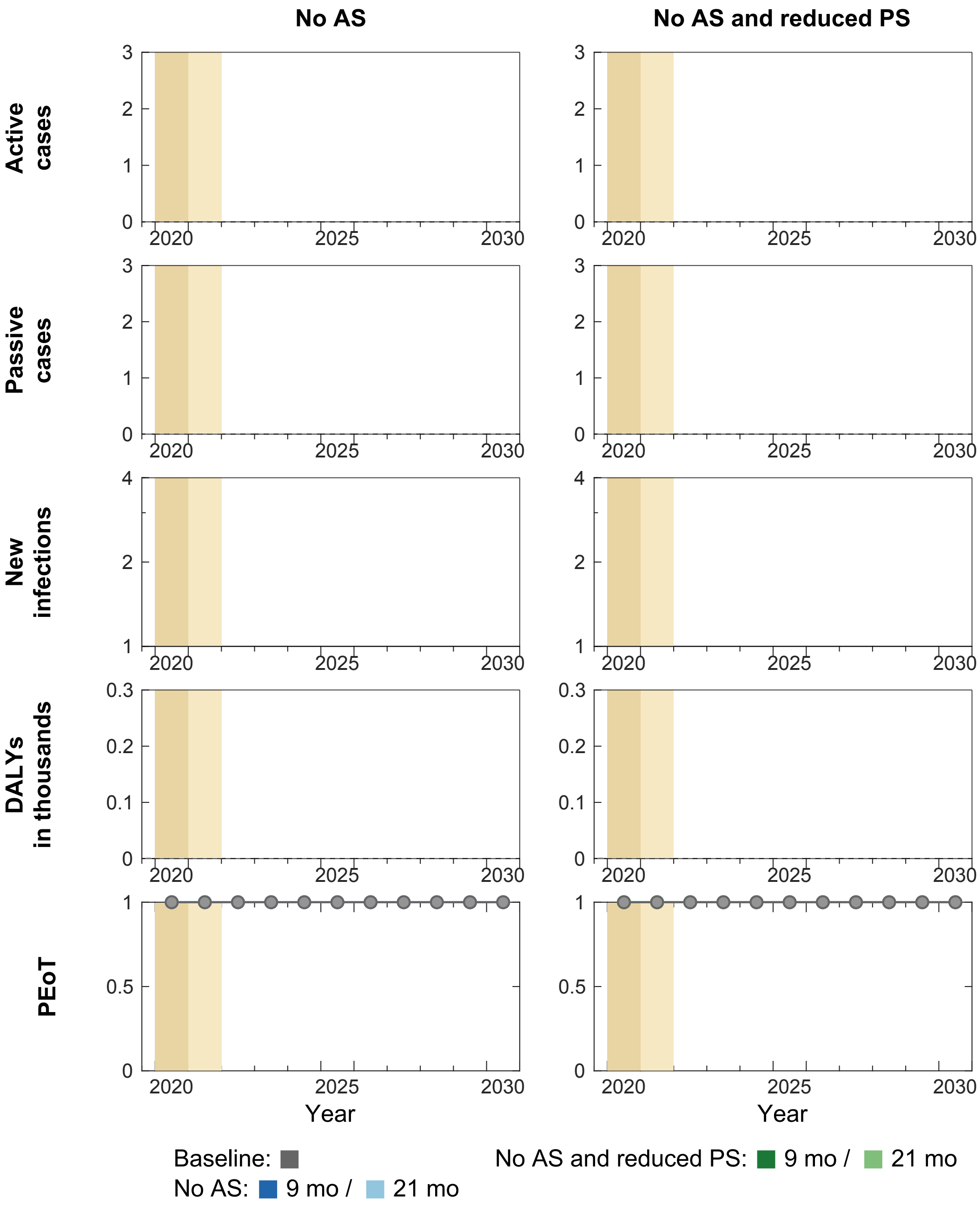

Fig W. Time series of model outputs in Kimbau health zone under the baseline and six interruption scenarios. During 2014–2018, an average of 1% of the population participated in active screening resulting in 0.01 reported cases (from both active and passive screenings) per 10,000 per annum in Kimbau health zone. There are n = 10,000 independent samples, 10 from each of 1,000 independent samples from the joint posterior distributions of the fitted model parameters. Box plots summarise parameter and observational uncertainty. The lines in the boxes represent the medians of predicted results. The lower and upper bounds of the boxes indicate 25th and 75th percentiles. The minimum and maximum values are 2.5th and 97.5th percentiles and therefore whiskers cover 95% prediction intervals.
AS: active screening; PS: passive screening; VC: vector control; DALYs: disability-adjusted life years; PEoT: probability of elimination of transmission

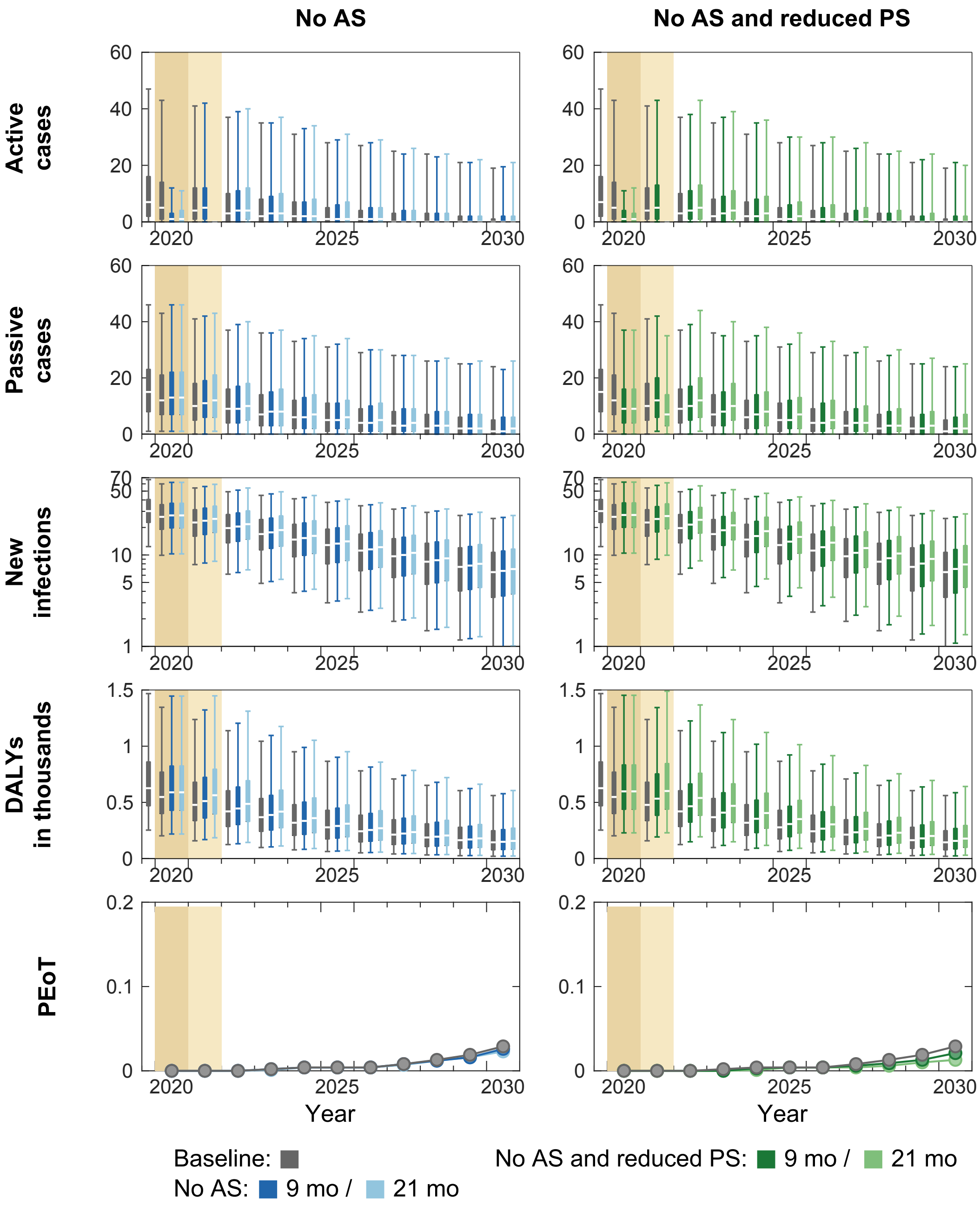

Fig X. Time series of model outputs in Kimputu health zone under the baseline and six interruption scenarios. During 2014–2018, an average of 16% of the population participated in active screening resulting in 2.16 reported cases (from both active and passive screenings) per 10,000 per annum in Kimputu health zone. There are n = 10,000 independent samples, 10 from each of 1,000 independent samples from the joint posterior distributions of the fitted model parameters. Box plots summarise parameter and observational uncertainty. The lines in the boxes represent the medians of predicted results. The lower and upper bounds of the boxes indicate 25th and 75th percentiles. The minimum and maximum values are 2.5th and 97.5th percentiles and therefore whiskers cover 95% prediction intervals.

AS: active screening; PS: passive screening; VC: vector control; DALYs: disability-adjusted life years; PEoT: probability of elimination of transmission

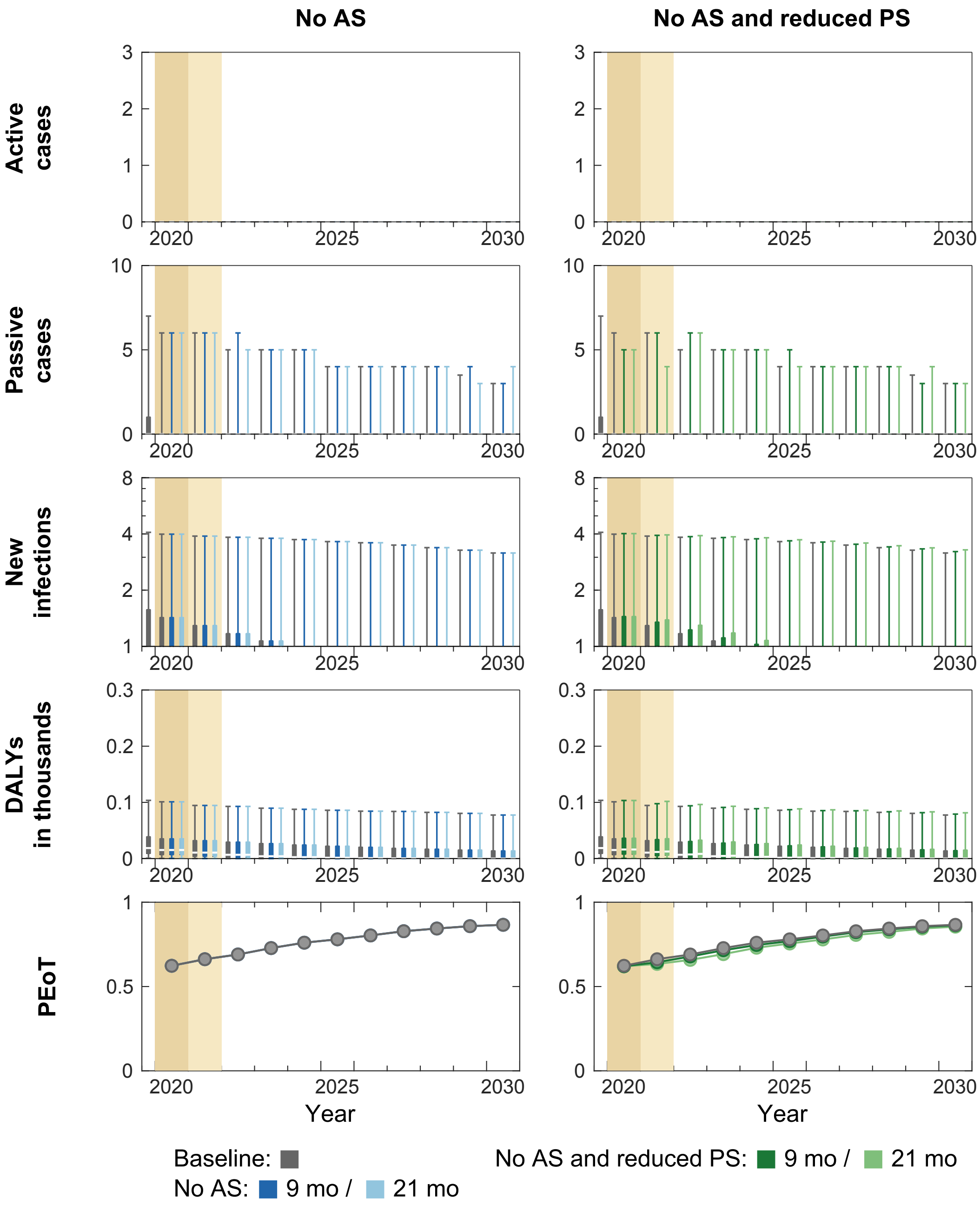

Fig Y. Time series of model outputs in Kiri health zone under the baseline and six interruption scenarios. During 2014–2018, an average of 0% of the population participated in active screening resulting in 0.05 reported cases (from both active and passive screenings) per 10,000 per annum in Kiri health zone. There are n = 10,000 independent samples, 10 from each of 1,000 independent samples from the joint posterior distributions of the fitted model parameters. Box plots summarise parameter and observational uncertainty. The lines in the boxes represent the medians of predicted results. The lower and upper bounds of the boxes indicate 25th and 75th percentiles. The minimum and maximum values are 2.5th and 97.5th percentiles and therefore whiskers cover 95% prediction intervals.

AS: active screening; PS: passive screening; VC: vector control; DALYs: disability-adjusted life years; PEoT: probability of elimination of transmission

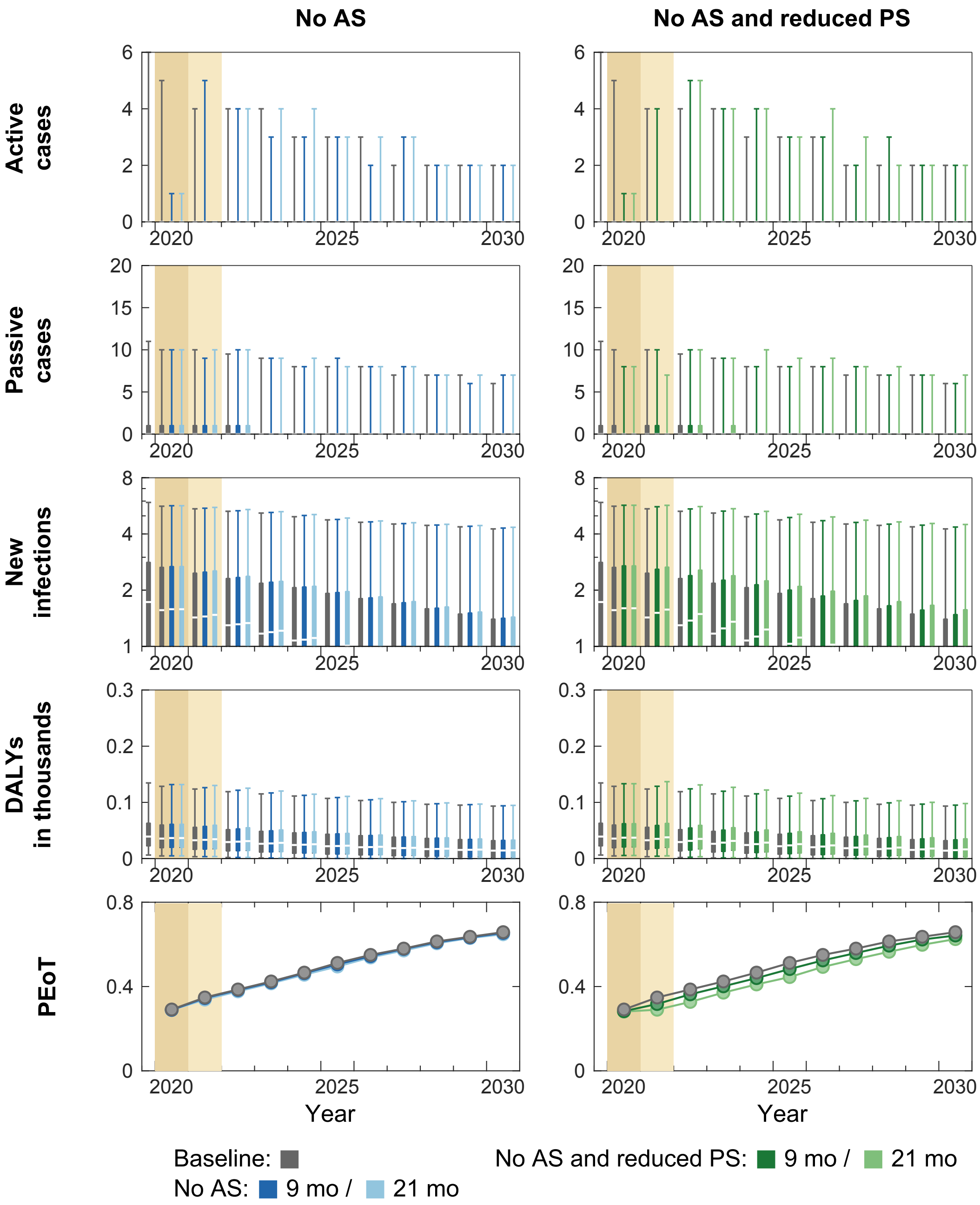

Fig Z. Time series of model outputs in Koshibanda health zone under the baseline and six interruption scenarios. During 2014–2018, an average of 8% of the population participated in active screening resulting in 0.5 reported cases (from both active and passive screenings) per 10,000 per annum in Koshibanda health zone. There are n = 10,000 independent samples, 10 from each of 1,000 independent samples from the joint posterior distributions of the fitted model parameters. Box plots summarise parameter and observational uncertainty. The lines in the boxes represent the medians of predicted results. The lower and upper bounds of the boxes indicate 25th and 75th percentiles. The minimum and maximum values are 2.5th and 97.5th percentiles and therefore whiskers cover 95% prediction intervals.
AS: active screening; PS: passive screening; VC: vector control; DALYs: disability-adjusted life years; PEoT: probability of elimination of transmission

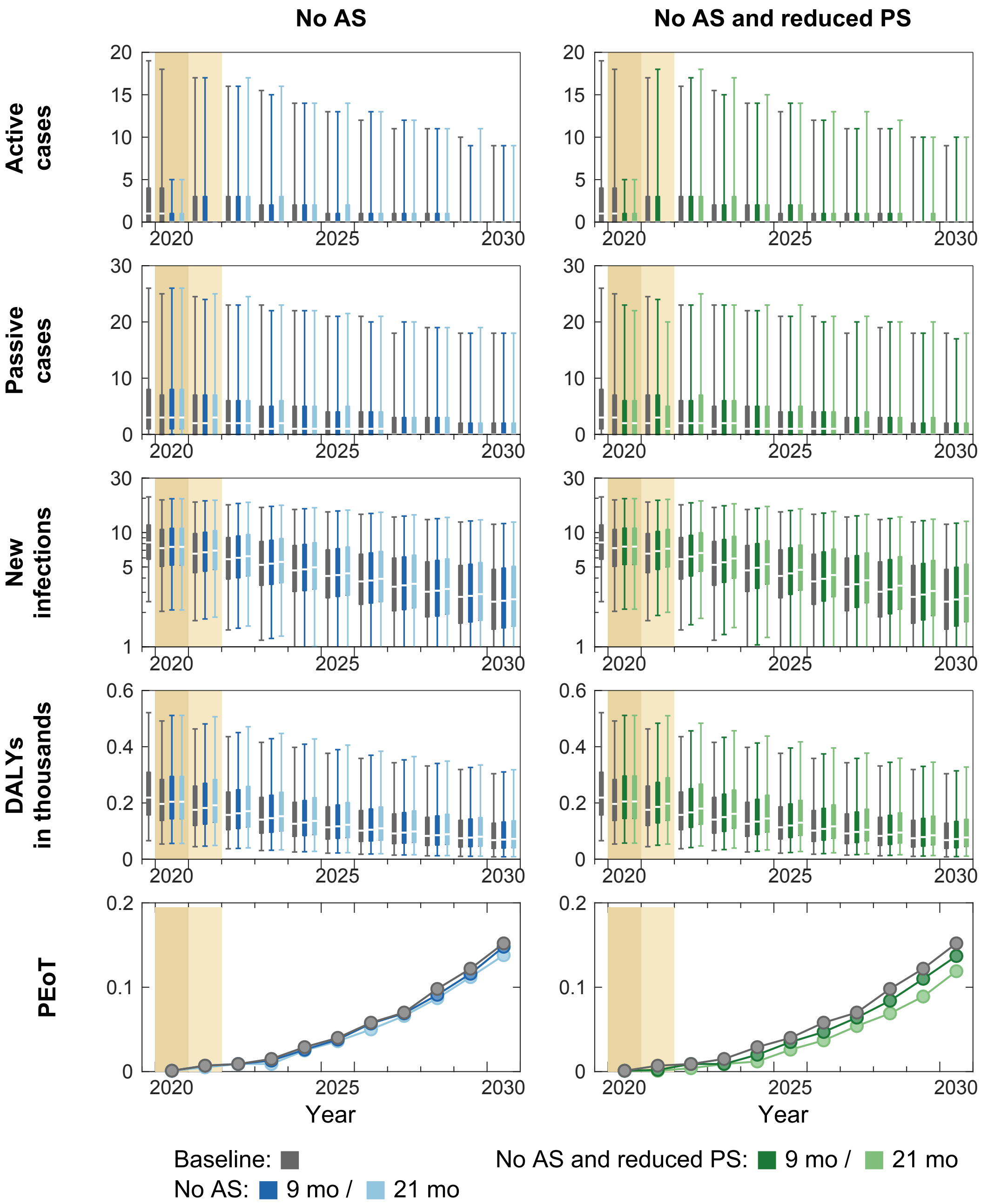

Fig AA. Time series of model outputs in Lusanga health zone under the baseline and six interruption scenarios. During 2014–2018, an average of 8% of the population participated in active screening resulting in 0.23 reported cases (from both active and passive screenings) per 10,000 per annum in Lusanga health zone. There are n = 10,000 independent samples, 10 from each of 1,000 independent samples from the joint posterior distributions of the fitted model parameters. Box plots summarise parameter and observational uncertainty. The lines in the boxes represent the medians of predicted results. The lower and upper bounds of the boxes indicate 25th and 75th percentiles. The minimum and maximum values are 2.5th and 97.5th percentiles and therefore whiskers cover 95% prediction intervals.
AS: active screening; PS: passive screening; VC: vector control; DALYs: disability-adjusted life years; PEoT: probability of elimination of transmission

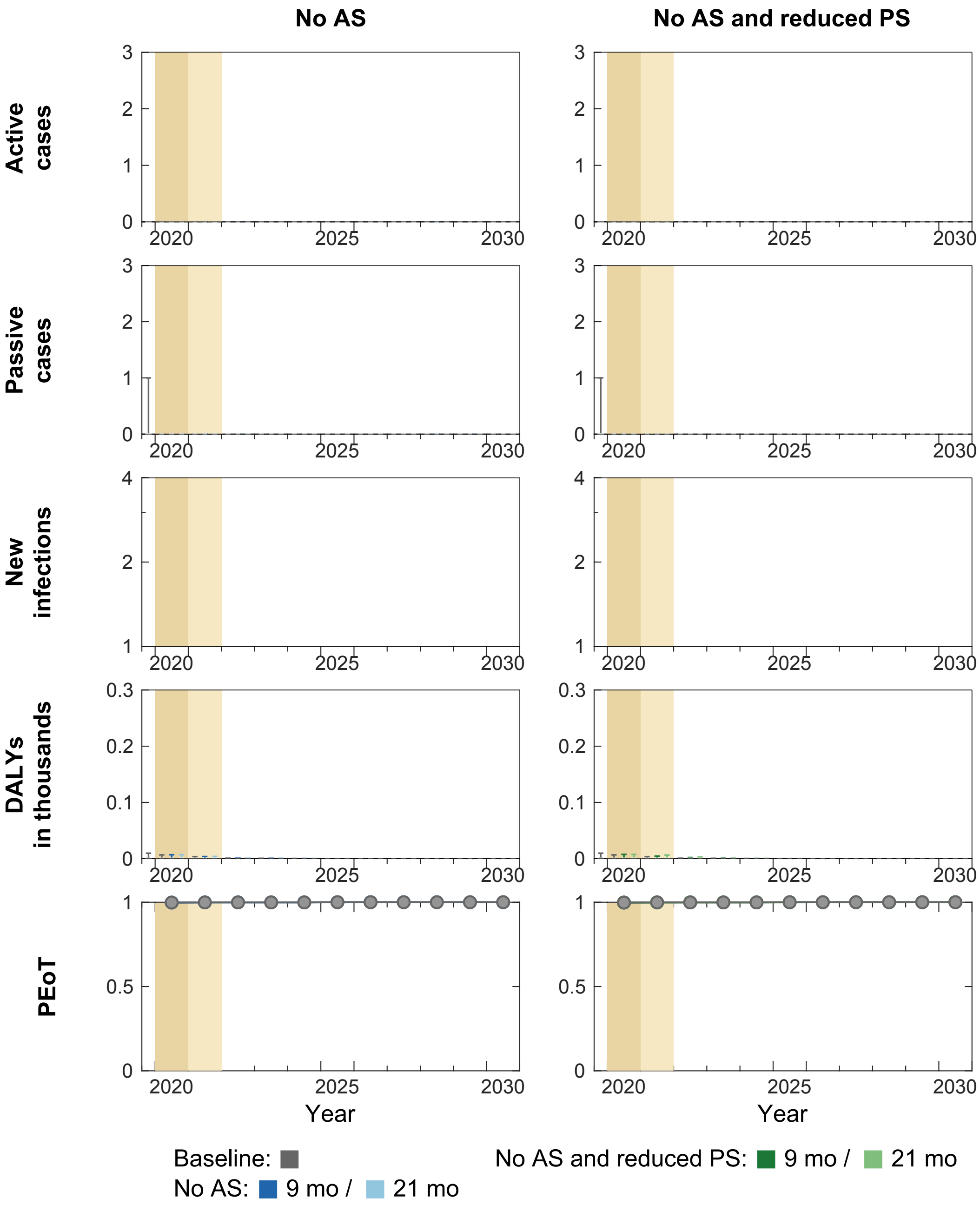

Fig AB. Time series of model outputs in Moanza health zone under the baseline and six interruption scenarios. During 2014–2018, an average of 8% of the population participated in active screening resulting in 0.21 reported cases (from both active and passive screenings) per 10,000 per annum in Moanza health zone. There are n = 10,000 independent samples, 10 from each of 1,000 independent samples from the joint posterior distributions of the fitted model parameters. Box plots summarise parameter and observational uncertainty. The lines in the boxes represent the medians of predicted results. The lower and upper bounds of the boxes indicate 25th and 75th percentiles. The minimum and maximum values are 2.5th and 97.5th percentiles and therefore whiskers cover 95% prediction intervals.

AS: active screening; PS: passive screening; VC: vector control; DALYs: disability-adjusted life years; PEoT: probability of elimination of transmission

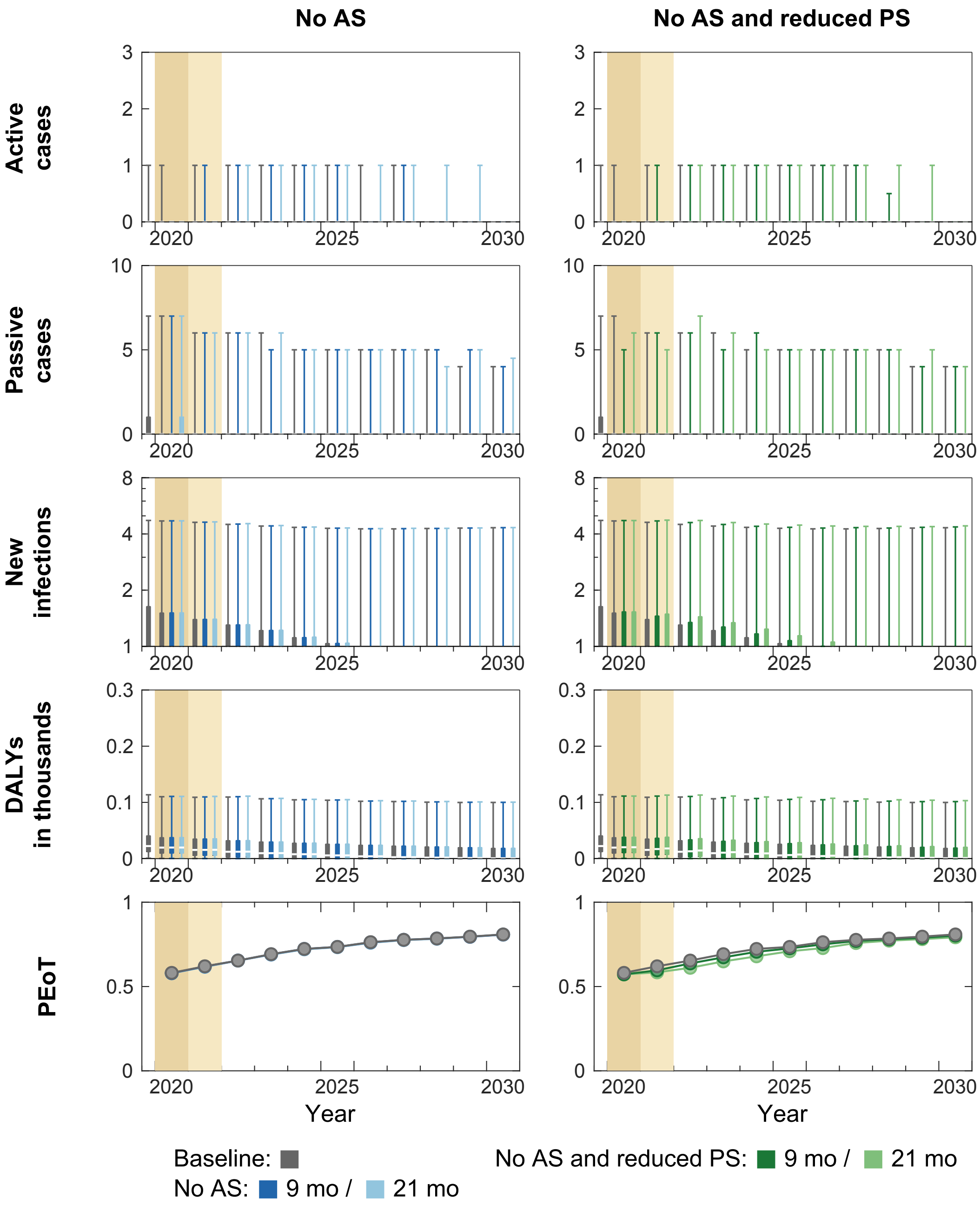

Fig AC. Time series of model outputs in Mungindu health zone under the baseline and six interruption scenarios. During 2014–2018, an average of 2% of the population participated in active screening resulting in 0.11 reported cases (from both active and passive screenings) per 10,000 per annum in Mungindu health zone. There are n = 10,000 independent samples, 10 from each of 1,000 independent samples from the joint posterior distributions of the fitted model parameters. Box plots summarise parameter and observational uncertainty. The lines in the boxes represent the medians of predicted results. The lower and upper bounds of the boxes indicate 25th and 75th percentiles. The minimum and maximum values are 2.5th and 97.5th percentiles and therefore whiskers cover 95% prediction intervals.

AS: active screening; PS: passive screening; VC: vector control; DALYs: disability-adjusted life years; PEoT: probability of elimination of transmission

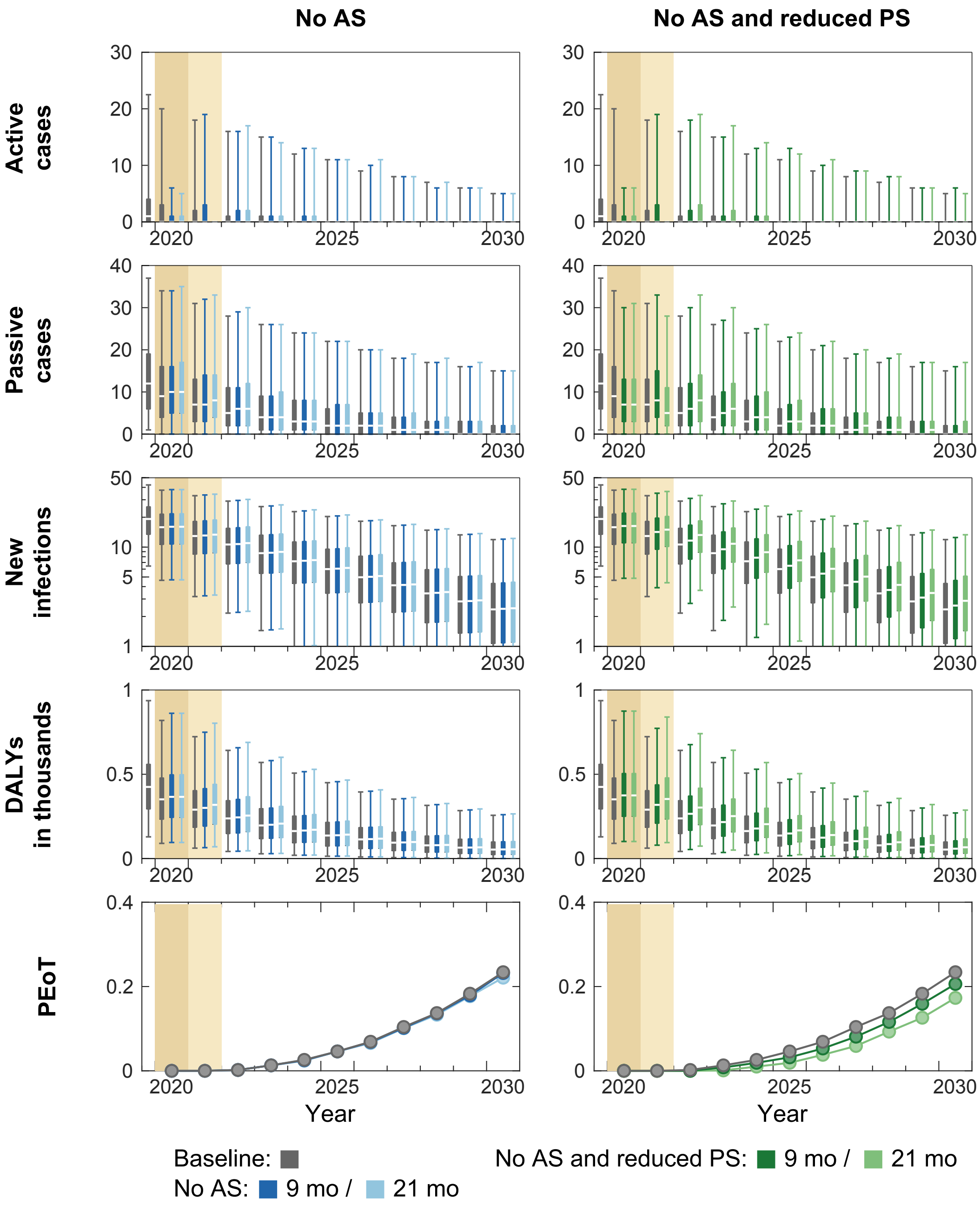

Fig AD. Time series of model outputs in Nioki health zone under the baseline and six interruption scenarios. During 2014–2018, an average of 16% of the population participated in active screening resulting in 2.82 reported cases (from both active and passive screenings) per 10,000 per annum in Nioki health zone. There are n = 10,000 independent samples, 10 from each of 1,000 independent samples from the joint posterior distributions of the fitted model parameters. Box plots summarise parameter and observational uncertainty. The lines in the boxes represent the medians of predicted results. The lower and upper bounds of the boxes indicate 25th and 75th percentiles. The minimum and maximum values are 2.5th and 97.5th percentiles and therefore whiskers cover 95% prediction intervals.
AS: active screening; PS: passive screening; VC: vector control; DALYs: disability-adjusted life years; PEoT: probability of elimination of transmission

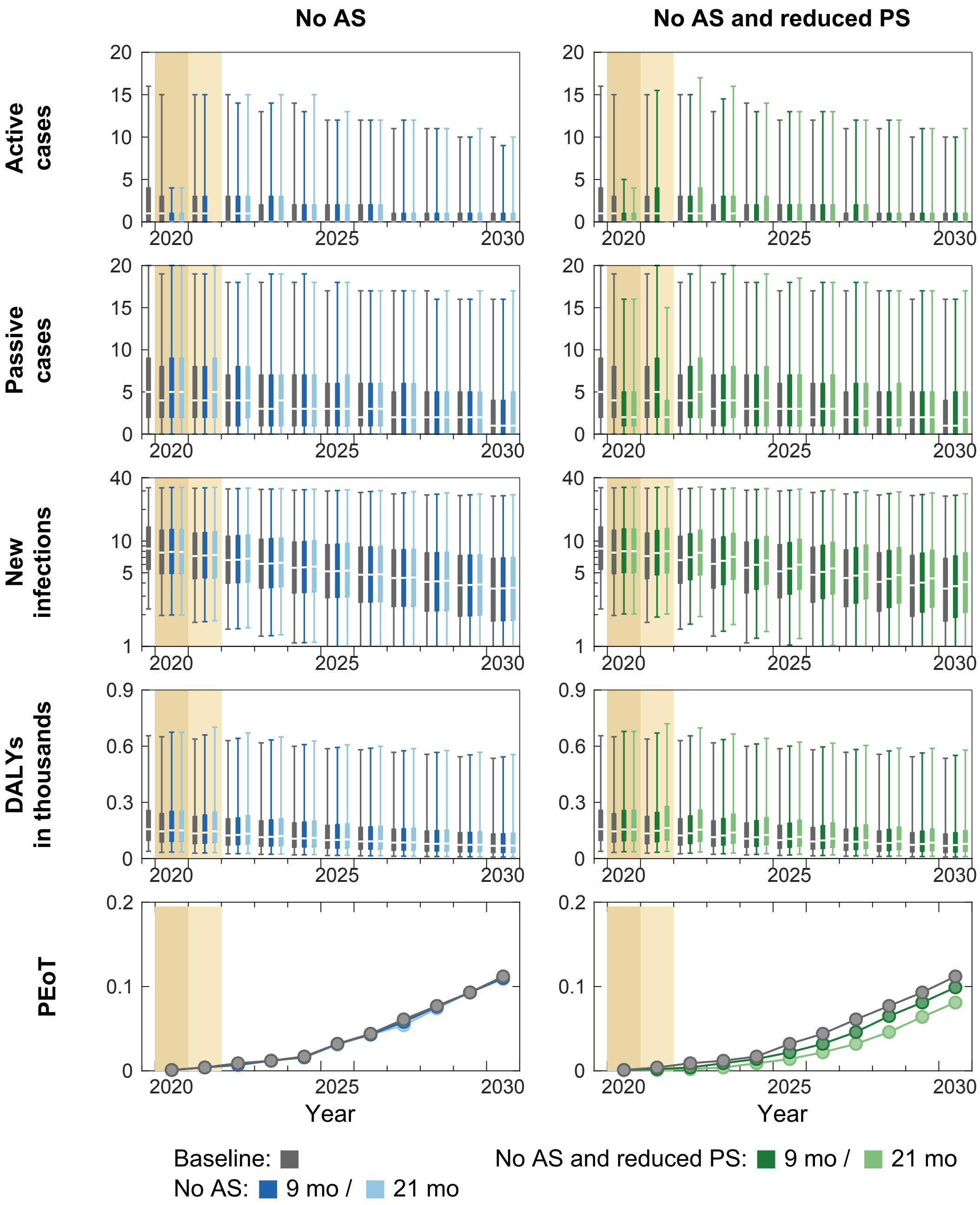

Fig AE. Time series of model outputs in Ntand Embelo health zone under the baseline and six interruption scenarios. During 2014–2018, an average of 16% of the population participated in active screening resulting in 2.69 reported cases (from both active and passive screenings) per 10,000 per annum in Ntand Embelo health zone. There are n = 10,000 independent samples, 10 from each of 1,000 independent samples from the joint posterior distributions of the fitted model parameters. Box plots summarise parameter and observational uncertainty. The lines in the boxes represent the medians of predicted results. The lower and upper bounds of the boxes indicate 25th and 75th percentiles. The minimum and maximum values are 2.5th and 97.5th percentiles and therefore whiskers cover 95% prediction intervals.

AS: active screening; PS: passive screening; VC: vector control; DALYs: disability-adjusted life years; PEoT: probability of elimination of transmission

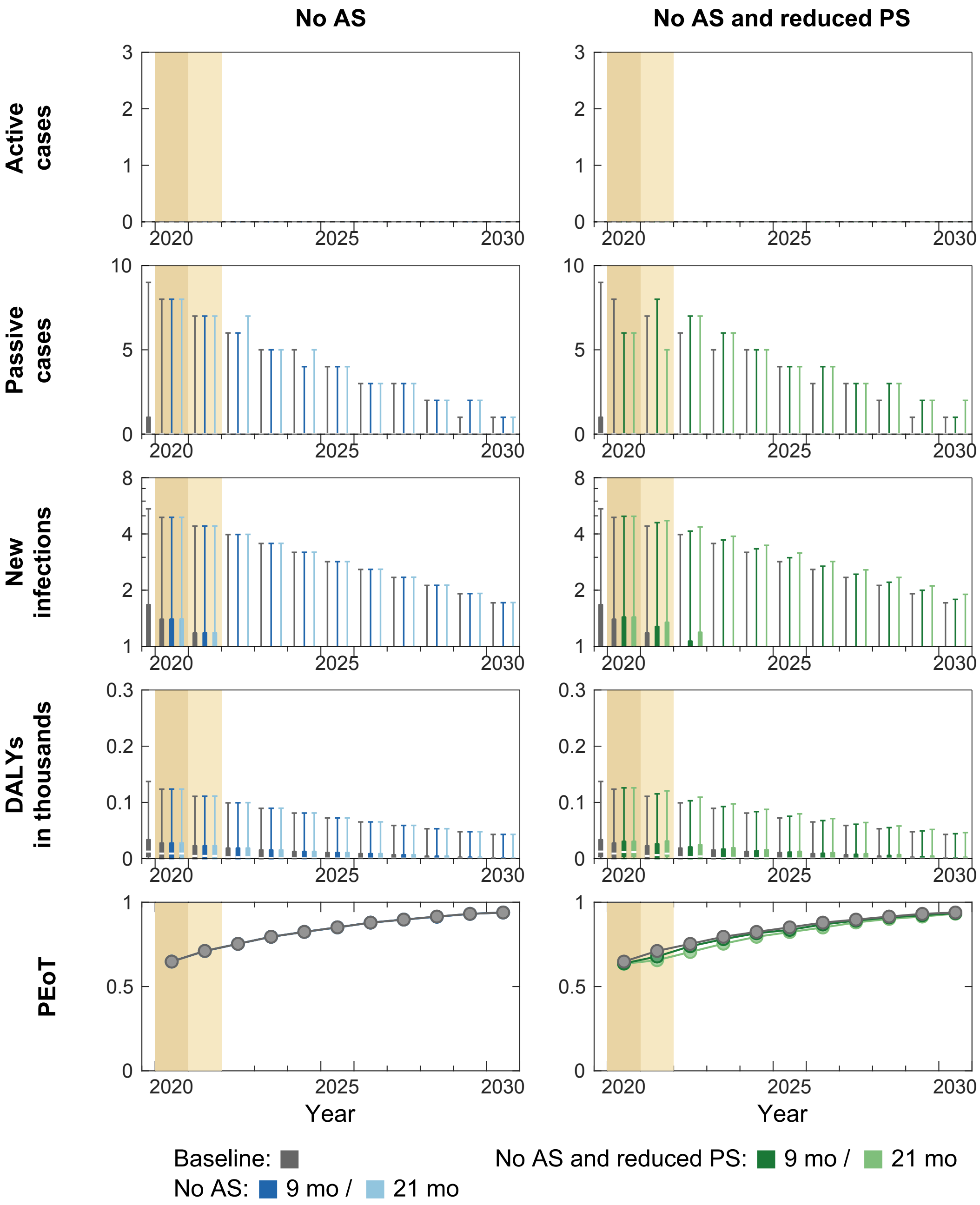

Fig AF. Time series of model outputs in Oshwe health zone under the baseline and six interruption scenarios. During 2014–2018, an average of 0% of the population participated in active screening resulting in 0.08 reported cases (from both active and passive screenings) per 10,000 per annum in Oshwe health zone. There are n = 10,000 independent samples, 10 from each of 1,000 independent samples from the joint posterior distributions of the fitted model parameters. Box plots summarise parameter and observational uncertainty. The lines in the boxes represent the medians of predicted results. The lower and upper bounds of the boxes indicate 25th and 75th percentiles. The minimum and maximum values are 2.5th and 97.5th percentiles and therefore whiskers cover 95% prediction intervals.

AS: active screening; PS: passive screening; VC: vector control; DALYs: disability-adjusted life years; PEoT: probability of elimination of transmission

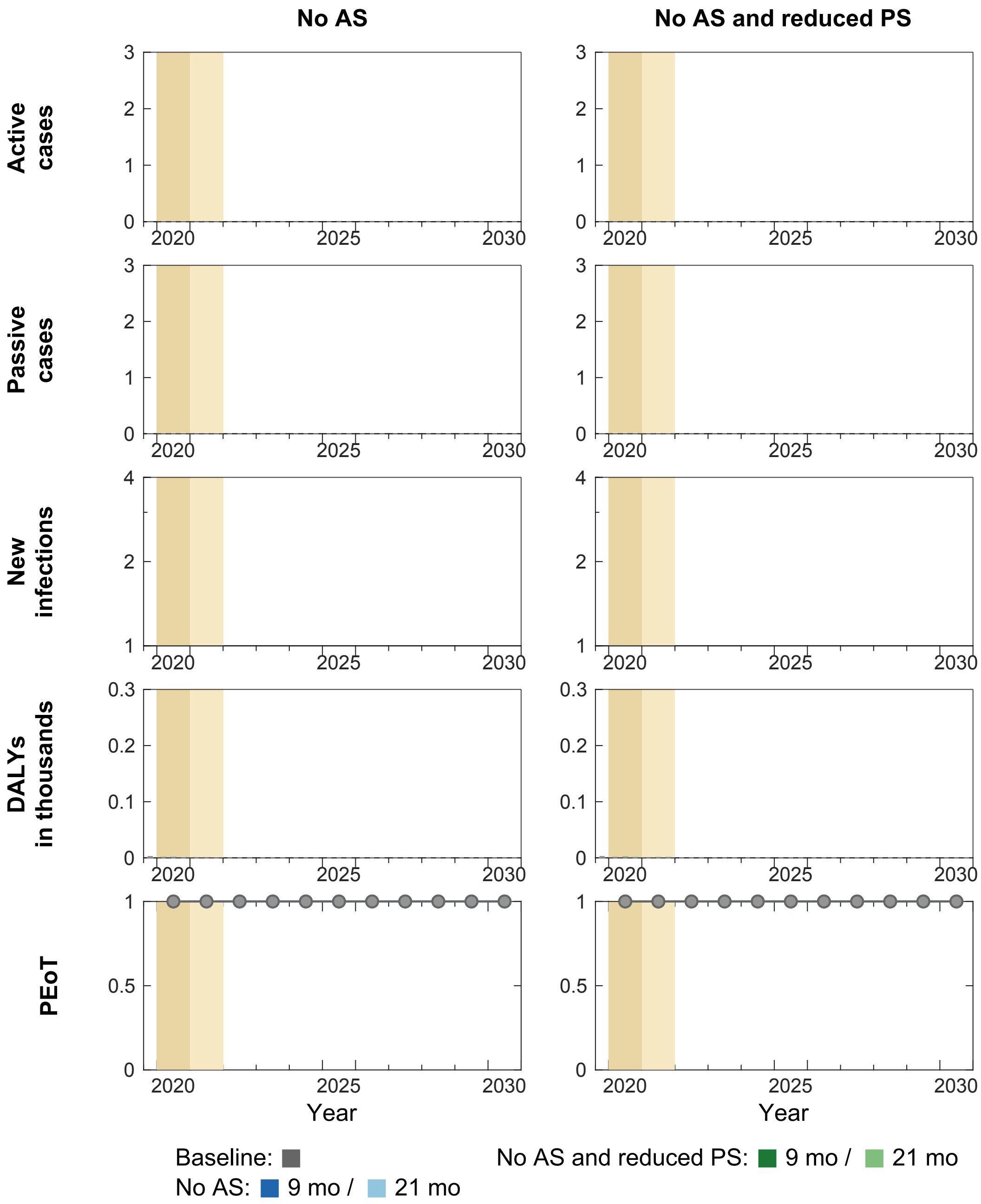

Fig AG. Time series of model outputs in Pay Kongila health zone under the baseline and six interruption scenarios. During 2014–2018, an average of 0% of the population participated in active screening resulting in 0.02 reported cases (from both active and passive screenings) per 10,000 per annum in Pay Kongila health zone. There are n = 10,000 independent samples, 10 from each of 1,000 independent samples from the joint posterior distributions of the fitted model parameters. Box plots summarise parameter and observational uncertainty. The lines in the boxes represent the medians of predicted results. The lower and upper bounds of the boxes indicate 25th and 75th percentiles. The minimum and maximum values are 2.5th and 97.5th percentiles and therefore whiskers cover 95% prediction intervals.
AS: active screening; PS: passive screening; VC: vector control; DALYs: disability-adjusted life years; PEoT: probability of elimination of transmission

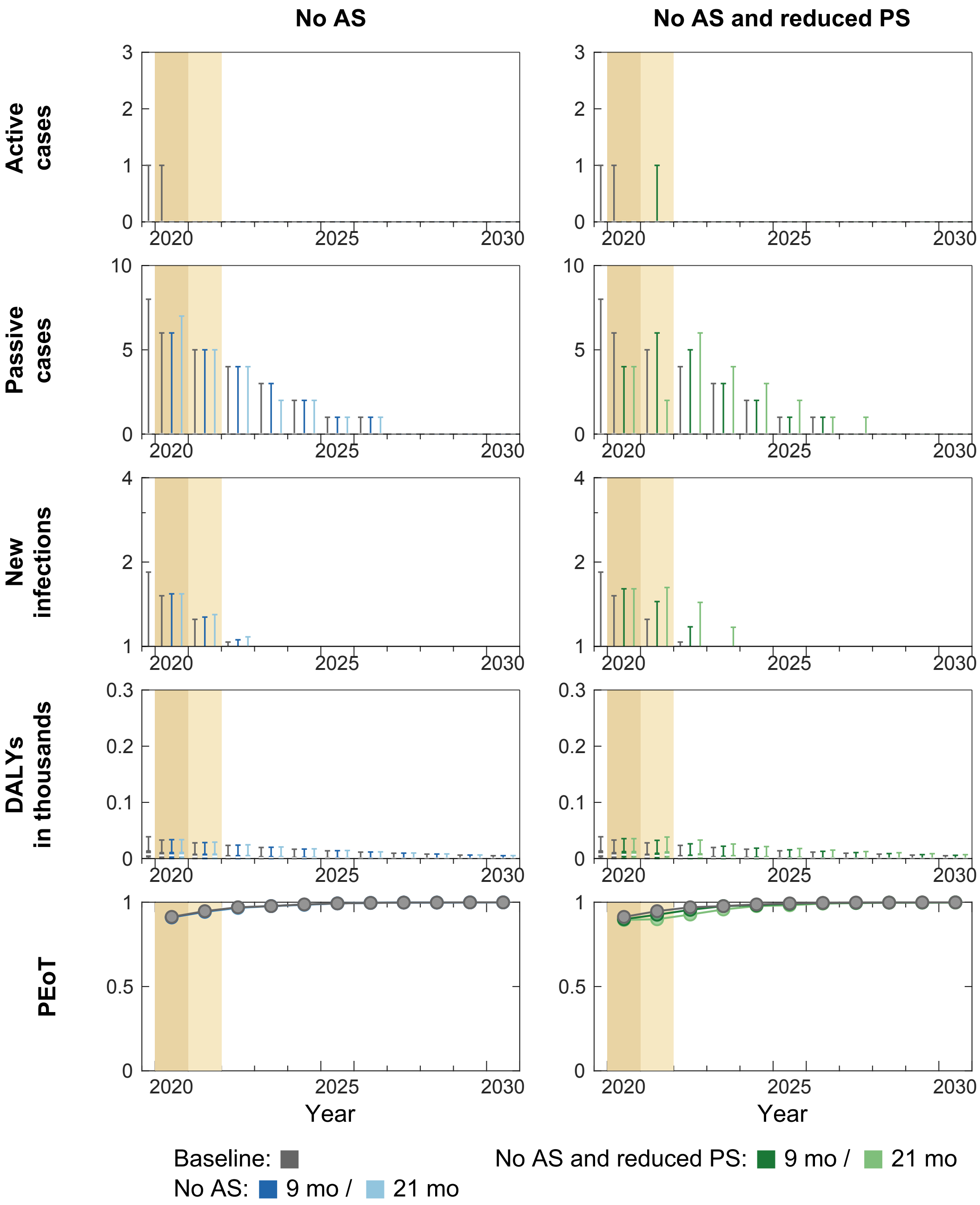

Fig AH. Time series of model outputs in Popokabaka health zone under the baseline and six interruption scenarios. During 2014–2018, an average of 4% of the population participated in active screening resulting in 0.53 reported cases (from both active and passive screenings) per 10,000 per annum in Popokabaka health zone. There are n = 10,000 independent samples, 10 from each of 1,000 independent samples from the joint posterior distributions of the fitted model parameters. Box plots summarise parameter and observational uncertainty. The lines in the boxes represent the medians of predicted results. The lower and upper bounds of the boxes indicate 25th and 75th percentiles. The minimum and maximum values are 2.5th and 97.5th percentiles and therefore whiskers cover 95% prediction intervals.
AS: active screening; PS: passive screening; VC: vector control; DALYs: disability-adjusted life years; PEoT: probability of elimination of transmission

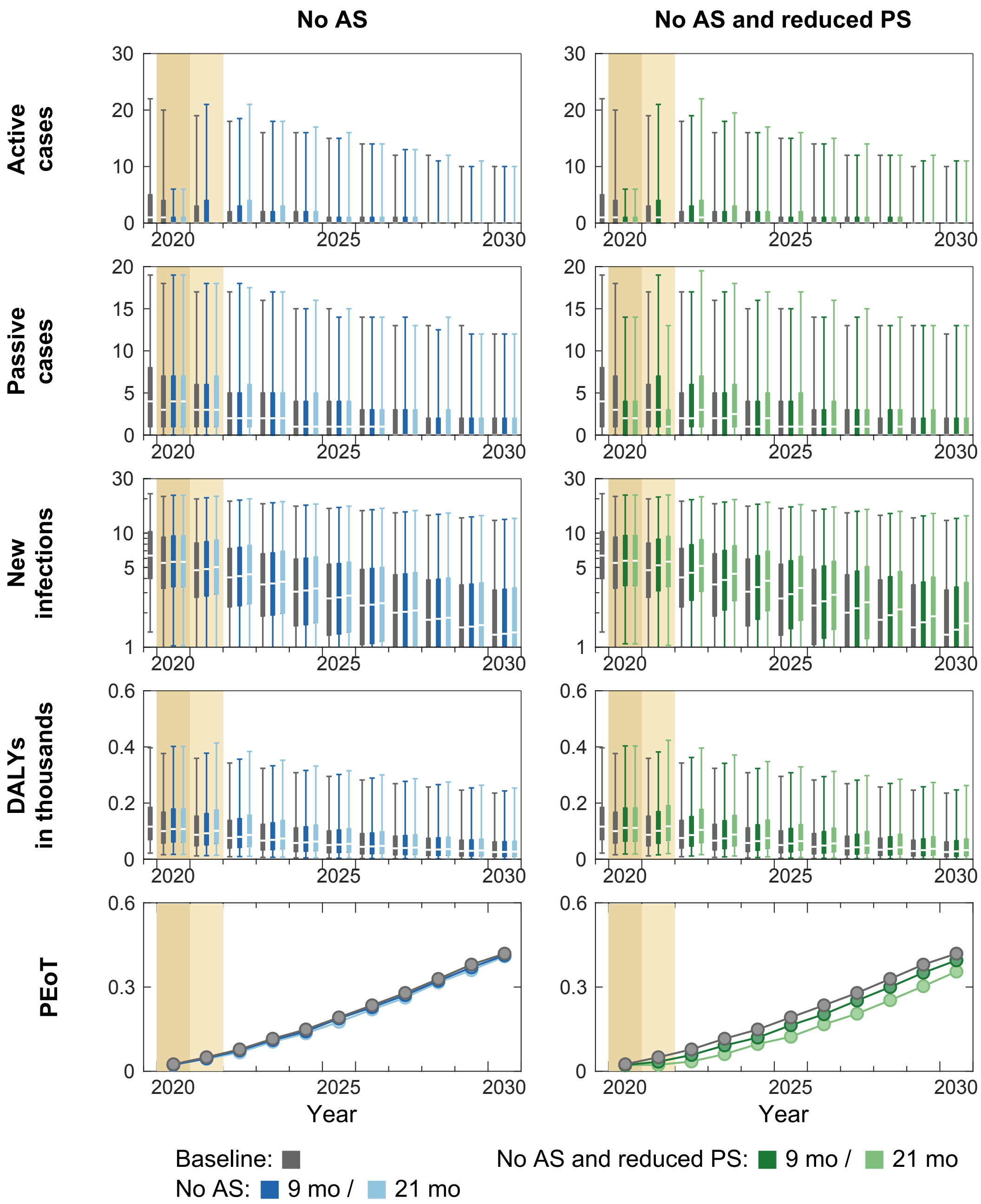

Fig AI. Time series of model outputs in Sia health zone under the baseline and six interruption scenarios. During 2014–2018, an average of 19% of the population participated in active screening resulting in 1.02 reported cases (from both active and passive screenings) per 10,000 per annum in Sia health zone. There are n = 10,000 independent samples, 10 from each of 1,000 independent samples from the joint posterior distributions of the fitted model parameters. Box plots summarise parameter and observational uncertainty. The lines in the boxes represent the medians of predicted results. The lower and upper bounds of the boxes indicate 25th and 75th percentiles. The minimum and maximum values are 2.5th and 97.5th percentiles and therefore whiskers cover 95% prediction intervals. AS: active screening; PS: passive screening; VC: vector control; DALYs: disability-adjusted life years; PEoT: probability of elimination of transmission

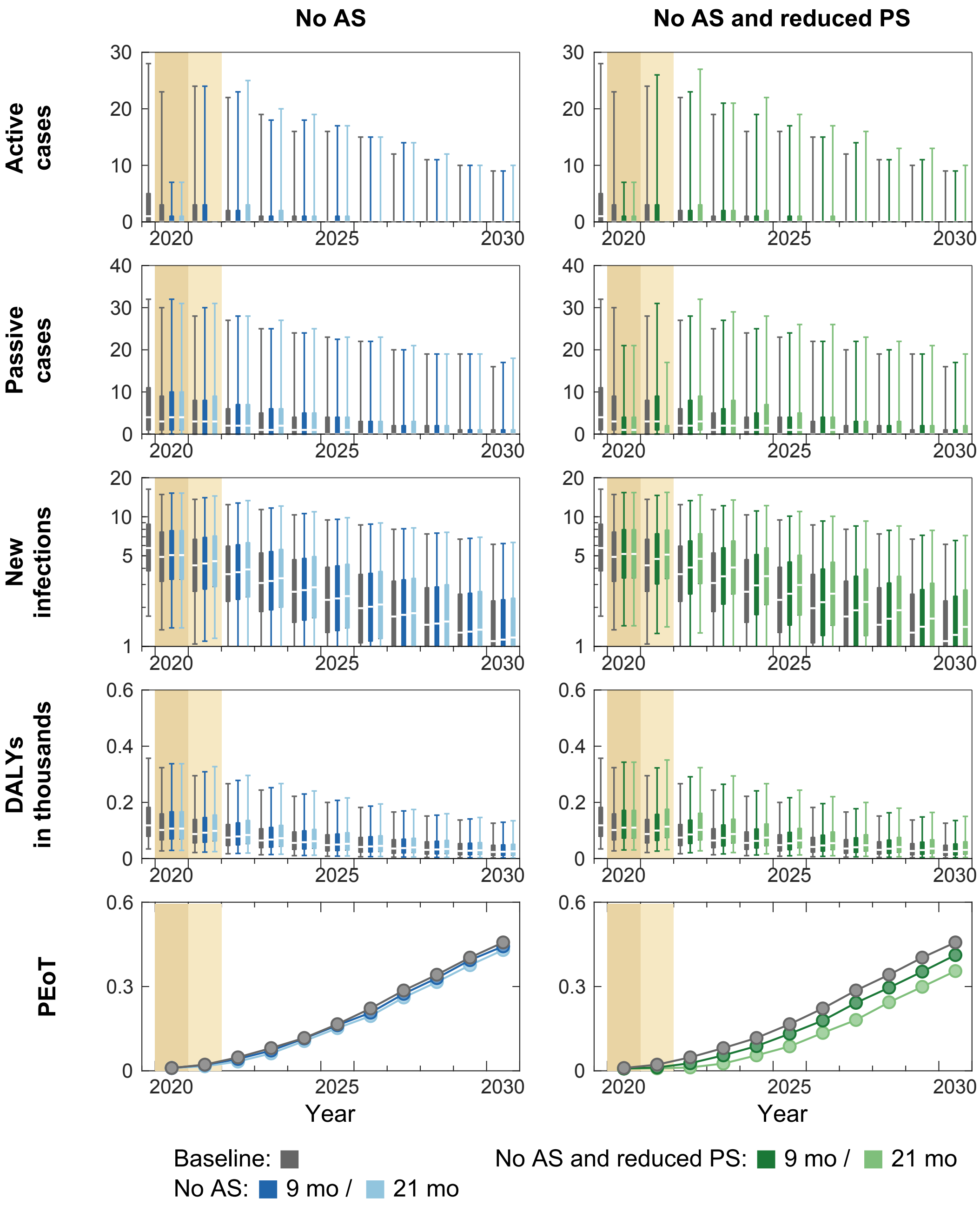

Fig AJ. Time series of model outputs in Vanga health zone under the baseline and six interruption scenarios. During 2014–2018, an average of 12% of the population participated in active screening resulting in 0.42 reported cases (from both active and passive screenings) per 10,000 per annum in Vanga health zone. There are n = 10,000 independent samples, 10 from each of 1,000 independent samples from the joint posterior distributions of the fitted model parameters. Box plots summarise parameter and observational uncertainty. The lines in the boxes represent the medians of predicted results. The lower and upper bounds of the boxes indicate 25th and 75th percentiles. The minimum and maximum values are 2.5th and 97.5th percentiles and therefore whiskers cover 95% prediction intervals.
AS: active screening; PS: passive screening; VC: vector control; DALYs: disability-adjusted life years; PEoT: probability of elimination of transmission

Health zone	YEoT under baseline	Median delay in YEoT (in years)					
		No AS		No AS and reduced PS		No AS or VC and reduced PS	
		9 months	21 months	9 months	21 months	9 months	21 months
Yasa Bonga	2017	0	0	0	0	0	0
Masi Manimba	2021	0	0	0	0	1	2
Bandundu	2020	0	0	0	0	1	2
Kikongo	2021	0	0	0	0	1	2
Kwamouth	2022	0	0	0	0	1	2
Bokoro	2023	0	0	0	0	0	1
Bolobo	2021	0	0	0	0	1	2
Bulungu	2022	0	0	0	0	1	2
Mokala	2023	0	0	0	0	0	1
Mushie	2023	0	0	0	0	0	1
Yumbi	2022	0	0	0	0	0	1
Bagata	2039	0	0	1	1		
Bandjau	>2050	0	0	1	2		
Boko	2018	0	0	0	0		
Bosobe	2022	0	0	0	1		
Djuma	2036	0	0	1	2		
Idiofa	2047	0	0	1	2		
Inongo	2019	0	0	0	0		
Ipamu	2038	0	0	1	2		
Kasongolunda	2015	0	0	0	0		
Kenge	2040	0	0	1	1		
Kikwit Nord	2020	0	0	0	0		
Kikwit Sud	2015	0	0	0	0		
Kimbau	2011	0	0	0	0		
Kimputu	2045	0	1	1	2	Same as No AS and reduced PS	
Kiri	2018	0	0	0	0		
Koshibanda	2025	0	0	0	1		
Lusanga	2040	0	1	1	1		
Moanza	2013	0	0	0	0		
Mosango	2027	0	1	1	1		
Mungindu	2019	0	0	0	0		
Nioki	2035	0	0	0	1		
Ntand Embelo	2048	0	0	1	2		
Oshwe	2019	0	0	0	0		
Pay Kongila	2011	0	0	0	0		
Popokabaka	2017	0	0	0	0		
Sia	2032	0	0	1	2		
Vanga	2031	0	0	1	2		

Table A. Expected year of elimination of transmission (YEoT) and delay to YEoT under interruption scenarios for all health zones of the former Bandundu province. Blue shading represents health zones with on-going vector control (VC) before 2020, yellow shading represents those with planned VC, and orange represents those with no planned or on-going VC. In the health zones with no planned or on-going VC the *No AS or VC and reduced PS* scenario is identical to *No AS and reduced PS*.
AS: active screening; PS: passive screening; VC: vector control; YEoT: year of elimination of transmission

Health zone	Median DALYs under baseline	Additional DALYs accrued (median)					
		No AS		No AS and reduced PS		No AS or VC and reduced PS	
		9 months	21 months	9 months	21 months	9 months	21 months
Yasa Bonga	47.0	<0.1	0.1	10.9	22.3	10.9	22.3
Masi Manimba	868.8	56.1	103.3	144.4	289.5	165.4	369.6
Bandundu	182.7	17.3	27.8	46.6	90.7	56.1	123.4
Kikongo	1131.1	17.7	43.9	84.6	182.6	92.1	218.1
Kwamouth	1445.9	160.4	268.8	365.9	727.5	463.7	1069.3
Bokoro	3221.4	22.7	58.8	190.5	476.5	190.5	631.4
Bolobo	148.7	15.0	26.2	64.7	129.2	106	232.2
Bulungu	1091.4	62.0	133.6	191.2	394.5	401.9	847.7
Mokala	1085.6	47.2	104.6	131.3	284.5	131.3	516.6
Mushie	1516.2	50.4	112.6	117.0	249.8	117.0	538.7
Yumbi	495.8	14.0	32.3	52.6	120.3	52.6	166.2
Bagata	2421.3	92.0	216.4	303.5	652.8		
Bandjau	3433.8	16.0	33.0	32.3	76.5		
Boko	18.5	0.3	0.5	3.1	6.5		
Bosobe	167.5	9.4	18.9	19.0	43.9		
Djuma	1371.1	74.4	176.3	199.9	451.2		
Idiofa	1119.5	29.1	63.4	71.2	168.9		
Inongo	27.6	<0.1	<0.1	8.8	24.8		
Ipamu	1151.5	39.2	76.3	141.9	332.9		
Kasongolunda	6.5	<0.1	<0.1	2.7	7.0		
Kenge	1945.2	99.1	204.8	187.3	417.3		
Kikwit Nord	22.3	<0.1	<0.1	10.5	27.9		
Kikwit Sud	0.9	<0.1	<0.1	0.6	1.4		
Kimbau	<0.1	<0.1	<0.1	<0.1	<0.1	Same as No AS and reduced PS	
Kimputu	3376.2	197.4	404.6	357.0	774.2		
Kiri	42.2	<0.1	<0.1	3.9	7.6		
Koshibanda	255.0	5.1	13.0	15.3	38.8		
Lusanga	1326.7	44.4	97.4	78.0	172.1		
Moanza	<0.1	<0.1	<0.1	<0.1	<0.1		
Mosango	322.8	35.2	76.3	61.4	152.7		
Mungindu	81.3	0.3	0.6	9.3	17.2		
Nioki	1769.6	47.3	96.7	169.4	401.3		
Ntand Embelo	1125.9	14.9	34.0	72.1	180.8		
Oshwe	18.7	<0.1	<0.1	4.3	8.4		
Pay Kongila	<0.1	<0.1	<0.1	<0.1	<0.1		
Popokabaka	14.1	0.3	0.5	3.6	8.0		
Sia	610.3	22.0	55.7	67.7	163.0		
Vanga	593.2	28.1	61.8	75.4	172.0		

Table B. Total median disability-adjusted life years (DALYs) under baseline and additional DALYs accrued under interruption scenarios between 2020 and 2030 for all health zones of the former Bandundu province. Blue shading represents health zones with on-going vector control (VC) before 2020, yellow shading represents those with planned VC, and orange represents those with no planned or on-going VC. In the health zones with no planned or on-going VC the *No AS or VC, and reduced PS* scenario is identical to *No AS and reduced PS*.
AS: active screening; PS: passive screening; VC: vector control; DALYs: disability-adjusted life years

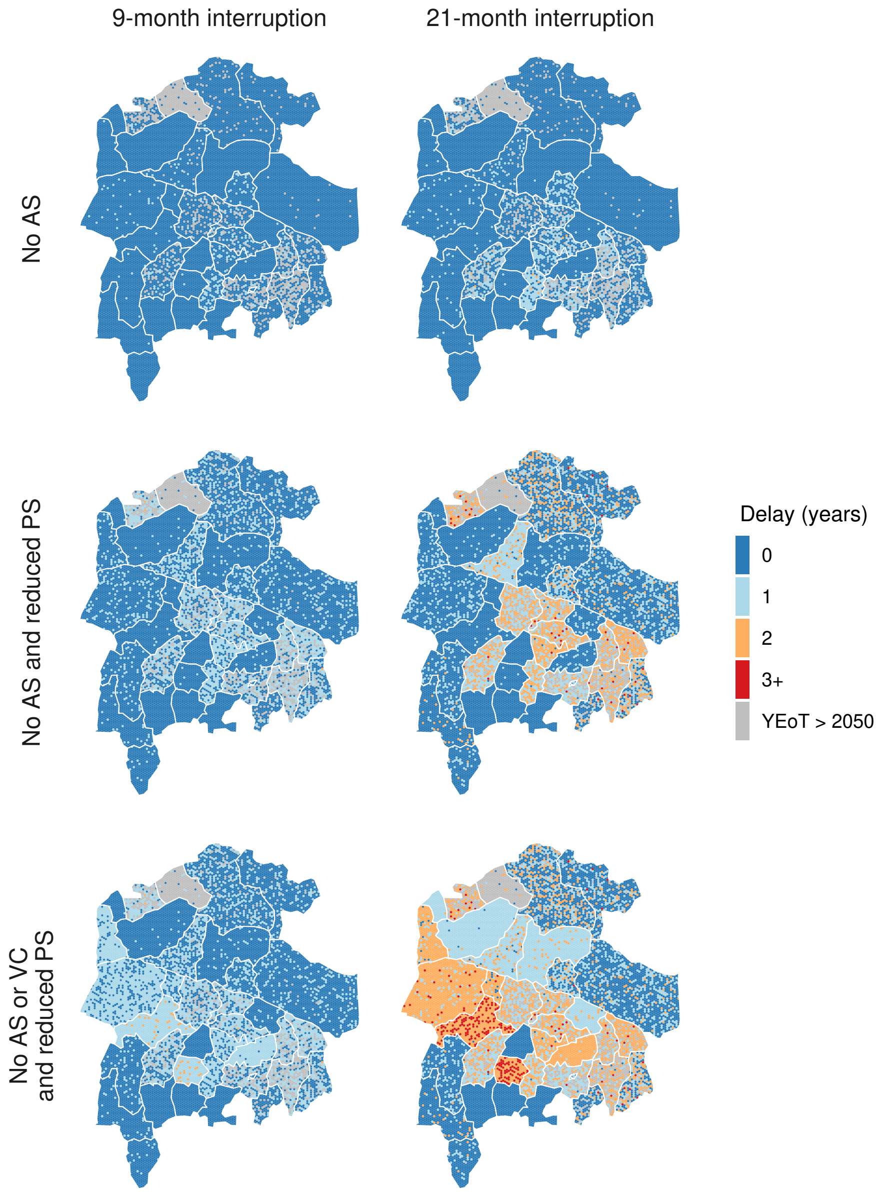

Fig AK. Representation of within-health zone distribution of delay in YEoT under six interruption scenarios. Colours of individual hexagons represent a random sample from the posterior distribution for that health zone. 'YEoT ¿ 2050' where the realised YEoT, either baseline, interruption, or both, was beyond 2050. In each health zone, the baseline strategy is either *MeanAS* or *MeanAS+VC* depending on its VC status (summarised in Table 1 in the main text). Shapefiles used to produce these maps were provided by Nicole Hoff and Cyrus Sinai under a CC-BY licence (current versions can be found at `https://data.humdata.org/dataset/drc-health-data`).
AS: active screening; PS: passive screening; VC: vector control; YEoT: year of elimination of transmission

References

1. Abomo P, Miaka EM, Crossman SJ, Hope A. Demonstrating the sustainability of capacity strengthening amidst COVID-19. International Health. 2021;13:480–481.
