# Supplementary material for: A modelling assessment of short- and medium-term risks of programme interruptions for gambiense human African trypanosomiasis in the DRC
Source: PLoS Negl Trop Dis. 2023 Apr 28;17(4):e0011299. doi: 10.1371/journal.pntd.0011299 (PMC10171604; doi:10.1371/journal.pntd.0011299)
Supplement: Attachment — Submitted filename: PLoSNTDs_Reviews.docx [file pntd.0011299.s004.docx]

**Reviewer Comments**

**Methods**
Reviewer #1:

The objectives of the study are not openly stated. Their resemblance simply run as statements in the text. These need to be stated clearly in objectives section to offer the reader with what to expect in the paper.
No hypotheses stated
methods and selected sample size were sufficient and subjected to right statistical analysis.
Model makes use of secondary data majorly. Hence no need for ethical clearance in this case

**Author response:** We added a subsection of “Research objectives” at the end of introduction (lines 86—107). We also added our hypothesis as below in the Research objectives subsection (line 89):
“We hypothesise that there would have been some impact on transmission and case reporting during the interruption period, but it is unclear how big an impact interruption would have given the slow progressing nature of gHAT.”

Reviewer #2:

The article is well written and discusses HAT control in the Democratic Republic of Congo during the COVID-19 lockdown. The objectives of this study are clear and the methods to achieve them are well developed. The authors demonstrate a strong mastery of statistical analyzes on modeling. It would be desirable for the authors to refer to the current epidemiological situation of HAT in the Democratic Republic of Congo because the prevalence of the disease has fallen sharply and it is no longer felt in the population as a health problem. This negatively impacts the rate of participation in active screening activities. It would perhaps be desirable for the authors to take this community aspect into account in their model so that it stands out in the analyzes or as limits.

**Author response:** We agree that community perception can change their behaviours towards interventions. We, therefore, added the following sentences in the Methods section to give a quick overview on recent participation in AS (line 146):

“Our fitted model explicitly uses the number of people tested during AS for 2000—2016, which captures the historical variation in AS coverage. Despite the potential for decreasing interest in attending AS when knowledge of disease is diminishing, data shows that the maximum number of people screened occurred after 2016 in 19 of out 38 health zones in Bandundu Nord and Sud coordinations. Our future projections use the average of known 2014—2018 AS coverage for each health zone as the future coverage to reflect the observed trends in participation in recent years.”

We also stated our limitations related to AS and what we can do when more data becomes available in the future in the Discussion section (line 575):

“The medium- to long-term projections could be influenced by model assumptions, it would also be possible to refit the model to the actual screening and case data to quantify if there is any statistical evidence of the interruption's impact on PS during 2020. Additionally, we can also factor in some plausible changes such as the decrease in AS participation caused by diminishing awareness of the disease, targeted AS that screens more high-risk individuals, and/or the introduction of new single-dose drug, acoziborole.”

Reviewer #3:

Objectives are clearly stated.
Study design is appropriate and has been used previously to address similar objectives.
Study population has been described appropriately.
Appropriate statistical analysis was employed.
No ethical concerns.

**Author response:** We thank the reviewer for their positive feedback.

--------------------

**Results**
Reviewer #1: The analysis presented matches the analysis plan. The results are clearly presented. The figures applied are of sufficient quality and quite clear.

**Author response:** We thank the reviewer for their positive feedback.

Reviewer #2: The results are well presented and clear and seem to me to be in line with the objectives and the method used.

**Author response:** We thank the reviewer for their positive feedback.

Reviewer #3: Results match the analysis plan as mentioned in the methods section.
Results are clearly presented.
Figures and Images are of sufficient quality for online publication, may need more polishing for printing purposes.

**Author response:** We thank the reviewer for their positive feedback. We have run all figures through PACE and confirmed that our main figures match the requirements.

--------------------

**Conclusions**
Reviewer #1:

There is no conclusion section. This needs to be worked on.
Public health relevance addressed, and authors attempt to discuss the usefulness of the model

**Author response:** We added a Conclusion section at the end of the manuscript (lines 582—618).

Reviewer #2:

We would have liked the authors to take into account in the discussion the aspect of the re-invasion of vectors, ... it has been demonstrated on several occasions that if vector control is not concerted and applied in other areas of surrounding health and/or provinces and/or endemic countries, the vectors concerned by the control recolonize the environments and can constitute a non-negligible factor in the resurgence of the disease. Moreover, during the lock-down the care activities did not stop and some mobile teams did little to work. The impact on disease control was therefore low. The aspect on the functionality of the health system is major in the control of HAT in areas with low endemicity. and this was not very elaborated in the discussion. Passive screening can also be impacted by the poor functionality of health centers. Some work has revealed that the rate of use in the two HAT endemic areas may be low and this may make it difficult to implement an effective HAT surveillance system based on passive screening. Sometimes the use of mini teams may be necessary. It is possible that despite the best scenarios even if the patient does not consult the health center, he cannot be diagnosed and treated and remains a danger for the members of his community.

**Author response:** We agree that the reinvasion of vector population is a non-negligible factor in the resurgence of the disease when the control measures have been paused for any reason. We added the following paragraph to make it clear to readers that we have considered the bounceback of tsetse populations during the interruption period in our model (line 321):

“We assume that, if VC deployments are interrupted, that there is some possibility for bounceback of the tsetse populations. We model the tsetse population explicitly and this bounceback is assumed to be driven by repopulation from remaining tsetse in the region (see S1 Text). In regions with planned roll out in 2020 or 2021 which are interrupted we assume these are then postponed to July 2021 and July 2022 in the 9- and 12-month scenarios respectively.”

The modelled bounceback dynamics under different interruption periods are added to the supplementary information (S1 Text: S1.3.3 Tsetse bounce back in interruption scenarios & Fig F).

We agree that PS is important yet can be challenging to implement effectively. We stated in the Methods section “Access to such facilities can be geographically heterogeneous, and in the case where no microscopy is available in the screening facility, if an initial RDT is positive, further travel is needed to enable the suspect to have infection confirmed. This can result in high levels of attrition between screening and confirmation [24]” and have now added the following text to the Discussion section (line 483):

“Our modelling results highlight the importance of the PS system in avoiding disease burden in the Bandundu Nord and Sud coordinations in the DRC, as noted when comparing strategies with continuing PS to those with reduced PS. This in spite of challenges linked to accessibility of fixed health centres, training of health facility staff, and local availability of diagnostics and drugs [24]. As well as direct burden benefits, a robust PS system will be essential moving into the future, as when vertical interventions (AS and VC) are ceased after having no case reporting for 5 years [13], PS will remain the main mode of monitoring the success of the programme or triggering reactive vertical interventions.”

Reviewer #3:

The conclusions are supported by the data presented.
No limitations have been explicitly mentioned.
The authors discuss the data and provide insights of its applicability in relation to the topic under study.
Public health relevance is appropriately addressed.

**Author response:** We have created a Limitations subsection in the Discussion section (lines 497—581). In which we discussed limitations of our study related to the model structure, assumptions, and data availability.

--------------------

**Editorial and Data Presentation Modifications?**
Reviewer #1:

presentation modifications include;
1. Clearly show the key paper headings i.e  Introduction, objectives, methodology, findings / results, discussion, conclusion & recommendations. + key guiding assumptions
2. Some important maps for inclusion have been suggested and are highly recommended
3. Model details are missing. Hard to follow and cause duplication.

**Author response:** We restructured our paper to include more key paper handles such as objectives and conclusion. To make our present study understandable as a standalone article, we also added extra content into the main text (in the Model subsection) and supplementary information (S1: Text) including a schematic illustration of our model (Fig A), prior and posterior distributions of geographical varying fitted parameters (Fig B), figures demonstrating how improved passive screening and vector control are modelled (Fig C—D and F), fitting results (Fig E) from our previous publication (Crump et al., 2020), and finally maps of our study areas (Fig G). However, we feel that it would be misleading to provide detailed maps of where AS, VC and PS are operating at a sub-health-zone level because we do not use smaller-scale spatial modelling in this paper (see our responses to comment #12 below).

Reviewer #2: minor revision

Reviewer #3: (No Response)

--------------------

**Summary and General Comments**
**Reviewer #1:**

| **Comment** | **Author response** |
| --- | --- |
| 1. What are 'health zones'? need to provide interpretation. | We added a “Study areas” subsection at the beginning of the Methods section and included an interpretation of health zones (lines 109—127). |
| 2. Why continuously use the term ‘former Bandundu province? Better to use the current identification. | We changed from using the terminology “former province” to “Bandundu Nord and Sud coordinations” to reflect the fact that operational planning takes place at the coordination level. We also clarify what this means in terms of current provinces for the DRC (line 114):  “We picked Bandundu Nord and Sud coordinations (which are the same geographical region as Kwilu, Mai Ndombe and Kwango provinces) as our study areas because these regions see some of the highest levels of on-going case reporting across the country. Furthermore, as there are health zones in these coordinations with low case reporting, and therefore we expected to see a range of impacts of interruptions across these different prevalence settings. A map showing the location of Bandundu Nord and Sud within the DRC and their relation to Kwilu, Mai Ndombe and Kwango provinces can be found in S1 Text: Fig G.” |
| 3. Update your information on countries which have been validated for elimination of gHAT and have since been declared so by WHO in this year 2022. | We revised the following paragraph to provide up to date information on countries that have been validated for EPHP of gHAT (line 28):  “Up to the end of 2022, five countries (Togo in June 2020, Côte d’Ivoire in December 2020, Benin in November 2021, Uganda in April 2022, and Equatorial Guinea in June 2022) have received official validation for the EPHP of gHAT [10—12].” |
| 4. Please state the objectives clearly and even conclusion. | We added a “Research objectives” subsection at the end of the Introduction section (lines 86—107) and a “Conclusion” section after the Discussion section (lines 582—618). |
| 5. Unfortunately, this is not a stand-alone piece of work as there is frequent reference to the ‘previous works’ even for aggregated data used for model fitting. Hence reviewer is compelled to refer to previous team works. | We understand the reviewer’s comment and have augmented both the main text (in the Model subsection) and the SI (S1: Text) with additional information (lines 203—258 in the main text, a schematic illustration of our model (Fig A), prior and posterior distributions of geographical varying fitted parameters (Fig B), figures demonstrating how improved passive screening and vector control are modelled (Fig C—D and F), fitting results (Fig E) from our previous publication (Crump et al., 2020), and finally maps of our study areas (Fig G) in the SI) which hopefully provides sufficient background to the model fitting and projections presented elsewhere without the need to read our team’s other publications in detail. We believe we have now included sufficient detail to make our present study understandable as a standalone article. Of course for full justification of assumptions and results we would still direct readers to the original research articles published elsewhere. |
| 6. Not clearly able to see the disruptive roles of covid, political unrest, disasters disease epidemics etc in the model. Their modes of disruption differ. | We agree that these different modes of disruption differ substantially, however we did not want to concretely link specific causes of interruption to specific changes in strategy implementation. We added the following paragraphs in the Methods section to clarify it (line 295):  “Different modes of disruption vary substantially, however, we did not want to concretely link specific causes of interruption to specific changes in strategy implementation. Therefore, our analyses are framed in the context of interruption to different intervention components, to determine what type and what severity of impact interruptions could have, regardless of cause. Motivated by the COVID-19 pandemic and interruption durations that were considered plausible in early 2020, we focus on interruptions starting in April 2020 lasting until either the end of 2020 or the end of 2021. Although the COVID-19 pandemic has had seemingly minimal impact on strategy — with only a reduction in the AS coverage for 2020 — it was not clear at the outset of this novel pathogen outbreak whether this would have more far-reaching consequences of the type experienced during the West African Ebola outbreak in 2013—2016 where the PS system was heavily impacted in addition to the AS coverage falling [19]. Finally, political unrest could impact all intervention types, but this would be highly dependent on the nature, location, and duration of the situation.” |
| 7. Active Screening coverage is not uniform due to several reasons. How have you accounted for this? | We used historical screening data (2000—2016) during fitting and assumed random participation in AS from the low-risk group only. The reasons for having this assumption on risk and AS participation structures are given in lines 218—226:  “Previous development of this model has found that heterogeneity in the risk of people being bitten is important to be able to replicate time-series trends observed in reported case data for different settings [21,26,30], and furthermore the best fitting models had high-risk people that did not participate in AS, which also aligned with anecdotal evidence. In the present study we therefore consider that our low-risk group of people participate in AS randomly and according to the coverage of annual AS, but our high-risk group do not participate and can only be detected and treated by seeking help through the PS system in fixed health facilities.”  For future screening, the mean value is based on the most recent screening data (i.e. 2014—2018) available to us. To highlight these assumptions, we added the following paragraphs in the Methods and Discussion sections (lines 146 and 575 respectively):  “Our fitted model explicitly uses the number of people tested during AS for 2000—2016, which captures the historical variation in AS coverage. Despite the potential for decreasing interest in attending AS when knowledge of disease is diminishing, data shows that the maximum number of people screened occurred after 2016 in 19 of out 38 health zones in Bandundu Nord and Sud coordinations. Our future projections use the average of known 2014—2018 AS coverage (the 2014–2016 HAT Atlas and the 2017–2018 PNLTHA-DRC AS data) for each health zone as the future coverage to reflect the observed trends in participation in recent years.”  “The medium- to long-term projections could be influenced by model assumptions, it would also be possible to refit the model to the actual screening and case data to quantify if there is any statistical evidence of the interruption's impact on PS during 2020. Additionally, we can also factor in some plausible changes such as the decrease in AS participation caused by diminishing awareness of the disease, targeted AS that screens more high-risk individuals, and/or the introduction of new single-dose drug, acoziborole.” |
| 8. The model was fitted on the 2000-2016 HAT Data yet covid data is only for 2020-2021. How was this harmonized? | Model fitting, via a MCMC method, was used to parameterise the model independently in the different health zones. The realised parameter sets are then used to project forwards in time, applying the various intervention strategies described in the manuscript. The interruption occurs during this projection phase and no actual COVID-19 data in the DRC was used in this study. We added the following paragraph in the Methods section to clarify this (line 254):  “The posterior parameter sets for each health zone are then used to project forwards in time, applying various intervention settings described below in Baseline strategy (no interruption) and Interruption scenarios. In this study, all interruption scenarios occur during this projection phase and no actual data other than the timing of the start of COVID-19 epidemic in the DRC was used in our projections.” |
| 9. Vector control is not everywhere in your ‘health zones’ – I imagine so. What is the strength of the model outputs in such a scenario? What population is being effectively protected from tsetse bites by the Tiny targets? | The national programme in the DRC (PNLTHA-DRC) and the expert team of tsetse control from Liverpool School of Tropical Medicine (LSTM) mapped out deployment areas based on historical reported cases. In this study, we assumed the entire population in the health zones is protected by VC, but we are currently developing methods to incorporate effective VC coverage into our model. We added the following paragraph in the Discussion section to state our model assumption and expectation when using realistic VC coverage (lines 556 & 560):  “The levels of protection from VC can be highly variable within a health zone depending on the geographical distribution of Tiny Targets. In this study, we assumed VC covers 100% of population at risk.” & “To improve the tsetse dynamics generated by our model, in the future we hope to combine case coverage of VC and observed VC efficiency (within intervention areas) to estimate the overall tsetse reduction in a health zone. Note that simulation results would be very different if a much lower overall reduction percentage (e.g. 20%) were fed into our model, although we believe these low reduction values are very unlikely.” |
| 10. Tiny targets are laid along major river banks to trap the riverine glossina species. But it is also true that during the rainy season tsetse flies are more wide spread far beyond the river banks. How have you accounted for this? | We did not consider any seasonal effects in our model, and therefore, we added a sentence to the Discussion section to make it clear that we did not take seasonal factors into account (line 558):  “Other factors such as seasonal distribution of tsetse populations and tsetse reinvasion from connected areas are also out of the scope of this paper.” |
| 11. Vector control effectiveness has been standardized to 80% across board even where it has never been applied. Under such circumstances am not sure if this VC carries with it any role in the model. Also refer to lines 440 and 441. Why bother with VC? Looks like a redundant model parameter. | We have standardised to 80% tsetse reduction for health zones other than Yasa Bonga (where a known 90% reduction was achieved) as this mirrors a similar reduction achieved in other locations. In lines 440 and 441 we meant that reductions at a similar level (e.g. >70% tsetse reduction) would have similar impacts on transmission to humans and case reporting as the default values presented, however simulating with a much lower reduction percentage (e.g. 20%) would certainly generate very different looking results. We have modified our text in the Discussion section to be clearer that the tsetse reduction parameter is important and certainly there is a large difference in simulating these models with and without VC (lines 543 & 563):  “At present there is limited data for tsetse reduction in four of five health zones with VC prior to 2020 as they have started recently with six-monthly monitoring. As such we assumed 80% tsetse reduction after one year, which is less than the observed 90% in Yasa Bonga health zone and Uganda [28], or 99% in Chad [21,26] and on par with Boffa in Guinea [27]. Once entomological data from the health zones is available we can appropriately adjust the simulated VC reduction. However previous studies have shown that a reduction in tsetse of >70% could break transmission [28] and that reductions of this magnitude should be sufficient to interrupt transmission within 4 years [29]. In the DRC Tiny Target deployments are focused on the rivers that serve as the likely tsetse habitats for villages that have reported cases. So, whilst the VC is not exhaustive and is not designed to eliminate tsetse, it is aimed to substantially reduce those populations that are maintaining transmission cycles. Therefore, we expect limited differences in modelled outcomes when replacing the assumed tsetse reduction by observed ones when data becomes available.” & “Note that simulation results would be very different if a much lower overall reduction percentage (e.g. 20%) were fed into our model, although we believe these low reduction values are very unlikely.” |
| 12. There was need to set the stage with providing a spatial connectivity for the following; areas being treated with tiny targets (deployment sites), AS screening sites, PS (health centres), population densities (gridded). This should be map(s). | Since our model is operating here at the level of health zones it utilises aggregate health zone information for AS coverage, any case detections, and whether or not VC was operating or was planned. The PS coverage is estimated through model fitting to the data specific to the health zone and therefore provides a health-zone-level value for stage 1 and stage 2 case detection rates. It cannot tell us at a more granular level how effective different health facilities are but does use historical data to provide the overall health-zone-specific picture of the strength of on-going PS. In the model we do not use smaller-scale spatial modelling and therefore we feel that it would be misleading to provide detailed maps of where AS, VC and PS are operating at a sub-health-zone level. We do note that VC deployments by PNLTHA and LSTM are heavily guided by recent case reporting and so we would expect there to be strong correlation with where there is risk and where VC deployments take place. |
| 13. What was particularly unique with Bandundu setting? Any lessons picked? Can this model be automatically duplicated elsewhere? | Despite the fact that this analysis can be applied to any locations with sufficient data (> 10 data points in order to perform model fitting), we picked the former Bandundu province (now we use Bandundu Nord and Sud coordinations to refer to the same areas) to be our study area because we expect to see a diversity of impacts due to the widest variety of risk categories (very low, low, and medium) that it covers. We added the following paragraph into the Methods section to explain why we pick this specific setting (line 114):  “We picked Bandundu Nord and Sud coordinations (which are the same geographical region as Kwilu, Mai Ndombe and Kwango provinces) as our study areas because these regions see some of the highest levels of on-going case reporting across the country. Furthermore, as there are health zones in these coordinations with low case reporting, and therefore we expected to see a range of impacts of interruptions across these different prevalence settings. A map showing the location of Bandundu Nord and Sud within the DRC and their relation to Kwilu, Mai Ndombe and Kwango provinces can be found in Fig G.” |
| 14. The unique stakeholder inputs are not highlighted anywhere (and in what ways where they involved?) | We agree that stakeholder inputs are really important. Although this study is not designed to provide direct recommendations to stakeholders, we believe it gives valuable insights into consideration of unforeseen or unplanned interruptions, and how concerning these might be for stakeholders. This study reflects a small piece in an on-going body of work between the modelling group (HAT MEPP) and national programme (PNLTHA-DRC) and experts in tsetse control from LSTM – there are members of both organisations as co-authors for this study. We have regular discussions about on-going and planned strategies and new data analyses with these teams and other stakeholders (e.g. WHO and researchers in the area of drugs and diagnostics). We added the following paragraph in the Methods section to redirect readers to S3 Text: PRIME-NTD criteria where we provided more explicit information (line 198):  “This model has been developed and refined over many years in conjunction with PNLTHA-DRC and a variety of other stakeholders to ensure the modelling team fully understand historical data and context, capture the known epidemiology, and account for region-specific interventions within the model framework. We give a more specific list of stakeholder inputs and interactions in S3 Text.” |
| 15. The presence of an open source code is good and could be used for model validation as new actual data becomes available. | We agree. |

**Reviewer #2:** (No Response)

**Reviewer #3:** This paper presents a comprehensive exploration of the potential impact of interruptions towards the end of transmission and burden of Gambiense human African trypanosomiasis (gHAT) in thirty-eight health zones in the Democratic Republic of the Congo (DRC). The authors employed an existing deterministic model (implemented in MATLAB) to  simulate the transmission and reporting dynamics of gHAT under six scenarios of interruptions to intervention efforts for periods of nine and twenty-one months. The obtained predictions suggest that simultaneous interruption of passive and active screening could substantially increase  the burden of gHAT as compared to interrupting active screening alone. 
The paper is well-written, and the presented insights are interesting. However, there are some minor points that need clarification.

| **Comment** | **Author response** |
| --- | --- |
| 1. The specifications of the computational infrastructure platform used to conduct the analysis must be reported indicating CPU/GPU, RAM, OS among others. In addition, the authors should present the runtime analysis, indicating how much compute time is required to run simulations under different interruption scenarios on the employed compute infrastructure. This is important not only for reproducibility purposes but also compute cost estimation for this kind of analysis. | Our projections are running in parallel using the built-in function “parfor” in MATLAB. The runtime provided below is highly dependent on how many cores you assign to the code when running the simulations. With that in mind, we added a “Computational specifications and runtime” section to the supplementary information to provide information of our machines and runtime for different pieces of analyses (S1.5 in S1 Text) as below:  “Our code, available in Open Science Framework at https://osf.io/gur7c/, has been tested and run in MATLAB R2018b, R2021b, and R2022b on Windows, Linux and Mac operating systems. Model fitting for a single health zone takes 12–22 hours depending on data quality (how many data points present and possibly missing active screening numbers) and intervention history (which dictates the number of fitted parameters, e.g. improved passive detection has four extra parameters) on our cluster. In a subset of health zones additional running is required to ensure that the two MCMC chains have converged to the same volume of parameter space and to achieve our target effective sample size of 1,000. In this situation, the total elapsed time across runs for a health zone can increase greatly (up to 3 or 4 times the initial run time). Two independent MCMC chains were run in parallel for each MCMC analysis and we assigned 3 cores (1 per chain, plus a master/controller) with 5 GB RAM per CPU for each fitting job on our cluster. The cluster is running CentOS Linux version 7.8 and has either Intel Xeon Gold 6130 (2.10GHz) or Intel Xeon Gold 5218 (2.30GHz) CPUs depending on the node used. Future projections of 1,000 realisations with 10 samples each from 2020 to 2050 for seven strategies (one baseline and six interruption scenarios) take about 20 minutes to complete on a desktop machine (MacOS, 4.2 GHz Quad-Core, 32 GB 2300 MHz DDR4).” |
| 2. Page 6, line 195 and Table 1, column 5;  The authors assume “a fixed effectiveness of 80% tsetse reduction in one year - the MEanAS+VC baseline strategy”.  The authors need to justify the choice of 80% for this parameter. | We have standardised to 80% tsetse reduction for health zones other than Yasa Bonga (where a known 90% reduction was achieved) as this mirrors similar reduction achieved in other locations. We have modified our text in the Discussion section to be clearer that the tsetse reduction parameter is important and certainly there is a large difference in simulating these models with and without VC (lines 543 & 563):  “At present there is limited data for tsetse reduction in four of five health zones with VC prior to 2020 as they have started recently with six-monthly monitoring. As such we assumed 80% tsetse reduction after one year, which is less than that the observed 90% in Yasa Bonga health zone and Uganda [28], or 99% in Chad [21,26] and on par with Boffa in Guinea [27]. Once entomological data from the health zones is available we can appropriately adjust the simulated VC reduction. However previous studies have shown that a reduction in tsetse of >70% could break transmission [28] and that reductions of this magnitude should be sufficient to interrupt transmission within 4 years [29]. In the DRC Tiny Target deployments are focused on the rivers that serve as the likely tsetse habitats for villages that have reported cases. So, whilst the VC is not exhaustive and is not designed to eliminate tsetse, it is aimed to substantially reduce those populations that are maintaining transmission cycles. Therefore, we expect limited differences in modelled outcomes when replacing the assumed tsetse reduction by observed ones when data becomes available.” & “Note that simulation results would be very different if a much lower overall reduction percentage (e.g. 20%) were fed into our model, although we believe these low reduction values are very unlikely.” |
| 3. Model parameters for the simulation are shown, well referenced, and justified. However, it would great to provide a sensitivity analysis of parameters to check the effect of different choice of model parameters to the model predictions. | A previous work from our group (Aliee et al, PLoS Com. Bio., 2021) performed a full sensitivity analysis for this model (called “Model 4” or “baseline model”). Using a ranked partial correlation method, the results showed that our fitted parameters (which we have most uncertainty about) are the ones leading to most variation in outcomes. We added the following paragraph in the Methods section to provide readers with a reference (line 229):  “There are two types of parameters in our model, fixed and fitted parameters. Most fixed parameters are biological parameters, and therefore their values are most likely to be location-independent (health zones in our study) with less uncertainty. Fitted parameters, on the other hand, are believed to vary from location to location with much bigger uncertainty so we fit these on a health zone by health zone basis. A full sensitivity analysis performed elsewhere for this same model [31] used a ranked partial correlation method and found that the fitted parameters were the ones leading to most variation in outcomes, strengthening our justification for which parameters should be fixed and fitted.” |
| 4. In the discussion section, the authors cite previous work that showed similar predictions between deterministic and stochastic versions of the model. However, it is mentioned that for this work, “a stochastic version of the model could better capture the model”. Why not present the stochastic version of the model? The authors could clarify why a deterministic version is presented if a stochastic version could provide better insights. | The previously used stochastic version of the model was not coded to optimise its speed. A revised version is under development that will be more practical to use across a large number of locations. The stochastic version of the model becomes more valuable at low prevalence and in smaller geographical areas (e.g. health areas or villages). In Bandundu Nord and Sud coordinations, which have higher prevalence on average than most other regions, we do not believe that use of the stochastic model would result in different conclusions. Finally, we don’t expect there to be great variation between the disease burden estimated in the two model types given the similarities in case reporting predictions even at low values.  We revised the following paragraph to clearly explain why we chose to use the deterministic model knowing that the stochastic one could be preferable for studying elimination of transmission (line 501):  “Although stochastic models are generally better suited to capturing dynamics approaching elimination, we do not expect this variant of the deterministic model (representing average expected dynamics) to result in different conclusions from a stochastic model, as other work by this group demonstrated similar predictions generated by the analogous stochastic gHAT model [20]. In this study we chose to utilise the deterministic version as we expected limited impact on key outcomes (change in disease burden with interruptions and delay in elimination year) and to avoid the comparative high computational cost of running a stochastic model. If a similar analysis was conducted for smaller spatial scales (e.g. a health area with around a tenth of the population size of a health zone) then stochastic models would become more desirable.” |
